# N6-methyladenosine writer METTL16-mediated alternative splicing and translation control are essential for murine spermatogenesis

**DOI:** 10.1186/s13059-024-03332-5

**Published:** 2024-07-19

**Authors:** Qian Ma, Yiqian Gui, Xixiang Ma, Bingqian Zhang, Wenjing Xiong, Shiyu Yang, Congcong Cao, Shaomei Mo, Ge Shu, Jing Ye, Kuan Liu, Xiaoli Wang, Yaoting Gui, Fengli Wang, Shuiqiao Yuan

**Affiliations:** 1 Department of Urology, Shenzhen Key Laboratory of Male Reproductive Medicine and Genetics, Peking University Shenzhen Hospital, Shenzhen, Guangdong 518036 China; 2 Institute of Reproductive Health, Tongji Medical College, Huazhong University of Science and Technology, Wuhan, 430030 China; 3 Laboratory Animal Center, Huazhong University of Science and Technology, Wuhan, Hubei 430030 China; 4 Shenzhen Huazhong University of Science and Technology Research Institute, Shenzhen, 518057 China

## Abstract

**Background:**

The mitosis-to-meiosis switch during spermatogenesis requires dynamic changes in gene expression. However, the regulation of meiotic transcriptional and post-transcriptional machinery during this transition remains elusive.

**Results:**

We report that methyltransferase-like protein 16 (METTL16), an N6-methyladenosine (m6A) writer, is required for mitosis-to-meiosis transition during spermatogenesis. Germline conditional knockout of *Mettl16* in male mice impairs spermatogonial differentiation and meiosis initiation. Mechanistically, METTL16 interacts with splicing factors to regulate the alternative splicing of meiosis-related genes such as *Stag3*. Ribosome profiling reveals that the translation efficiency of many meiotic genes is dysregulated in METTL16-deficient testes. m6A-sequencing shows that ablation of METTL16 causes upregulation of the m6A-enriched transcripts and downregulation of the m6A-depleted transcripts, similar to *Meioc* and/or *Ythdc2* mutants. Further in vivo and in vitro experiments demonstrate that the methyltransferase activity site (PP185-186AA) of METTL16 is necessary for spermatogenesis.

**Conclusions:**

Our findings support a molecular model wherein the m6A writer METTL16-mediated alternative splicing and translation efficiency regulation are required to control the mitosis-to-meiosis germ cell fate decision in mice, with implications for understanding meiosis-related male fertility disorders.

**Supplementary Information:**

The online version contains supplementary material available at 10.1186/s13059-024-03332-5.

## Background

Spermatogenesis is a highly complicated and delicate process involving mitosis of spermatogonia, meiosis of spermatocytes, and spermiogenesis. In mice, gonocytes resume mitosis at approximately 1.5 or 2 days after birth and form spermatogonial stem cells (SSCs) [1]. Some SSCs maintain their stemness, whereas others subsequently differentiate into spermatogonia (types A and B), giving rise to primary spermatocytes, which undergo leptotene (formation of programmed DNA double-stranded breaks), zygotene (initiation of synapsis between homologous chromosomes), pachytene (completion of synapsis), diplotene (unsynapsis and dissolved crossover), and diakinesis to complete meiotic prophase I [2]. One primary spermatocyte produces four round haploid spermatids after two successive meiotic events [3]. Although gene expression in germ cells at different stages is strictly regulated at the transcriptional and post-translational levels, the detailed mechanisms remain unclear.

N6-methyladenosine (m6A) is the predominant mRNA modification and participates in mRNA splicing [4], stability [5], and translation [6]. m6A abundance is dynamically regulated by m6A writers, readers, and erasers: writers of non-rRNA include methyltransferase-like protein 3 (METTL3)/METTL14, WT1 associated protein (WTAP), and METTL16; readers include YTH N6-methyladenosine RNA binding protein C1 and 2 (YTHDC1/2), YTHDF1/2/3, and proline rich coiled-coil 2A (PRRC2A); and erasers include AlkB homolog 5 (ALKBH5) and FTO alpha-ketoglutarate dependent dioxygenase (FTO) [7–9]. This writer-reader system-controlled gene expression is essential for embryonic development in mice [8] and sex determination in *Drosophila* [10]. The METTL3/METTL14 complex is the main m6A writer involved in spermatogonial differentiation, meiosis initiation, and spermiogenesis via translational regulation in an m6A-dependent manner [11, 12]. Interestingly, *Mettl3/Mettl14* double knockout via *Stra8* (*stimulated by retinoic acid 8*)*-Cre* in the mouse testis, which specifically inactivates both *Mettl3* and *Mettl14* in germ cells starting from P3, resulted in normal meiosis and impaired spermiogenesis, with negligible changes of m6A levels in spermatocytes and significant reduction of m6A levels in spermatids [11], suggesting that other m6A methyltransferase members might exist to regulate m6A during meiosis.

Human METTL16 is encoded by 562 amino acids, with the methyltransferase domain (MTD) and N-terminal region forming a deep-cut groove that binds to the substrate RNA [13, 14]. In contrast to the numerous mRNA substrates of METTL3/METTL14, the substrates of METTL16 mainly include methionine adenosyltransferase 2A (*Mat2a*) and several noncoding RNAs [15–17]. Global knockout of *Mettl16* causes embryonic lethality due to the reduced expression of the S-adenosylmethionine (SAM) synthetase *Mat2a* in 16-cell embryos [8]. Additionally, METTL16 exists in the cytosol and directly interacts with eukaryotic translation initiation factor 3 subunit A (eIF3a), eIF3b, and ribosomal RNA to promote m6A-independent translation [18]. In U2OS osteosarcoma cells, METTL16 inhibits the exonuclease activity of MRE11 homolog (MRE11) by forming a complex with RNA in a methyltransferase-independent manner, thus repressing DNA end resection during double-strand break (DSB) repair [19]. A recent study showed that METTL16 is essential for genome integrity during erythropoiesis via the regulation of BRCA2 DNA repair associated (*Brca2*) and FA complementation group M (*Fancm*) mRNA expression in a methyltransferase-dependent manner [20]. Interestingly, in *Caenorhabditis elegans*, METT10 (the ortholog of mouse METTL16) could deposit m6A modification on the 3’-splice site (AG) of the SAM synthetase pre-mRNA to prevent the recognition by the essential splicing factor U2 small nuclear RNA auxiliary factor 35 (U2AF35) [16]. In addition, germ cell-specific knockout of mouse *Mettl16* generated by *Vasa*-Cre (induces recombination in germ cells as early as embryonic day 15) exhibited male infertility with atrophied testes [16]. However, the specific functions and mechanisms of METTL16 in mammalian spermatogenesis are yet unclear.

In the present study, we used *Stra8*-Cre (induce recombination at postnatal day 3, since type A1 spermatogonia) to construct a germ cell-specific knockout mouse model of *Mettl16* to investigate the physiological role of METTL16 during spermatogenesis. METTL16 deficiency in male germ cells causes a reduction in differentiated spermatogonia and compromised DNA replication before entering the meiotic prophase, which ultimately lead to male infertility. Further studies showed that METTL16 could directly bind to the mRNA of *Stag3* (*STAG3 cohesin complex component*) and coordinately regulate the alternative splicing (AS) by recruiting splicing factors 3b subunit 1 (SF3B1) and SF3B3. Mechanistically, we discovered that METTL16 could regulate gene transcription and translation efficiency (TE) in mouse testes by direct targeting and that METTL16 modulated the translation efficiency of mRNA transcripts with no m6A bias in testicular cells. Furthermore, m6A-sequencing (m6A-seq) identified several differentially methylated transcripts in *Mettl16* conditional knockout (cKO) testes and demonstrated that the methyltransferase activity site of METTL16 is required for normal spermatogenesis in mice. In addition, we showed that METTL16 interacts with the meiosis specific with coiled-coil domain (MEIOC)/YTHDC2/RNA binding motif protein 46 (RBM46) complex and is involved in spermatogenesis. Thus, our study revealed a previously undefined mechanism that modulates gene expression in spermatogonial differentiation and meiosis initiation, highlighting the crucial role of METTL16 in spermatogenesis to ensure male fertility.

## Results

### METTL16 is highly conserved and essential for spermatogenesis in mice

**Fig.1:**
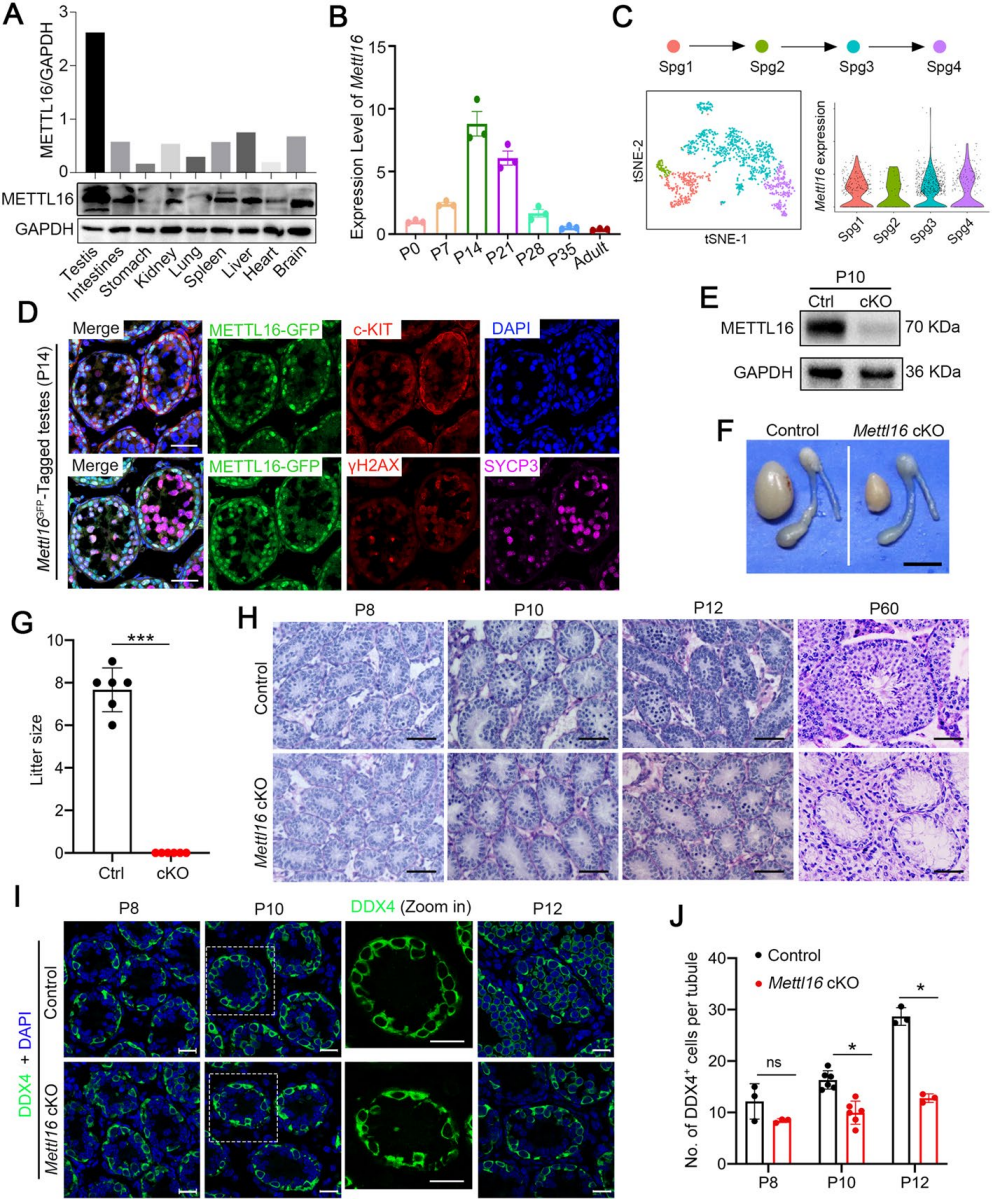
Germline-specific knockout *Mettl16* in male mice impairs spermatogenesis and male fertility. **A** Expression of METTL16 in various tissues of adult mice (8 weeks) is detected by Western blot. GAPDH was used as a loading control. **B** Expression of *Mettl16* in the developmental testes of mice is assessed through RT-qPCR. *Gapdh* was used for norminalization. Data were presented as mean ± SEM, *n* = 3 (three biological replicates). **C** scRNA-seq analyses of the expression profile of *Mettl16* during spermatogonia differentiation is shown. Spg1, Spg2, Spg3, and Spg4 represent SSCs, undifferentiated spermatogonia, differentiating spermatogonia, and differentiated spermatogonia, respectively. **D** Expression of METTL16-EGFP (green) in testes from P14 *Mettl16*^EGFP^-tagged mice is detected by immunofluorescent staining with marker proteins, including c-KIT (red), or SYCP3 (purple) and γH2AX (red). DAPI (blue) was used to counterstain the nuclei. Scale bars = 20 μm. **E** METTL16 expression in Control and *Mettl16* cKO mice at P10 is analyzed by Western blot. GAPDH was used as a loading control. **F** Gross images of testes from adult Control and *Mettl16* cKO mice are shown. Scale bar = 50 mm. **G** Histogram shows the litter size of adult Control (Ctrl) and *Mettl16* cKO (cKO) male mice. Data were presented as mean ± SEM, *n* = 6. ***P < 0.001. **H** PAS staining of testes from Control and *Mettl16* cKO mice at P8, P10, P12, and P60. Scale bars = 50 μm. **I** Immunofluorescent staining of germ cell marker (DDX4, green) on testicular sections from Control and *Mettl16* cKO mice at P8, P10, and P12. DAPI was used to counterstain the nuclei. Scale bars = 20 μm. **J** The histogram shows the quantification of the number of DDX4^+^ cells per tubule in (**I**). Data were presented as mean ± SEM. *n* = 3 for P8 mice, *n* = 6 for P10 mice, and *n* = 3 for P12 mice. ns, not significant. *P < 0.05

Since METTL16 has been reported to be highly conserved in vertebrates [21], we performed multi-alignment and phylogenetic analyses of METTL16 orthologs in six vertebrate species. Consistently, METTL16 was highly conserved across species, and human METTL16 shared 89.15% of the amino acid sequence with its mouse orthologs (Additional file 1: Fig. S1A). In addition, the N-terminus of the METTL16 protein was more conserved than the C-terminus, suggesting a high conservation of the MTD (Additional file 1: Fig. S1B). To explore whether METTL16 plays a role in the reproductive system, we first detected the expression of METTL16 in various mouse organs by Western blotting. As shown in Fig. 1A, METTL16 was predominantly expressed in the adult mouse testes, indicating that METTL16 may function in spermatogenesis. We then examined the mRNA levels of *Mettl16* in developing mouse testes by RT-qPCR. The results showed that the expression of *Mettl16* mRNA gradually increased after birth, reaching its highest level at postnatal day 14 (P14), and then decreased to a low level in adult mouse testes (Fig. 1B). Further analysis of *Mettl16* expression in adult testes using previously published single-cell RNA-sequencing (scRNA-seq) datasets [22] revealed that *Mettl16* mRNA was predominantly expressed in spermatogonia (Additional file 1: Fig. S1C). We next analyzed the *Mettl16* expression level among the four P7 spermatogonia clusters[23]: SSCs (SPG1); undifferentiated spermatogonia expressing *Utf1*, *Sall4,* and *Plzf* (SPG2); early differentiating spermatogonia expressing *Kit*, *Stra8,* and *Dnmt3b* (SPG3); and differentiated spermatogonia (SPG4). The results showed that *Mettl16* was ubiquitously highly expressed in the four clusters of spermatogonia (Fig. 1C), suggesting a potential role for METTL16 in spermatogonial self-renewal and differentiation.

Since the commercially available METTL16 antibodies did not work by immunofluorescence (IF), we generated *Mettl16-*EGFP-tagged transgenic mice via CRISPR/Cas9 strategy to study the subcellular localization of METTL16 in the testicular cells. IF staining with GFP antibody revealed that METTL16 is highly expressed in testicular cells at P14 mice, with more intense signals observed in WT1^+^ Sertoli cells, c-KIT^+^ spermatogonia, early spermatocytes with dot-like SYCP3, and weak signals in pachytene spermatocytes (Fig. 1D, Additional file 1: Fig. S1D). In addition, METTL16-GFP signals were also seen in PLZF^+^ undifferentiated spermatogonia of adult mouse testes (Additional file 1: Fig. S1D).

To determine the function of METTL16 in male germ cell development and spermatogenesis, we generated a germline conditional *Mettl16* knockout mouse model (*Mettl16* cKO) by breeding *Mettl16*^*flox/flox*^ mice with *Stra8-*Cre mice (Additional file 1: Fig. S2A-B). Western blot analysis of METTL16 protein levels in testes showed that METTL16 protein levels were significantly reduced in both P8 and P10 *Mettl16* cKO testes compared to control testes, and there was no truncated protein of METTL16 in *Mettl16* cKO testes (Fig. 1E, Additional file 1: Fig. S2C), suggesting METTL16 was successfully knocked out specifically in the testes. Interestingly, adult male *Mettl16* cKO mice were viable and appeared as healthy as control mice. However, the size of their testes was much smaller than those of the controls (Fig. 1F). Fertility tests showed that *Mettl16* cKO male mice were completely infertile (Fig. 1G), suggesting that spermatogenesis was disrupted in *Mettl16* cKO male mice.

To trace the morphological changes in *Mettl16* cKO testes during spermatogenesis, we performed Periodic acid–Schiff (PAS) staining of testes from P8, P10, P12, and P60 mice. Compared to the control mice, apparent germ cell loss was observed in the seminiferous tubules of P12 *Mettl16* cKO testes, and almost no germ cells were found in the inner layers of seminiferous tubules in P60 *Mettl16* cKO testes (Fig. 1H). Immunofluorescent staining using the germ cell marker DDX4 (DEAD-box helicase 4) confirmed a significant reduction in germ cells from P10 and a remarkable loss of DDX4-positive germ cells in the testes of adult *Mettl16* cKO mice compared with that of the control mice (Fig. 1I-J, Additional file 1: Fig. S2D-E). However, the number of PLZF-positive germ cells was comparable between adult control and *Mettl16* cKO mice (Additional file 1: Fig. S2F-G), suggesting that SSCs were not affected and still harbored the capacity to constantly produce new spermatogonia. In addition, the PNA signals in *Mettl16* cKO testes compared with control mice implied that meiosis was defective, and no post-meiotic germ cells were present (Additional file 1: Fig. S2H). Taken together, these results indicate that METTL16 is indispensable for male germ cell development and spermatogenesis in mice.

### METTL16 deficiency causes defective spermatogonial differentiation and meiosis initiation

**Fig.2:**
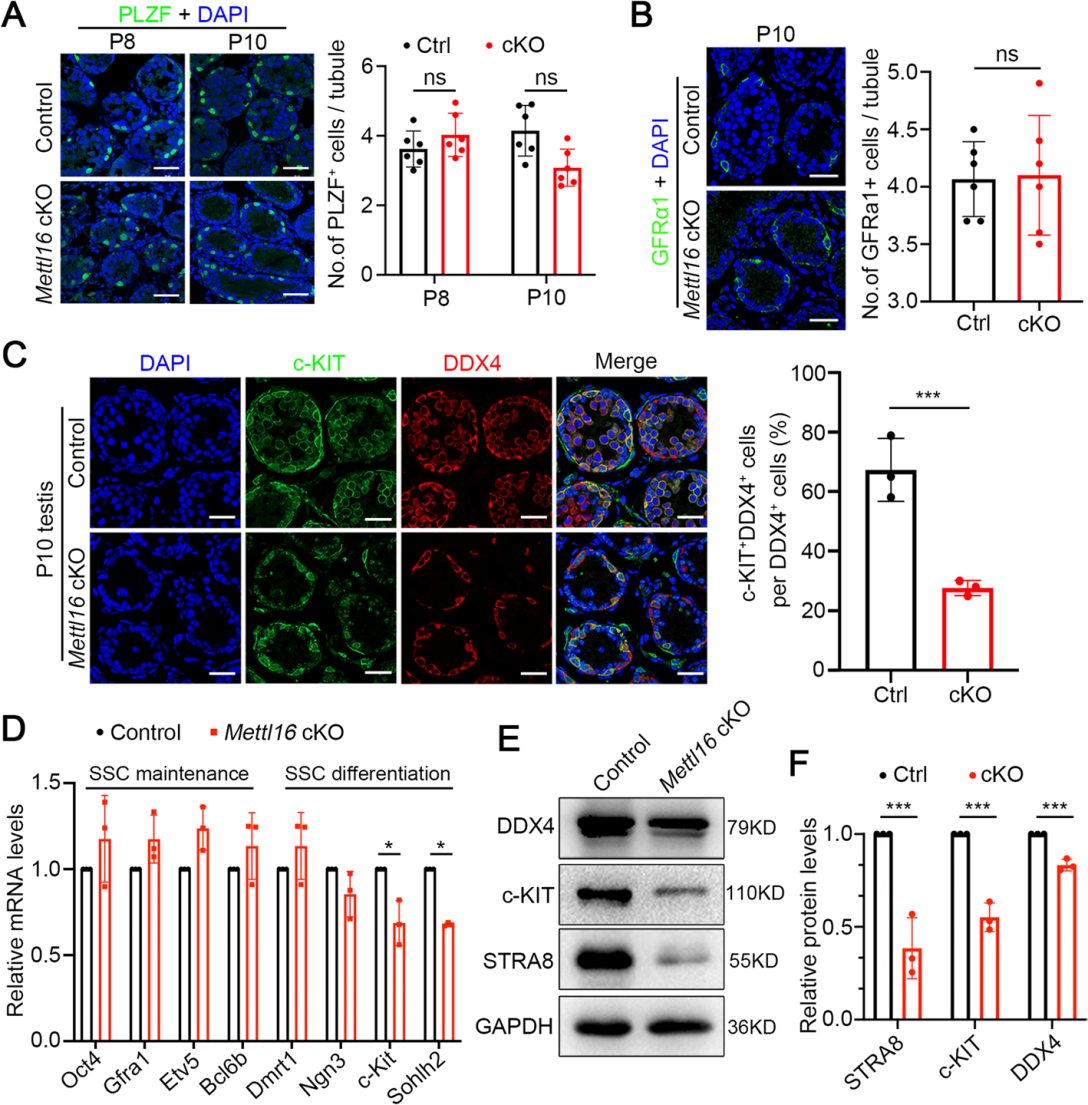
Ablation of METTL16 in mice causes aberrant spermatogonia differentiation. **A** Representative images of immunofluorescent staining for PLZF on testicular sections from Control (Ctrl) and *Mettl16* cKO (cKO) mice at P8 and P10 are shown. The DNA was stained with DAPI. Scale bars = 50 μm. The right histogram shows the quantification of the number of PLZF^+^ cells per tubule in (**A**). Data were presented as mean ± SEM. *n* = 6. ns, not significant. **B** Representative images of immunofluorescent staining for GFRα1 on testicular sections from Control and *Mettl16* cKO mice at P10 are shown. The DNA was stained with DAPI. Scale bars = 50 μm. The right histogram shows the quantification of the number of GFRα1^+^ cells per tubule in (**B**). Data were presented as mean ± SEM. *n* = 6. ns, not significant. **C** Representative images of co-immunofluorescent staining of c-KIT (green) and DDX4 (red) on P10 testicular sections from Control and *Mettl16* cKO mice are shown. Scale bars = 50 μm. The right histogram quantifies the ratio of c-KIT^+^ DDX4^+^ cells to DDX4^+^ cells in (**C**). **D** Histogram shows the expression of genes involved in SSC maintenance and differentiation in P10 testes from Control and *Mettl16* cKO mice. *Gapdh* was used for nominalization. Data were presented as mean ± SEM, *n* = 3. *P < 0.05. **E** Western blotting analyses of DDX4, c-KIT, and STRA8 in P10 testes from Control and *Mettl16* cKO mice. GAPDH was used as a loading control. **F** Histogram shows the quantification of the protein expression in (**E**). Data were presented as mean ± SEM, *n* = 3. ***P < 0.001

Considering the constitution of germ cells and the significant loss of DDX4-positive germ cells in *Mettl16* cKO testes at P10 and P12, we speculated that METTL16 deficiency may have caused abnormal spermatogonial differentiation or meiosis. To test this hypothesis, we first examined whether spermatogonial differentiation was affected in the *Mettl16* cKO mice through immunostaining for SSCs marker GFRα1, undifferentiated spermatogonia marker PLZF, and differentiated spermatogonia marker c-KIT. The results showed that the number of GFRα1- and PLZF-positive cells in *Mettl16* cKO mice was comparable to that of control mice, but the number of c-KIT-positive cells was significantly reduced in *Mettl16* cKO mice at P10 compared with controls (Fig. 2A-C). In addition, we found that the expression of genes involved in SSC maintenance (*Oct4*, *Gfra1*, *Etv5*, and *Bcl6b*) were similar to the controls, while the expression of *Sohlh2*, which participates in SSCs differentiation, was significantly decreased in P10 testes of *Mettl16* cKO mice compared to control mice (Fig. 2D). Western blotting also showed a remarkable reduction in DDX4, c-KIT, and STRA8 expression in *Mettl16* cKO testes at P10 compared with control mice (Fig. 2E-F). Together, these results suggest that METTL16 is essential for spermatogonia differentiation.

**Fig.3:**
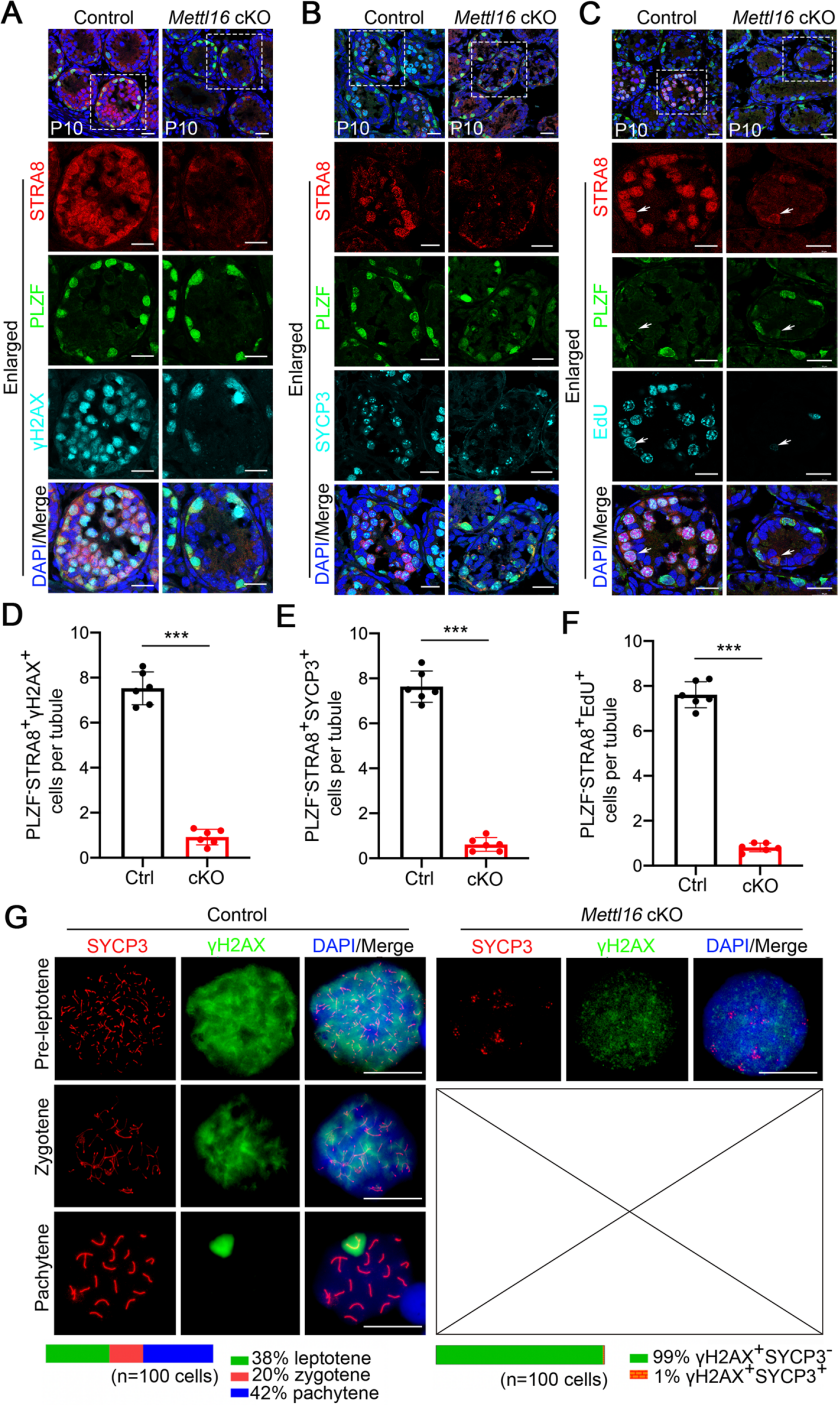
Male germline conditional knockout *Mettl16* results in meiosis arrested. **A** Representative images of co-immunofluorescent staining of STRA8 (red), PLZF (green), and γH2AX (cyan) on testicular sections from Control (Ctrl) and *Mettl16* cKO (cKO) mice at P10 are shown. The DNA was stained with DAPI. Scale bars = 20 μm. **B** Representative images of co-immunofluorescent staining of STRA8 (red), PLZF (green), and SYCP3 (cyan) on testicular sections from Control and *Mettl16* cKO mice at P10 are shown. The DNA was stained with DAPI. Scale bars = 20 μm. **C** Representative images of co-immunofluorescent staining of STRA8 (red), PLZF (green), and EdU (cyan) on testicular sections from Control and *Mettl16* cKO mice at P10 are shown. The DNA was stained with DAPI. Scale bars = 20 μm. **D**-**F** The histogram shows the quantification of the number of PLZF^−^STRA8^+^γH2AX^+^, PLZF^−^STRA8^+^SYCP3^+^, PLZF^−^STRA8^+^EdU^+^ cells per tubule in (**A**-**C**). Data were presented as mean ± SEM. *n* = 6. ***P < 0.001. **G** Representative images of co-immunofluorescent staining of SYCP3 (red) and γH2AX (green) on spermatocyte spreads from P14 Control and *Mettl16* cKO mice are shown. The percent of each cell type was calculated in the lower panel. The DNA was stained with DAPI. Scale bars = 5 μm

Since no post-meiotic spermatozoa were present in the adult testes of *Mettl16* cKO mice, we next asked whether the meiosis is affected in *Mettl16* cKO mice. To this end, we analyzed meiotic hallmarks by immunostaining meiotic marker proteins in P10 testes of control and *Mettl16* cKO mice. The results showed that germ cells expressing both PLZF and STRA8 (PLZF^+^STRA8^+^) were present in control and *Mettl16* cKO mice; however, PLZF^−^STRA8^+^ cells, representing preleptotene spermatocytes, were rarely observed in *Mettl16* cKO mice (Fig. 3A-C). In addition, we found that phosphorylated H2AX (γH2AX)^+^ or synaptonemal complex protein 3 (SYCP3)^+^ cells were remarkably reduced in *Mettl16* cKO P10 testes compared with controls, especially PLZF^−^STRA8^+^γH2AX^+^ or PLZF^−^STRA8^+^SYCP3^+^ cells corresponding to pre-leptotene spermatocytes (Fig. 3D-E), which suggests the hallmarks of meiosis prophase I, including DSB formation and synapsis, were severely defective. In the control P10 testes, DNA was replicated (5-ethynyl-2′-deoxyuridine [EdU]^+^) preceding meiotic prophase I in pre-leptotene spermatocytes (PLZF^−^STRA8^+^EdU^+^) (Fig. 3C and F, *white arrows*). However, EdU signals were barely detectable in PLZF^−^STRA8^+^ germ cells in *Mettl16* cKO P10 testes, indicating that DNA replication was abnormal during S phase in the mitosis-to-meiosis transition in *Mettl16* cKO testes, which is akin to the METT-10 knock-out phenotype described in *C. elegans* [24]. To further investigate the defects in DSB and homologous chromosome synapsis in *Mettl16* cKO spermatocytes, we performed chromosome surface spreading assays by staining for SYCP3 and γH2AX in P14 control and *Mettl16* cKO mice. In control mice, three types of spermatocytes were observed in the testis, accounting for leptotene (38%), zygotene (20%) and pachytene (42%) (Fig. 3G). However, in *Mettl16* cKO testes, most cells were SYCP3^−^γH2AX^+^, and only 1% positive pre-leptotene spermatocyte-like cells harboring patchy aggregates of SYCP3 were found (Fig. 3G). These data suggest that METTL16 is required for the initiation of meiosis during spermatogenesis.

### METTL16 deletion alters expression of meiosis-related genes

**Fig.4:**
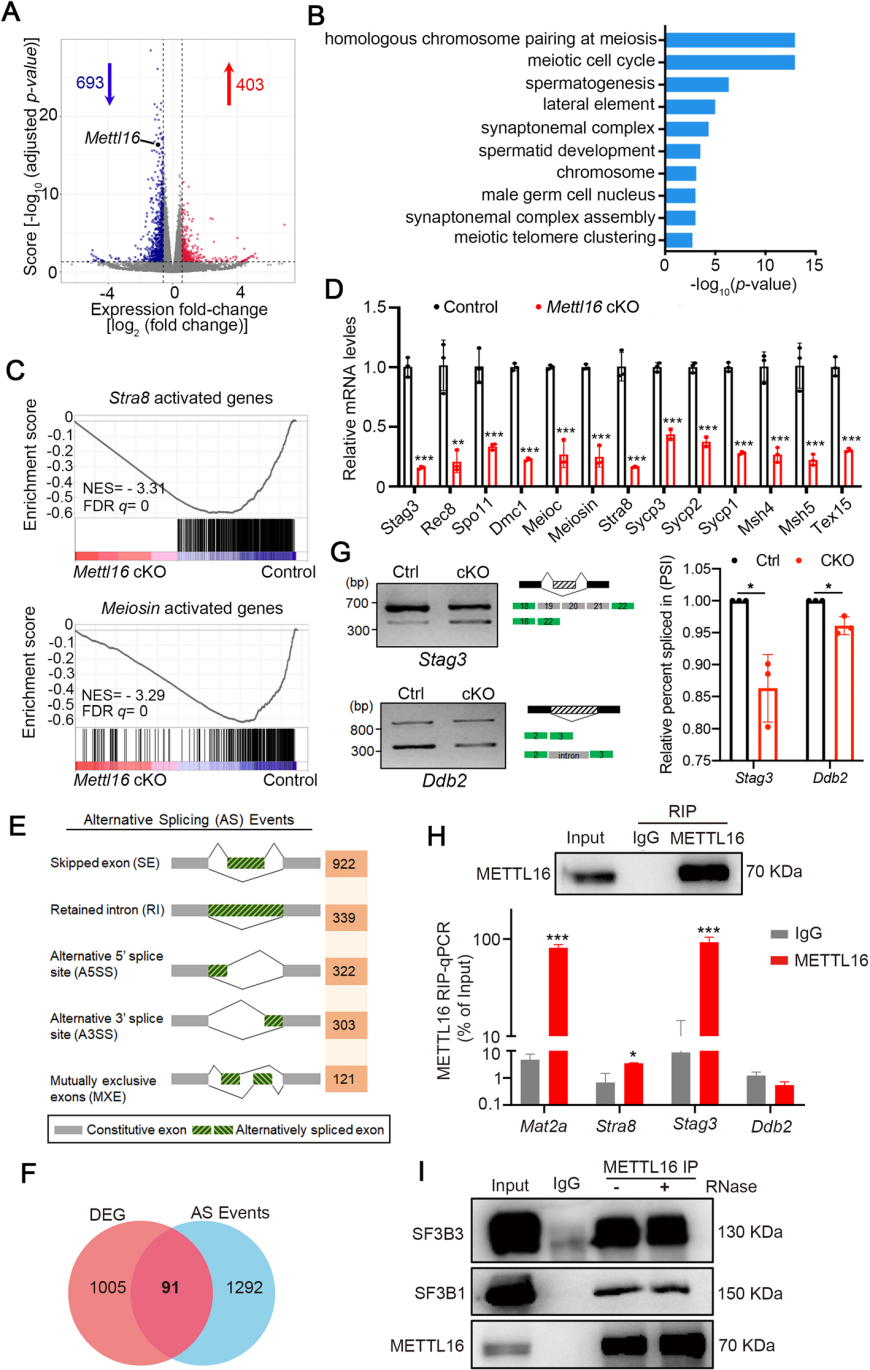
*Mettl16* cKO mouse testes display abnormal gene expression profiles and alternative splicing. **A** The volcano plot shows differentially expressed genes (DEGs) identified from RNA-seq from P10 *Mettl16* cKO and control testes. |log2FC|> 0.7 and an adjusted *P*-value < 0.05 were considered significant. Total identified 403 genes upregulated and 693 genes downregulated in *Mettl16* cKO testes. **B** GO term enrichment analysis of DEGs shows the top 10 enriched biological processes. **C** GSEA analyses of the RNA-seq data from P10 control and *Mettl16* cKO testes are shown. FDR q < 25%. NES, normalized enriched score. **D** RT-qPCR analysis verifies the indicated downregulated meiosis-related genes from RNA-seq data in c-KIT^+^ cells purified from P10 testes. Data are presented as mean ± SEM, *n* = 3. **P < 0.01, ***P < 0.001. *Actin* was used as an internal control for gene expression normalization. **E** Summary of differential alternative splicing (AS) events in testes from P10 Control and *Mettl16* cKO mice. The numbers of predicted AS events in each category are indicated. **F** Venn diagrams show the overlap between DEGs and AS events in *Mettl16* cKO versus control testes. **G** RT-PCR analysis for indicated genes (*Stag3* and *Ddb2*) in c-KIT-positive spermatogonia isolated from Control and *Mettl16* cKO mice at P10. The middle panels represent the schematic diagram of indicated AS exons. Right panel shows the quantification of percent spliced in (PSI). Data are presented as mean ± SEM, *n* = 3. *P < 0.05. **H** RIP-qPCR analysis of METTL16 binding genes as indicated. IgG was used as a control versus METTL16 antibody. RIP-qPCR enrichment was calculated concerning the Input. *Mat2a* was used as a positive control of selected METTL16 targets. **I** Immunoprecipitation (IP) analysis of METTL16 binding proteins (SF3B3 and SF3B1) in the presence or absence of RNase A is shown

To explore the molecular basis of how METTL16 regulates spermatogenesis, we compared the transcriptomes of control and *Mettl16* cKO mice at P10 testes, when spermatogonial differentiation and meiosis initiation has been completed, using high-throughput RNA-seq. Data analysis of three biological replicates yielded 403 upregulated and 693 downregulated genes (Fig. 4A, Additional file 2: Tables S1), mainly enriched in the meiotic cell cycle and homologous chromosome pairing during meiosis and synapsis (Fig. 4B, Additional file 3: Tables S2). Specifically, many down-regulated genes were involved in DNA replication, DSB repair, the cell cycle, and sister chromatid cohesion (Additional file 1: Fig. S3A, Additional file 4: Tables S3). Consistent with the defects in meiosis initiation, gene set enrichment analysis (GSEA) revealed that METTL16 deficiency dramatically decreased the enrichment of *STRA8* target genes and *MEIOSIN*-activated genes (Fig. 4C). Validation using reverse transcription-quantitative polymerase chain reaction (RT-qPCR) further confirmed the significant reduction in meiotic genes involved in chromosome axis formation (*Stag3*, and *Rec8*), DSB formation (*Spo11*, *Msh4*, *Msh5*, and *Dmc1*), and synapsis (*Sycp3*, *Sycp1*, and *Sycp2*) in P10 *Mettl16* cKO mice compared with that in control mice (Additional file 1: Fig. S3B). In addition, to eliminate the potential bias caused by different germ cell composition in control and *Mettl16* cKO testis, c-KIT^+^ spermatogonia were also purified from P10 testes, and used to validate the expression of these meiotic genes, which showed similar results to those in testes (Fig. 4D). These results suggest that METTL16 deficiency affects the expression of meiosis-related genes, thereby leading to defective meiosis during spermatogenesis.

### METTL16 modulates the alternative splicing of *Stag3* and *Ddb2*

Since previous studies reported that METTL16 could regulate the alternative splicing (AS) of SAMs in *Caenorhabditis elegans* [16] and human cell lines[13], we investigated whether AS was affected by METTL16 depletion in the mouse testes. We analyzed differential splicing events in the transcriptomes of control and *Mettl16* cKO testes using replicate multivariate analysis of transcript splicing (rMATS). Compared with the control, a large number of differential splicing events were identified in the testes of P10 *Mettl16* cKO mice, including skipped exon (SE), retained intron (RI), alternative 5’ splice site (A5SS), alternative 3’ splice site (A3SS), and mutually exclusive exons (MXE), with SE being the predominate differential splicing event (Fig. 4E, Additional file 1: Fig. S3C). Meanwhile, 55.6% of the differential AS events were upregulated in *Mettl16* cKO mice compared with control mice (Additional file 1: Fig. S3D), suggesting that METTL16 may be more likely to repress AS than promote AS in the testes. In addition, we found 91 genes that overlapped between differentially expressed genes (DEGs) and AS events, and gene ontology (GO) analysis showed that these genes were enriched in DNA recombination, homologous recombination, meiotic nuclear division, sister chromatid segregation, and meiotic cell cycle (Fig. 4F, Additional file 1: Fig. S3E). From the 91 overlapping AS genes, three meiotic-related genes, *Stag3* [25] (engaged in the formation of lateral element), *Stra8* [26] (a key regulator of meiosis initiation) and *Ddb2* [27] (DNA repair and germ cell apoptosis related), were selected to validate the reliability of RNA-seq data using RT-PCR on purified c-KIT-positive spermatogonia from P10 control and *Mettl16* cKO testes. Compared to controls, increased skipping of exons19-21 in *Stag3* mRNA and increased retention of intron 2 in *Ddb2* mRNA were observed in *Mettl16* cKO c-KIT-positive spermatogonia (Fig. 4G). However, despite the decreased skipping of exon3 in *Stra8* mRNA observed in *Mettl16* cKO c-KIT-positive spermatogonia, there were no statistically significant differences (Additional file 1: Fig. S3F-H). These data suggest that METTL16 may be involved in modulating the AS of mRNA of meiosis-related genes, such as *Stag3* and *Ddb2* to regulate their transcriptional levels.

To further explore whether the *Stag3, Ddb2,* and *Stra8* transcripts were the direct target of METTL16, RNA immunoprecipitation (RIP) combined qPCR (RIP-qPCR) analysis was performed using spermatogenic cells isolated from P10 control testes. These results confirmed that METTL16 binds directly to *Stra8* and *Stag3* mRNA but not to *Ddb2*, as shown by RIP-qPCR (Fig. 4H). In addition, we performed immunoprecipitation (IP) using the METTL16 antibody and found that METTL16 could interact with known splicing factors, including SF3B3 and SF3B1, and that their interaction was RNA-independent, as the addition of RNase did not affect their binding (Fig. 4I). Taken together, these results indicate that METTL16 may be involved in meiosis initiation by directly regulating the *Stag3* alternative splicing, probably by cooperating with splicing factors, such as SF3B3 and SF3B1.

### METTL16 regulates the translation efficiency of genes in the testes

We employed ribosome profiling (Ribo-seq) of P10 testes from control and *Mettl16* cKO mice to assess the effects of METTL16 deletion on mRNA translation. Translation efficiency refers to the ratio of normalized ribosome-protected fragments (RPFs) to mRNA fragments. Combined analysis of their transcriptome and translatome between the control and *Mettl16* cKO mice identified 1299 differentially transcribed genes (DTGs; 785 upregulated and 514 downregulated) and 1567 differential translation efficiency genes (DTEGs; 537 upregulated and 1030 downregulated) in *Mettl16* cKO testes (Additional file 5: Tables S4). Among them, 921 genes were both DTGs and DTEGs, 378 genes were exclusively DTGs, and 646 genes were exclusively DTEGs (Additional file 1: Fig. S4A). In addition, to preliminarily investigate the relationship between altered AS and translational regulation in *Mettl16* cKO mouse testes, we conducted an overlap analysis of AS events with DTEGs, and the results revealed that 85 genes underwent alternative splicing and translational regulation (Additional file 1: Fig. S4B). Further analysis of these 85 genes showed that they mainly correlated with cytoplasmic ribosomal proteins, ion homeostasis, and other basic biological processes not associated with phenotype-related terms (Additional file 1: Fig. S4C). Functional analysis revealed that the downregulated DTGs were enriched for meiotic cell cycle and male gamete formation, whereas no enrichment for meiosis- or spermatogenesis-related terms was found among the upregulated DTGs (Additional file 1: Fig. S4D). Conversely, terms related to meiotic nuclear division and cell cycle transition were enriched among the upregulated DTEGs, whereas those enriched among the downregulated DTEGs were unrelated to meiotic progression (Additional file 1: Fig. S4E). Specifically, many meiotic genes, such as *Stra8* and *Meioc*, were downregulated with increasing RPF abundance (Additional file 1: Fig. S4F). These opposite change patterns revealed by the transcriptome and translation profiles suggest a type of translational balance in the testes to compensate for the disruption of the transcriptional landscape in the absence of METTL16.

**Fig.5:**
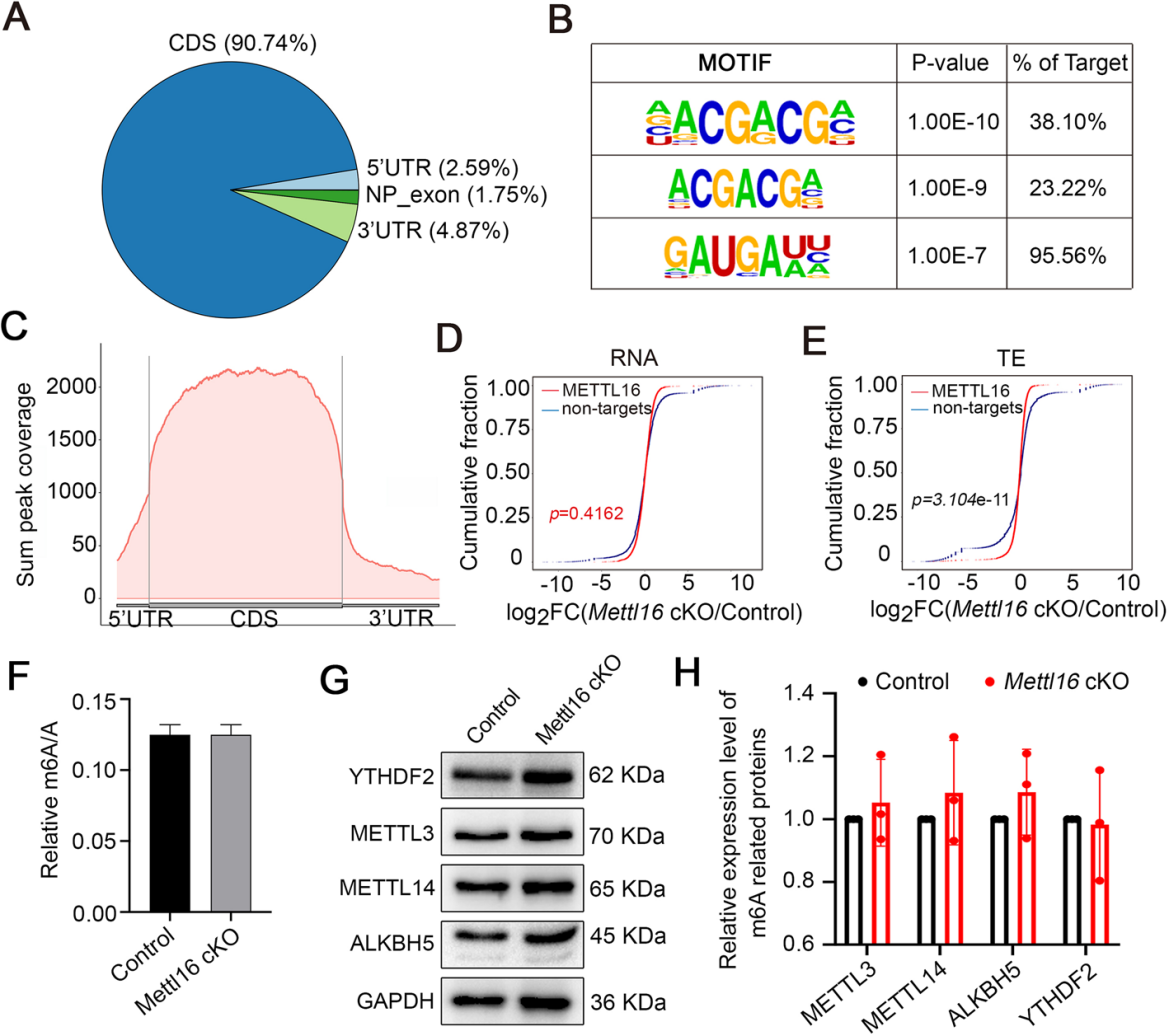
METTL16 decreases the mRNA abundance and improves the translation efficiency of its targets. **A** Pie chart depicting the binding peaks of METTL16 from the RIP-seq data. **B** The top 3 METTL16 binding peaks were identified through motif analysis based on RIP-seq data. **C** The distribution of METTL16 RIP peaks along the length of mRNA transcripts is shown. CDS indicates the coding sequence. **D**-**E** Cumulative distribution of RNA abundance (**D**) and translation efficiency (**E**) changes between Control (Ctrl) and *Mettl16* cKO (cKO) testes. RNA abundance was presented as the log_2_Fold Change(cKO/Ctl) in the X-axis. The blue curves indicate non-targets of METTL16, and the red curves indicate METTL16-RIP. *P*-values were calculated using a two-sided Wilcoxon test. **F** The m6A levels of testes from Control and *Mettl16* CKO mice at P8 are analyzed by LC-ESI-MS/MS. **G** Western blot analysis shows the expression levels of other m6A pathway proteins (METTL3, METTL14, YTHDF2, and ALKBH5) in testes from control and *Mettl16* cKO testis at P8. GAPDH was used as a loading control. **H** Histogram shows the quantification of the protein expression in (**G**). Data were presented as mean ± SEM, *n* = 3

To further clarify the role of METTL16 in the regulation of transcription and translation, we performed RIP-sequencing (RIP-seq) using P10 spermatogenic cells to analyze the mRNAs directly bound by METTL16. A total of 8554 peaks from 3465 genes were identified, and *Stra8* and *Stag3* were also present in the RIP-seq datasets (Additional file 6: Tables S5), consistent with the RIP-qPCR data (Fig. 4H). Further peak distribution and motif analyses revealed that METTL16 was preferentially bound to the coding sequence regions of mRNA, and the sequence “AGCG” was the top enriched motif (Fig. 5A-C). To explore the differences between METTL16 target and non-target genes at the transcriptional or translational level in the testes of *Mettl16* cKO mice, we analyzed the RIP-seq datasets in combination with RNA-seq or Ribo-seq datasets, respectively. Notably, cumulative distribution analyses revealed that in METTL16-deficient testes, METTL16 targets showed lower RNA abundance with METTL16 non-targets, and the translation efficiency of METTL16 targets was lower than that of non-targets (Fig. 5D-E). Considering the translation efficiency of several METTL16 targets, such as *Stra8*, were elevated, which prompted us to further divide the genes into two groups with Log_2_FC (cKO/Ctrl) > 0 and Log_2_FC (cKO/Ctrl) < 0” for further analysis. Interestingly, for the “Log_2_FC (cKO/Ctrl) < 0” group, METTL16 targets showed higher RNA abundance with METTL16 non-targets, and the translation efficiency of METTL16 targets was higher than non-targets in METTL16-deficient testes (Additional file 1: Fig. S4G-H). For the “Log_2_FC (cKO/Ctrl) > 0″ group, METTL16 targets showed lower RNA abundance with METTL16 non-targets, and the translation efficiency of METTL16 targets was also lower than non-targets in METTL16-deficient testes (Additional file 1: Fig. S4I-J). Collectively, these results indicate that METTL16 regulates gene transcription and translation in the mouse testes by direct targeting.

### The m6A transferase activity is essential for the function of METTL16 during spermatogenesis

**Fig.6:**
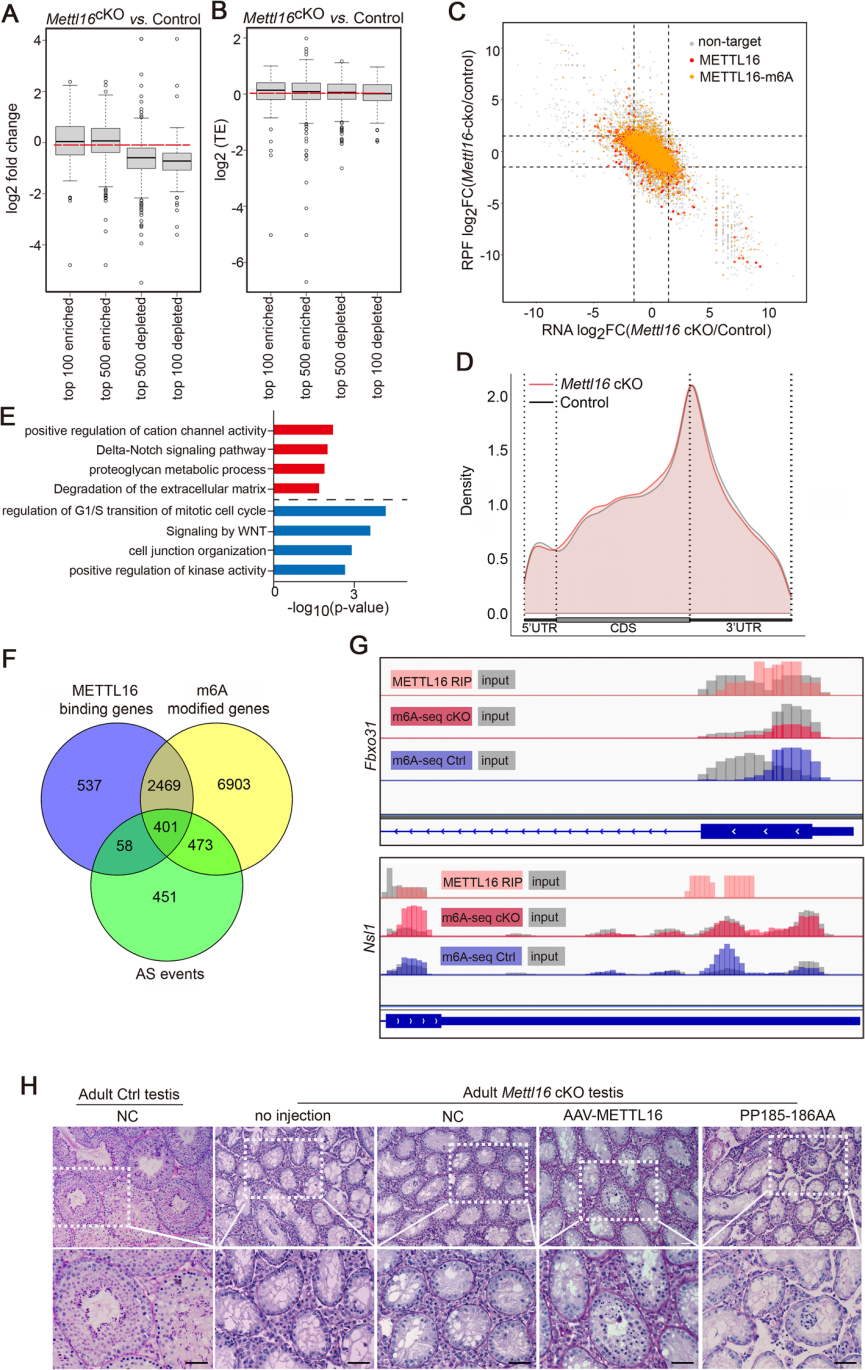
The catalytic activity of METTL16 is essential for spermatogenesis in mice. **A**, **B** Combined analysis of transcripts harboring differential m6A levels (Top 100 or 500 genes that are m6A-enriched or m6A-depleted in m6A-IP libraries) with DEGs (*Mettl16* cKO *v.s.* Control) from RNA-seq (**A**) or genes with differential TE (**B**). The m6A-enriched or m6A-depleted genes were identified based on *P*-values in P8 testis according to previously reported m6A-IP data (PMID: 29033321). **C** Scatter plots show the combined analysis of RIP-seq and m6A-seq from Control and *Mettl16* cKO testes. Gray dots (non-target) indicate genes with no METTL16-binding sites. Red dots indicate genes with METTL16-binding sites. Orange dots indicate genes with overlapped METTL16-binding and m6A-modified sites. **D** Distribution of m6A-modified peaks of Control and *Mettl16* cKO along the length of mRNA transcripts. **E** GO term enrichment analyses of genes with m6A-modification show the top 4 terms enriched in genes with down-regulated (blue) and up-regulated (red) m6A-modified peaks. **F** Venn diagrams show the overlap among AS genes (*Mettl16* cKO vs Control in RNA-seq), METTL16 binding genes (RIP-seq), and m6A modified genes (wild-type in m6A-seq). **G** Integrative Genomics Viewer (IGV) shows the distribution of m6A-modified peaks and METTL16-binding peaks along with indicated transcripts (*Fbxo31* and *Nsl1*) in METTL16 RIP-seq and m6A-seq data. **H** Histological analyses of testes from *Mettl16* cKO adult mice injected into AAV9 vectors carrying wild-type or mutant (catalytic dead) METTL16 are shown. No injection or NC (empty vector) injection was used as the negative control. The histology was analyzed by PAS two weeks after injection. Scale bars = 50 μm

Given recent reports have shown that it can deposit m6A into hundreds of its specific messenger RNA targets [18], we used liquid chromatography-electrospray ionization tandem mass spectrometry (LC-ESI-MS/MS) to preliminarily investigate whether the m6A transferase activity of METTL16 is required for spermatogenesis. To this end, we analyzed global m6A levels in total RNA (including rRNA, ncRNA, and mRNA) of testicular cells from control and *Mettl16* cKO mice at P8 by LC-ESI-MS/MS (Additional file 7: Table S6). Interestingly, no significant changes in the overall m6A levels were observed in METTL16-deficient and control testes (Fig. 5F), possibly due to the influence of a high proportion of rRNA and the inclusion of Sertoli cells in the *Mettl16* cKO testes. We next asked if other components of the m6A pathway are affected upon METTL16 depleted in the testes. We thus investigated the expression of m6A writers (METTL3, METTL14), reader YTHDF2, and eraser ALKBH5 in P8 testes by Western blot. Interestingly, the results showed that METTL16 deletion does not affect the protein levels of these classical m6A pathway proteins (Fig. 5G-H), suggesting a direct role of METTL16 in regulating the m6A levels during spermatogenesis. Interestingly, when we combined the analysis of DEGs with m6A-enriched or depleted transcripts obtained from P8 testes m6A-IP libraries [28], we found that the m6A-enriched transcripts (the top 100 or 500 genes enriched in the m6A-IP libraries) were upregulated in the *Mettl16* cKO testes compared to the controls. In contrast, the m6A-depleted transcripts were generally downregulated in *Mettl16* cKO testes (Fig. 6A). However, translation efficiency was comparable between the m6A-enriched and m6A-depleted transcripts (Fig. 6B). These bioinformatic analyses revealed that the m6A-enriched transcripts might not be directly mediated by METTL16, but the ablation of METTL16 could cause the differential expression between the m6A-enriched and m6A-depleted transcripts, and METTL16 might regulate mRNA TE with no m6A bias in testicular cells.

To examine whether METTL16 deletion influences the m6A modification of transcripts in the testes, we performed m6A-seq to determine the changes in m6A peaks in P10 testes from control and *Mettl16* cKO mice. A total of 19689 and 19367 m6A peaks were identified in control and *Mettl16* cKO testes, respectively (Additional file 8: Tables S7). Notably, the combined analyses of METTL16 RIP-seq from purified P10 spermatogenic cells and m6A-seq data revealed that 2870 (82.8%) METTL16-binding targets carried m6A modifications (Fig. 6C). The m6A peaks identified in both control and *Mettl16* cKO testes were mainly enriched in the 3’ untranslated region (UTR) and were closer to the transcription termination site (TTS) (Fig. 6D). Compared to controls, 196 downregulated m6A peaks and 106 upregulated m6A peaks were identified in *Mettl16* cKO testes (Additional file 9: Tables S8), suggesting that METTL16 depletion in the testes could affect the m6A modification of transcripts. Further functional gene analyses revealed that downregulated m6A peaks were highly enriched in the regulation of G1/S mitotic cell cycle transition (Fig. 6E). In addition, combined bioinformatic analysis revealed that 459 genes with altered AS events correlated with METTL16 binding and 874 AS events changed genes associated with m6A peaks (Fig. 6F). Surprisingly, no m6A modification sites were identified on *stag3* and *Stra8* in the m6A-seq. Notably, among the downregulated m6A peaks, cell cycle-related genes *Fbxo31* and *Nsl1* were identified to be bound by METTL16 (Fig. 6G), although the mRNA levels of these two genes remain unchanged.

To further test the physiological function of m6A transferase activity in METLL16 during spermatogenesis, we performed a rescue assay by injecting adeno-associated virus 9 (AAV9) vectors carrying wild-type METTL16 or catalytically dead mutant METTL16 (PP185-186AA, inactivated the m6A transferase activity) into the adult testes of *Mettl16* cKO mice (Additional file 1: Fig. S5A). Compared with negative controls, AAV9 vectors expressing wild-type METTL16 could partially rescue the defective spermatogenesis of *Mettl16* cKO mice, as pachytene spermatocytes were observed in some seminiferous tubules (Fig. 6H, *the fourth panel from left*). Injection of AAV vectors carrying the catalytically dead mutant METTL16 did not affect spermatogenesis in *Mettl16* cKO mice (Fig. 6H, *the fifth panel from left*). Altogether, these data suggest that METTL16 may play a critical role in spermatogenesis by regulating m6A levels of METTL16 bound genes involved in male germ cell development.

### METTL16 interacts with the MEIOC/YTHDC2/RBM46 complex

**Fig.7:**
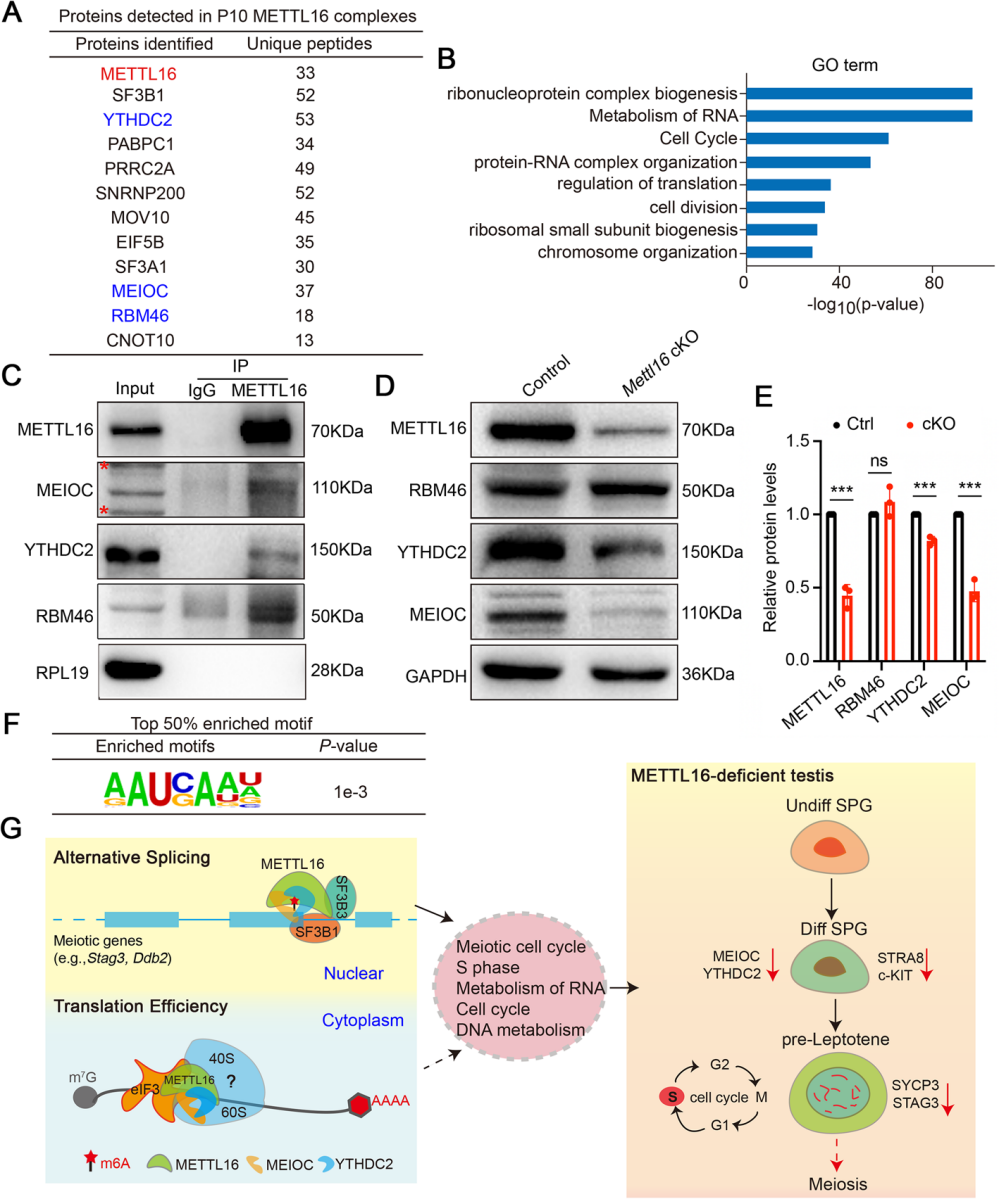
METTL16 is a binding partner of MEIOC/YTHDC2/RBM46 complex in mouse testes. **A** A list of eleven METTL16-interacting partners in P10 mouse testes identified by IP-MS is shown. **B** Go term enrichment analyses showing the METTL16-interacting proteins identified from IP-MS data. **C** Validation of interactions between METTL16 and three putative METTL16-interacting proteins (MEIOC, YTHDC2, and RBM46) in P10 mouse testes by IP assays are shown. Mouse testis lysates were subjected to IP with anti-METTL16 or IgG control antibodies. Asterisk indicates non-specific bands. **D** Western blot analyses show the expression levels of METTL16-interacting proteins (MEIOC, YTHDC2, and RBM46) in testes from control and *Mettl16* cKO testis at P10. GAPDH was used as a loading control. **E** Histogram shows the quantification of the protein expression in (**D**). Data were presented as mean ± SEM, *n* = 3. ns, not significant. ***P < 0.001. **F** The motif analysis shows that the top 50% METTL16 binding sites are ‘AAUCAA’, similar to the YTHDC2 and RBM46 binding sites. **G** A schematic illustration of the function of METTL16 during spermatogenesis is shown. METTL16 interacts with alternative splicing factors (e.g., SF3B1 and SF3B3) to regulate AS of meiotic genes for governing the meiotic gene expression program in the nuclear of male germ cells. Meanwhile, METTL16 balance translation efficiency for proper cell cycle and a successful meiosis initiation in the cytoplasm of male germ cells

To better understand the mechanism of METTL16-mediated regulation of mRNA metabolism and translation in the testes, we performed immunoprecipitation-coupled mass spectrometry (IP-MS) using the METTL16 antibody in P10 testes to unbiasedly identify the interactomes of METTL16. Consequently, thousands of proteins were identified in the METTL16 antibody pull-down products (Additional file 10: Tables S9). Notably, the splicing factor SF3B1 was found in METTL16-interacting proteins with highly unique peptides (Fig. 7A), further confirming the potential function of METTL16 in regulating AS by interacting with splicing factors. GO analysis of the candidate interacting proteins showed enrichment of functional terms related to RNA metabolism, cell division, and chromosome organization (Fig. 7B). Importantly, MEIOC, YTHDC2, and RBM46 were identified from METTL16 interactomes as highly unique peptides and were verified to bind to METTL16 using co-immunoprecipitation (Co-IP) (Fig. 7C, Additional file 1: Fig. S5B). Since MEIOC, YTHDC2, and RBM46 have been reported to form a complex and responsible for down-regulating mitotic transcripts during meiosis entry in mammalian spermatogenesis [28–30], we further examined the protein expression levels of MEIOC, YTHDC2, and RBM46 in P10 testes. We found that, compared to control mice, MEIOC and YTHDC2 were significantly downregulated in *Mettl16* cKO mice, whereas the expression level of RBM46 was comparable between control and *Mettl16* cKO mice (Fig. 7D-E). In addition, we found ‘AAUCAA’ were also identified among the top 50% of METTL16 binding peaks (Fig. 7F), which was similar to YTHDC2 and RBM46 binding sites [29]. These results suggest that METTL16 may interact with the MEIOC/YTHDC2/RBM46 complex and potentially co-regulate its target genes during meiotic initiation (Fig. 7G).

## Discussion

As revealed by the high-resolution map of m6A modifications in mouse germ cells, the distribution of m6A sites was dynamically regulated during spermatogenesis. However, the mechanisms through which m6A writers control these processes remain largely unknown. Previous research has shown that germ cell-specific deletion of m6A writers, either *Mettl3* or *Mettl14*, using *Vasa-Cre* leads to a depletion of SSCs, whereas germ cell-specific inactivation of either *Mettl3* or *Mettl14* using *Stra8-Cre* does not affect spermatogenesis. Interestingly, simultaneous knockout of *Mettl3* and *Mettl14* via *Stra8-Cre* causes defective spermiogenesis [11], suggesting that m6A modifications may function at different stages of male germ cell development. Furthermore, the high enrichment of m6A in meiosis-associated transcripts and the dynamic changes in methylated sites on these transcripts at different developmental stages of meiotic cells support the idea that m6A modification may regulate mouse meiosis, raising the question that in addition to *Mettl3* and *Mettl14*, whether more methyltransferases contribute to the m6A deposition on transcripts during mammalian meiosis [11]. Although *Vasa-Cre*-mediated *Mettl16* knockout (KO) mice have been reported to be infertile [16], the *Stra8-Cre*-induced KO mouse model was more suitable than the *Vasa-Cre*-mediated KO mouse model for investigating the gene function in spermatogonial differentiation and meiosis. In addition, as METTL16 was identified as an m6A writer essential for mouse embryonic development [8, 15], we investigated its role in postnatal male germ cell development by using the *Stra8*-*Cre-*induced conditional KO strategy. As expected, we found that *Mettl16* is required for spermatogonial differentiation and meiosis initiation, confirming that other m6A writers are involved in this process.

It is worth noting that in spermatids from *Mettl3/Mettl14* double-knockout mice, the expression of genes essential for spermiogenesis is transcriptionally unchanged but translationally inhibited [11]. However, in *Mettl16* cKO mice, genes critical for meiotic initiation were transcriptionally inhibited and translationally accelerated. One explanation for this paradox is that the elevated TE of these essential meiotic genes may be due to efforts made by germ cells to compensate for the sharply decreased mRNA under stress. Although TE was upregulated to accelerate the synthesis of meiotic proteins, the mRNA concentration (predicted to regulate ribosome occupancy and density) was so low that the protein expression could hardly be rescued [31], resulting in defects in meiotic initiation. To the best of our knowledge, this is the first study to reveal that METTL16 is involved in the regulation of meiotic gene expression, both transcriptionally and translationally.

In mouse testes, some SSCs can maintain the ability to proliferate and self-renew, whereas others differentiate into type A1, type B, and differentiated spermatogonia. In *Mettl16* cKO mice, the number of GFRα1-positive SSCs was normal, which may explain why DDX4-positive germ cells still remained in the adult testes. However, the number of c-KIT^+^ differentiating spermatogonia was significantly reduced, and PLZF^−^STRA8^+^ pre-leptotene spermatocytes undergoing DNA replication before entering meiotic prophase I were barely detected in *Mettl16* cKO testes, demonstrating that both spermatogonial differentiation and entry into meiosis were affected by METTL16 depletion in male germ cells. This is the first report in which the conditional knockout of one m6A writer (METTL16) in male germ cells driven by *Stra8*-Cre causes defective spermatogenesis. Interestingly, a much more profound defect in spermatogenesis was observed upon *Vasa-Cre* mediated *Mettl3* or *Mettl14* deletion, as SSCs were affected and germ cell development was arrested at the zygotene-like stage, exhibiting the Sertoli cell-only syndrome phenotype in adults [11]. Therefore, METTL16 displayed a distinct function in spermatogonial differentiation and meiosis initiation, different from that of other m6A writers (METTL3 and METTL14), adding another layer to the physiological roles of m6A modification mediated by m6A writers in spermatogenesis.

Furthermore, m6A levels were significantly reduced in the spermatids of *Mettl3/Mettl14*-double knockout mice [11]; however, the overall m6A level was not significantly altered in the testes of *Mettl16* cKO mice. This was likely because m6A modification is mainly installed by *Mettl3/Mettl14* in the RRm6ACH motif near the stop codon of thousands of mRNA transcripts [32], but METTL16 has been reported to only target *Mat2a*, *U6*, metastasis associated lung adenocarcinoma transcript (*Malat*), branched chain amino acid transaminase 1 (*Bcat1*)*,* and *Bcat2* [15, 33, 34]. In addition, the changes in the entire m6A levels were subtle due to the limited substrates, resulting in them hardly being detected using LC-MS. However, this does not exclude the possibility that the subtle m6A changes also exert profound effects on meiotic gene expression; the expression of m6A enriched transcripts was upregulated in *Mettl16* cKO mice at P10, which is consistent with that in *Ythdc2* knockout P8 testes and *Meioc* mutant P8 testes [28]. Notably, translation efficiency was altered with no m6A bias after METTL16 ablation, similar to previous reports that METTL16 regulates translation efficiency in an m6A-independent manner in human cell lines, facilitating the translation of thousands of transcripts by promoting the formation of translation initiation complexes [18]. Importantly, the combined RIP-seq and m6A-seq analyses of METTL16 revealed that the m6A peaks identified in both control and *Mettl16* cKO testes were mainly enriched in the 3’UTR region and were closer to the TTS, which was consistent with a previous report that m6A modifications were distributed near stop codons and in 3’UTR [7]. Among the downregulated m6A peaks in *Mettl16* cKO testes, *Fbxo31* and *Nsl1* were identified to be bound by METTL16. Given that *Fbxo31* plays a central role in G1 arrest following DNA damage [35] and *Nsl1* is required for normal chromosome alignment and segregation during mitosis [36, 37], the alterations of m6A modification in these cell cycle-related genes may explain the pre-leptotene arrest in *Mettl16* cKO testes. Recent studies have shown that METTL16 binds directly to eIF3A/B or eIF4E to promote translation [38]. However, no eukaryotic translational initiation factors with significant fold changes were found in the METTL16 antibody-immunoprecipitated fraction, nor was the eIF3B band observed when Co-IP analysis was applied to the mouse testes. Thus, METTL16 may participate in translation control, either indirectly or in an unknown manner.

In *C. elegans* and human cells, METT-10 (an ortholog of mouse METTL16) deposits m6A on the 3’ splice site of *SAM* pre-mRNA and regulates proper splicing of SAM synthetase *Mat2a* via either its methyltransferase or non-methyltransferase activities [15, 16]. In the current study, although *Mat2a* was identified in the A3SS events and directly bound by METTL16 according to RIP-qPCR data, further analysis suggested that the expression level of *Mat2a* was not altered in *Mettl16* cKO testis and the splice site of *Mat2a* in *Mettl16* cKO mice was different from that in humans (Additional file 1: Fig. S5C). Therefore, the spermatogenesis arrest in *Mettl16* cKO mice may not be directly correlated with the A3SS splicing of *Mat2a* in mice. In this study, we found 91 overlapping DEGs and AS genes in P10 *Mettl16* cKO testes that were mainly enriched in DNA recombination, sister chromosomes segregation, and the meiotic cell cycle, confirming that METTL16 functions in mouse meiotic progression by regulating AS. Interestingly, we discovered that METTL16 could bind to known splicing factors SF3B1 and SF3B3 [39, 40], as well as the mRNA of *Stag3* and *Ddb2*. METTL16 knockout caused increased exon skipping in *Stag3* and increased intron retention in *Ddb2*. Previous studies have shown that *Stag3* is a key meiosis-specific STAG protein that associates with the cohesin proteins SYCP2 and SYCP3 to form an axial element (AE) in early meiotic prophase I [25]. Moreover, in the meiocytes of *Stag3* knockout mice, no AE was formed and synapsis did not occur; only diffused dot-like or aggregate SYCP3-positive signals were observed in leptonema-like cells [41]. Similar to *Stag3*-deficient mice, only low-level, diffused, weak SYCP3 signals were found in the meiotic spermatocytes of our *Mettl16* cKO mice, suggesting that no AE was formed in *Mettl16* cKO mice. Therefore, our study is the first to demonstrate that dysregulated AS of key regulators targeted by METTL16 may be a reason why the mitosis-to-meiosis transition is compromised upon METTL16 ablation in mice, suggesting that METTL16-mediated AS is essential for male meiotic progression. However, it remains to be elucidated whether the m6A modifications around the AS sites in *Stag3* mRNAs also change upon METTL16 deletion. This opens up renewed interest in studying the role of m6A writers in male meiosis and germ cell development. Moreover, we also designed minigenes of *Stag3* and *Ddb2* (cloning splicing regulated region into pcDNA3.1) and co-transfected them with wild-type METTL16 or catalytically dead mutant METTL16 into HEK293T cells in vitro, respectively. Surprisingly, the splicing pattern of these two genes in vitro was found to be different from that in spermatogonia. The primary transcript of *Ddb2* was different, and *Stag3* did not even undergo alternative splicing in HEK293T cells, although wild-type METTL16 and the catalytically dead mutant FLAG-METTL16 were normally expressed (Additional file 1: Fig. S5D-E). Thus, this in vitro method may not always be suitable for all gene AS studies, and AAV injection into neonatal mouse testis through efferent duct injection may be a better way to study gene AS in spermatogonia in the future.

METTL16 may play a critical role in AS regulation through two main axes: the METTL16/U6 axis and the METTL16/SF3B axis. METTL16 could methylate U6 snRNA and likely influence interactions with other splicing factors, thereby affecting spliceosome assembly and splice site selection, ultimately modulating alternative splicing patterns of specific genes. In addition, METTL16 directly interacts with SF3B1/ SF3B3 and these interactions may regulate the functional states of SF3B proteins, affecting their assembly into the spliceosome and their role in AS. Therefore, the METTL16/U6 axis and the METTL16/SF3B axis may contribute to the regulation of spliceosome function and alternative splicing patterns, which are critical for maintaining gene expression in spermatogenesis.

In the current study, the potential difference in germ cell composition between the wild-type control and METTL16 cKO groups at P10 likely introduced a bias in the detected differentially expressed genes (DEGs) identified by RNA-seq. We hypothesized that these DEGs may be influenced by indirect factors rather than direct regulation by METTL16. Nevertheless, by choosing the P10 time point for RNA-seq, we were able to obtain a large number of DEGs in Mettl16 cKO mice. Our RNA-seq results revealed 403 up-regulated and 693 down-regulated genes with absolute log2-transformed fold changes greater than 1.5 and an adjusted *P*-value < 0.05, and this number of DEGs is acceptable in the context of our study. Despite this bias, we performed RNA-seq at P10 to study spermatogonial differentiation and the onset of meiosis simultaneously. To overcome this challenge, we strategically isolated cKit + spermatogonia and performed qPCR for validation, which increased the relevance of our findings to spermatogonial differentiation and minimized potential noise from other testicular cell types.

## Conclusion

Based on the functions of the m6A writer METTL16, we propose a molecular model in which METTL16 regulates spermatogenic-related genes via two mechanisms, m6A transferase activity-dependent transcription regulation and m6A-independent mRNA TE regulation, to control proper spermatogonial differentiation and meiotic initiation. A new insight into METTL16 and MEIOC/YTHDC2/RBM46 interaction was revealed, forming a complex as the critical transcriptional and translational regulator in testes. The exact mechanism by which the m6A modification pathway is mediated by METTL16 requires further biochemical and genetic studies. Nevertheless, our data strongly support the hypothesis that METTL16-mediated AS and TE regulation are essential for spermatogonial differentiation and meiosis initiation. Our findings provide a starting point for further elucidation of the functional importance of m6A writers in the early stages of mammalian spermatogenesis.

## Methods

### Ethics statement

All mice were bred and housed under specific pathogen-free conditions with controlled temperature (22–25 °C) and 50–70% humidity in the animal center of Huazhong University of Science and Technology. All animal experiments were approved by the Institutional Animal Care and Use Committee of Huazhong University of Science and Technology and conducted in accordance with the Guide for the Care and Use of Laboratory Animal (approval number: S2795).

### Mice

*Mettl16*^*flox/*+^ mice were generated by Cyagen Biosciences company. The *Stra8-Cre* mouse line was gifted from Professor Minghan Tong of the Center for Excellence in Molecular Cell Science, Chinese Academy of Sciences (Shanghai, China). There are 10 exons in the mouse *Mettl16* gene, with the ATG start codon in exon 2 and the TAA stop codon in exon 10 (Transcript: ENSMUST00000141755). The loxP sites were inserted into the intron 2 and intron 3 of *Mettl16* gene to generate the *Mettl16*^+*/flox*^ mice. *Mettl16*^*flox/flox*^ mice were generated by mating *Mettl16*^*flox/*+^ and *Mettl16*^*flox/*+^ mice. *Mettl16*^*flox/*+^*; Stra8-Cre* mice were generated by breeding *Stra8-Cre* mice with *Mettl16*^*flox/*+^ mice. *Mettl16*^*flox/Del*^*; Stra8-Cre* (designated as *Mettl16* cKO) mice were generated by crossing male *Mettl16*^*flox/*+^*Stra8-Cre* mice with female *Mettl16*^*flox/flox*^ mice. Exon 2 of *the Mettl16* gene contains 128 bp that encodes protein. Deletion of exon 3 of the *Mettl16* gene will result in a frameshift and might produce a short peptide encoding about 43 amino acids, which is too small (~ 4.7 KDa) to be detected. The C-terminal EGFP-tagged METTL16 (*Mettl16*^EGFP^-Tagged) mice were generated by Cyagen Biosciences company. The sgRNA to the mouse *Mettl16* gene, the donor vector containing the ‘3xEAAAK-EGFP’ cassette, and Cas9 mRNA were co-injected into fertilized C57BL/6 J mouse oocytes to generate targeted knockin offspring. F0 founder mice were identified by PCR followed by Sanger sequence analysis and mated with wild-type C57BL/6 J mice to obtain the F1 generation. Genotyping of the mice was performed using PCR amplification of genomic DNA extracted from mouse tails. The primer sequences used are listed in Additional file 11: Table S10.

### Construction of expression plasmids and cell culture

Full-length METTL16, mutant METTL16 expression constructs were generated by PCR amplification using adult mouse testis cDNA, followed by purification with agarose gel electrophoresis. Then, PCR products were subcloned into the p3xFLAG-CMV plasmids to yield expression constructs before transfection into 293 T cells. For the generation of mini-gene of *Stag3* and *Ddb2*, splicing regions were generated by PCR amplification using mouse tail genome DNA and were subcloned into pcDNA3.1 plasmids, respectively. HEK293T cells were maintained in DMEM with 10% FBS and seeded into 24 well dishes. Expression plasmids were transfected in 293 T cells using Lipo2000 following the manufacturer’s instruction. Cells were collected 48 h after transfection, and RT-PCR was performed.

### Fertility test

Fertility was analyzed using caged 8-weeks-old wild-type female mice with control or *Mettl16* cKO male mice for at least 5 months. Generally, two females were mated with one male in each cage, and the females were checked for vaginal plugs every morning. Pregnant female mice were single-caged and the number of offspring was recorded.

### PAS staining

Mouse testes and epididymides were dissected and fixed in Bouin’s solution (HT10132, Sigma-Aldrich, USA) for 24 h at room temperature (RT), followed by five washes with 75% ethanol for 30 min. After dehydration, samples were embedded in paraffin and sectioned into 5 μm slices. Testicular and epididymal sections were dewaxed and sequentially hydrated, followed by incubation with 0.5% periodic acid solution at RT in the dark for 20 min. The slides were then washed twice with phosphate buffered saline (PBS) and incubated with Schiff’s reagent at RT for 15 min. Nuclei were counter-stained with hematoxylin, and the sections were dehydrated and sealed with neutral gum for photographing.

### Immunofluorescence staining

For immunofluorescence staining, mouse testes were fixed in 4% paraformaldehyde (PFA) for 4–6 h at RT and sequentially dehydrated with a sucrose gradient. After shaking in Tissue-Tek O.C.T compound (4583, Sakura Finetek, Torrance, CA, USA) for 1 h, the tissues were embedded using liquid nitrogen. Tissue sections were cut at 5 μm and mounted on slides. Antigen retrieval was performed by boiling slides in 0.01 M sodium citrate buffer at approximately 95 °C for 20 min in a microwave. Tissue sections were washed with PBS, blocked with 3% bovine serum albumin for 30 min at RT, and incubated with primary antibodies at 4 °C overnight in a humidified chamber. After washing three times with PBS, the sections were incubated with secondary antibodies for 1–2 h at RT. The slides were mounted with VectorShield mounting medium containing DAPI after rinsing three times with PBS. The images were captured using a confocal microscope (Zeiss, Germany). Detailed information on the antibodies used in this study is provided in Additional file 12: Table S11.

For the EdU incorporation analysis, control and *Mettl16* cKO male mice (P10) were injected with 50 mg/kg EdU (dissolved in PBS). After 2 h, the testes were removed, and cryosections were prepared as described above. EdU signals were detected using an EdU Cell Proliferation Image Kit (green fluorescence) (KTA2030, Abbkine, Wuhan, China) according to the manufacturer’s instructions.

### Chromosome spread analysis

Testes were dissected from P12 male mice (control and *Mettl16* cKO) and the tunica albuginea was removed. Testicular seminiferous tubules were treated with hypotonic buffer containing 30 mM Tris (pH 8.2), 17 mM trisodium citrate dihydrate, 50 mM sucrose, 5 mM ethylenediaminetetraacetic acid, 0.5 mM dithiothreitol, and 0.5 mM phenylmethylsulfonyl fluoride for 60 min. The tubules were cut into fragments and suspended in 100 mM sucrose on slides. After treatment with 2% PFA containing 0.15% Triton X-100, the slides were incubated in a humidified chamber for 4–6 h at RT and washed with 0.4% Photo-Flo (1,464,510, Kodak, Rochester, NY, USA). The slides were air-dried and immunostained for imaging using a Axio Scope A1 microscope (Zeiss).

### c-KIT-positive spermatogonia isolation

Spermatogonia were isolated from freshly harvested P10 control and *Mettl16* cKO mouse testes. Briefly, testes were digested with 1 mg/ml collagenase IV at 37 °C for 10 min, centrifuged, and washed with Dulbecco's Modified Eagle Medium. Seminiferous tubules were subsequently digested with 0.25% trypsin containing 1 mg/ml DNase I at 37 °C for 3 min to prepare single-cell suspensions. Cell clumps were removed using a 40 µm nylon mesh. The cells were incubated for 15 min at 4 °C with CD117 microbeads (Miltenyi Biotec, Germany) and magnetically separated using columns to obtain c-KIT-positive spermatogonia.

### RNA-seq and RT-qPCR

Total RNA was extracted from P10 mouse testes or c-KIT-positive spermatogonia using TRIzol reagent (Thermo Fisher Scientific, Waltham, MA, USA) according to the manufacturer’s instructions and subjected to high-throughput RNA-seq or RT-qPCR. For RNA-seq, three biological replicates were performed using P10 control and *Mettl16* cKO testes, with one mouse for each sample. Poly (A +)-enriched cDNA libraries were generated and raw data (base pairs) were produced using the Illumina Hi-Seq 2500 platform (Illumina, San Diego, CA, USA). Data were trimmed with Cutadapt v1.9.1 and mapped to the UCSC mm10 genome using HiSAT2 v2.0.1, with the default parameters. Statistical significance tests for DEGs were performed using DEseq2. DEGs were defined as genes with absolute log2-transformed fold changes greater than 1.5 and an adjusted *P*-value < 0.05. GSEA using GO and Kyoto Encyclopedia of Genes and Genomes were performed using the Database for Annotation, Visualization, and Integrated Discovery. rMATS was used to analyze the splicing events. Events with a false discovery rate < 0.05 and |△PSI|> 10% were regarded as differential splicing events. Gene track view of interested genes were visualized using the IGV browser.

For RT-qPCR, cDNA was synthesized using a PrimeScript RT Reagent Kit (Takara Bio Inc., Japan). Quantitative PCR was performed using SYBR Green Master Mix (Takara Bio Inc.) and analyzed using an LC480 (Roche Applied Science, Germany). The primer sequences are listed in Additional file 11: Table S10. The relative expression of 12 meiotic genes in P10 testes and purified c-KIT^+^ spermatogonia were detected to validate the RNA-seq results, with *Gapdh* and *Actin* used as internal controls for gene expression normalization, respectively. Additionally, *Stra8* expression in c-KIT^+^ spermatogonia was also tested. At least three replicates were performed. Statistical analysis between normalized gene expression in each sample was performed by two-sided Student’s *t*-test using GraphPad Prism 8.0.

### Western blot

Testis proteins were extracted using RIPA lysis buffer, then boiled with 5 × sodium dodecyl-sulfate (SDS) loading buffer and separated using SDS- polyacrylamide gel electrophoresis (PAGE). Proteins from the PAGE were transferred to polyvinylidene difluoride membranes, blocked with 5% skim milk in Tris-buffered saline with 0.1% Tween20 (TBST) for 1 h at RT, and incubated with primary antibodies at 4 °C overnight. After washing with TBST, the membranes were incubated with corresponding horseradish peroxidase-conjugated secondary antibodies for 1 h at RT. Images were captured using a Luminol/Enhancer and Peroxide solution (Clarity Western ECL Substrate, Bio-Rad, Hercules, CA, USA). Detailed information on the primary and secondary antibodies used is provided in Additional file 12: Table S11.

### RNA immunoprecipititation (RIP)-seq

RIP-seq was performed on spermatogenic cells from P10 testis as described previously [42]. Briefly, about forty testes from twenty P10 mice were dissected, decapsulated in PBS at RT, and then treated with collagenase and trypsin sequentially in a 37 °C water bath to obtain a single-cell suspension. Somatic cells, such as Sertoli cells, were removed in overnight culture, leaving spermatogenic cells. The cell pellets were collected, resuspended in PBS, cross-linked in the ultraviolet cross linker with a 150 mj/cm^2^, and lysed in RIP lysis buffer. After centrifugation at 4 °C, the supernatant was collected and 1/10 lysis buffer were kept for ‘Input’ sample. The remaining lysis buffer were incubated with pre-cleaned magnetic beads (MCE) with 5 µg METTL16 antibody or IgG (used as control) at 4 °C overnight. The beads were washed three times, and protein-bound mRNAs were extracted using TRIzol and chloroform. The purity and integrity of the extracted mRNAs were assessed using an Agilent Bioanalyzer (Agilent Technologies, Santa Clara, CA, USA). There were two group for each ‘Input’ sample and the ‘RIP’ sample for RIP-seq. The RNA sequencing library was constructed by using KC-DigitalTM Stranded mRNA Library Prep Kit for Illumina® (Catalog NO. DR08502, Wuhan Seqhealth Co., Ltd. China) following the manufacturer’s instruction and then sequenced on Novaseq 6000 sequencer (Illumina) with PE150 model. Trimmomatic (version 0.36) was used to filter low-quality reads and trimmed the reads contaminated with adaptor sequences. Then, the de-duplicated sequences were used for protein binding site analysis and were mapped to the reference genome of mus musculus from GRC38 using STAR software (version 2.5.3a) with default parameters. ExomePeak (Version 3.8) software was used for peak calling, and peaks were annotated using bedtools (Version 2.25.0). Then, deepTools (version 2.4.1) was used for peak distribution analysis, and sequence motifs enriched in peak regions were identified using Homer (version 4.10) with default parameters. Gene ontology (GO) analysis for interested genes was done by the Metacape website (http://metascape.org/) with a corrected* P*-value cutoff of 0.05 to judge statistically significant enrichment.

### Immunoprecipitation-mass spectrometry (IP-MS)

Testes from five P10 mice were dissected and lysed in IP lysis buffer with pre-added proteinase inhibitors (Thermo Fisher Scientific). The lysates were centrifuged at 14,000 × g for 20 min. The supernatant was collected and 1/10 volume was used as the input. The rest of the protein was incubated with METTL16 antibody, IgG, or METTL16 antibody plus RNase A for 2 h at 4 °C with gentle rotation. Pre-cleaned magnetic Protein A beads (Invitrogen, Waltham, MA, USA) were added and incubated at 4 °C overnight. The beads were washed with IP lysis buffer and eluted with the elution buffer. The eluted samples were subjected to MS or boiled with 5 ×  SDS loading buffer for western blot analysis. MS experiments were performed on a Q Exactive Plus mass spectrometer coupled to an Easy nLC1200 (Thermo Scientific). High-performance liquid chromatography (HPLC) separation was performed using a self-packed column at a flow rate of 300 nL/min. MS data were then acquired by dynamically selecting the most abundant precursor ions from the survey scan (350-1800 m/z) for fragmentation. MaxQuant (version 1.6.1.0) was used to analyze the MS data and searched against the UniProtKB Rattus norvegicus database. Results were filtered and exported with < 1% false discovery rate (FDR) at the peptide and protein levels, respectively. The experiment was repeated twice. The IP-MS data is provided in Additional file 10: Table S9.

### Ribosome profiling

Testes from seven P10 control or *Mettl16* cKO mice were dissected, immediately placed in liquid nitrogen for 2 h, and transported on dry ice to Genechem (Shanghai, China) for parallel Ribo-seq and RNA-seq. There is one group for ribosome profiling for each 'control' sample (mixture of 14 testes) and the ‘cKO’ sample (mixture of 14 testes). Briefly, samples were treated with a specific lysis buffer containing cycloheximide (50 mg/mL) to obtain the lysate and the concentration of the lysate was measured using the NanoDrop™ 2000 spectrophotometer. To construct a library for Riboseq, unspecific endoribonuclease RNase ǀ was added to digest RNA other than RPFs. Monosomes were isolated using size-exclusion chromatography on a MicroSpin S-400 HR column. The RNA samples were then treated with an rRNA depletion kit and purified with PAGE. cDNA was synthesized and amplified using PCR to construct a library. Samples were sequenced using the Illumina platform, and 150 nt paired-end sequencing was used to obtain 15G raw data. After trimming raw data, clean reads were mapped to the reference genome using TopHat2. The mapped results were quantified at the gene level using HTSeq. Reads per kilobase of transcript per million mapped reads (RPKM) were generated to represent the expression levels of each gene. Parallel RNA-seq was used to analyze the abundance of all transcripts to determine the number of ribosomes associated with individual transcripts after combined analysis with Ribo-seq. Translation efficiency (TE), in the context of cell biology, is the rate of mRNA translation into proteins within cells and was calculated as the ratio of RPKM in Ribo-seq to RPKM in RNA-seq using Xtail.

### Construction of AAV vectors and efferent duct injection

Total RNA was isolated from adult mouse testes and cDNA was prepared using reverse transcription. The full-length open reading frame of *Mettl16* was amplified from the cDNA using PCR with specific primers. A mutant cDNA of *Mettl16* was generated (PP185-186AA, inactivated m6A transferase activity, catalytically dead), and the PCR product was cloned into GV467. An empty vector was used as a negative control. Each vector was cotransfected with the packaging vectors (pHelper and pAAV-RC) into 293 T cells. After 48–72 h, the virus propagation was collected and concentrated. A high-titer of METTL16, mutant, or negative control virus was delivered to mouse testes via efferent duct injection using a micropipette under a microscope. Green dye was added to the virus suspension to monitor the filling of the tubules and approximately 10 µl viral suspension was injected into the rete testis of mouse seminiferous tubules. Two weeks after the injection, the testes were collected for PAS staining to assess their morphology.

### m6A-seq

m6A-seq was performed as previously described [43]. Briefly, total RNA was extracted from one P10 control and *Mettl16* cKO mouse testis, respectively, using TRIzol (Invitrogen, cat. NO 15596026). After DNA digestion with DNase I, RNA quality was determined by examining A260/A280 using the NanodropTM OneC spectrophotometer (Thermo Fisher Scientific Inc), and RNA integrity was confirmed by agarose gel electrophoresis. 10 μg total RNA (control or Mettl16 cKO) was used for polyadenylated RNA enrichment using VAHTS mRNA Capture Beads (VAHTS, cat. NO. N401-01/02) to enrich mRNA. RNA fragments were fragmented (100-200 nt) by ZnCl2 treatment and incubated at 95℃ for 10 min. Among these RNA, 10% RNA fragments were stored as "input," and the rest was later used for m6A immunoprecipitation (IP). Anti-m6A antibody (Synaptic Systems, 202203) was used for m6A immunoprecipitation. RNA samples of both input and IP were prepared using TRIzol. The RNA sequencing library was prepared using the KC-DigitalTM Stranded mRNA Library Prep Kit for Illumina® (Catalog No. DR08502, Wuhan Seqhealth Co., Ltd. China) according to the manufacturer's instructions and finally sequenced on the Novaseq 6000 sequencer (Illumina) using the PE150 model. Raw sequencing data were processed in the same way as RIP-seq. The m6A peaks were annotated using bedtools (version 2.25.0), and deepTools (version 2.4.1) was used for peak distribution analysis with default parameters. Differential m6A peaks were identified using a Fisher test. Sequence motifs enriched in m6A peak regions were identified using Homer (version 4.10), and m6A peaks were visualized using the IGV browser.

### LC-ESI-MS/MS analysis of m6A levels

Total RNA was extracted using TRIzol from P8 control or *Mettl16* cKO mouse testes for MS analysis. Two biological replicates were performed. The concentration and purity of RNA were detected using a Nanodrop ND-1000 (Thermo Fisher Scientific), whereas RNA integrity and genomic DNA contamination were tested using denaturing agarose gel electrophoresis. RNA was digested using nuclease S1, phosphodiesterase, and alkaline phosphatase at 37 °C. The digested sample was extracted with chloroform, and the supernatant was collected in brown autosampler vials for LC-ESI-MS/MS. Nucleosides were separated using an ACQUITY HSS T3 column (Waters, Milford, MA, USA) and detected with a TRAP 6500 (AB SCIEX, Framingham, MA, USA) in ESI^+^ ion multiple reaction monitoring (MRM) mode. Nucleoside-to-base ion mass transitions were used to quantify the modifications, and pure nucleosides were used to generate standard curves. Finally, the m6A level was calculated as a percentage of total unmodified A, as previously described [11].

### Statistical analysis

The data were shown as the mean ± standard error of the mean (SEM). The statistical significance of the differences between the mean values was measured using two-sided Student’s *t*-test with GraphPad Prism 8.0. Data were considered statistically significant at *P < 0.05, **P < 0.01, and ***P < 0.001.

### Supplementary Information

Additional file 1: Figure S1. Conservative analysis of METTL16 among different species and its expression during spermatogenesis. Figure. S2. Generation of germline-specific Mettl16 knockout mouse model. Figure. S3. Loss of METTL16 in testes causes aberrant alternative splicing. Figure. S4. Ribo-seq and RNA-seq analyses of P10 Control and *Mettl16* cKO testes. Figure 5. Related to the data of Figs. 6, 7.

Additional file 2: Table S1. DEGs identified between Control and *Mettl16* cKO mouse testes at P10.

Additional file 3: Table S2. GO term anlsysis of DEGs in *Mettl16* cKO testes identified from RNA-seq data.

Additional file 4: Table S3. GO term analysis of down-regulated genes in P10 *Mettl16* cKO testes identifed from RNA-seq data.

Additional file 5: Table S4. Ribo-seq of Control and *Mettl16* cKO testes at P10.

Additional file 6: Table S5. RIP-seq of METTL16 binding targets using spermatogenic cells from P10 testis.

Additional file 7: Table S6. m6A level identified using LC-ESI-MS/MS from P8 testes.

Additional file 8: Table S7. m6A peaks identified in P10 Control and *Mettl16* cKO testes from m6A-seq data.

Additional file 9: Table S8. Up-regulated and Down-regulated peaks identified in P10 *Mettl16* cKO testes using m6A-seq.

Additional file 10: Table S9. Proteins identified in P10 METTL16 complexes (IP-MS).

Additional file 11: Table S10. The sequences of primers used in this study.

Additional file 12: Table S11. Antibodies used for immunohistochemistry and immunoblot in this study.

Additional file 13. Uncropped images for the blots.

Additional file 14. Review history.

## Data Availability

The RNA-seq datasets are available in the NCBI SRA database under accession number PRJNA1019929 (www.ncbi.nlm.nih.gov/sra/?term=PRJNA1019929) [44], Ribo-seq datasets are available in the NCBI SRA database under accession number PRJNA1022765 (www.ncbi.nlm.nih.gov/sra/?term=PRJNA1022765) [45], m6A-seq datasets are available in the NCBI SRA database under accession number PRJNA1020634 (www.ncbi.nlm.nih.gov/sra/?term=PRJNA1020634) [46] and RIP-seq datasets are available in the NCBI SRA database under accession number PRJNA1071981 (https://www.ncbi.nlm.nih.gov/sra/?term=PRJNA1071981) [47]. The study additionally used several public datasets from NCBI GEO datasets including single-cell RNA-sequencing of adult wild-type mouse spermatogenic cells (www.ncbi.nlm.nih.gov/geo/query/acc.cgi?acc=GSE109033) [22] and wildtype postnatal 7 days testicular cells (www.ncbi.nlm.nih.gov/geo/query/acc.cgi?acc=GSE117708) [23]. Raw scripts used in the paper can be found from ZENODO (10.5281/zenodo.12608411) [48]. Uncropped versions of our microscopy images are deposited on Figshare (10.6084/m9.figshare.26143246) [49].
