# Supplementary material for: N6-methyladenosine writer METTL16-mediated alternative splicing and translation control are essential for murine spermatogenesis
Source: Genome Biol. 2024 Jul 19;25:193. doi: 10.1186/s13059-024-03332-5 (PMC11264951; doi:10.1186/s13059-024-03332-5)
Supplement: Supplementary file 1 — Additional file 1: Figure S1. Conservative analysis of METTL16 among different species and its expression during spermatogenesis. Figure. S2. Generation of germline-specific Mettl16 knockout mouse model. Figure. S3. Loss of METTL16 in testes causes aberrant alternative splicing. Figure. S4. Ribo-seq and RNA-seq analyses of P10 Control and Mettl16 cKO testes. Figure 5. Related to the data of Figs. 6, 7. [file 13059_2024_3332_MOESM1_ESM.pdf]

# Figure. S1

**A**

| | Frog | Zebrafish | Chick | Mouse | Human | Bovine |
|---|---|---|---|---|---|---|
| Frog | 100 | 72.53 | 71.22 | 71.59 | 72.74 | 71.82 |
| Zebrafish | 72.53 | 100 | 76.92 | 75.43 | 75.05 | 74.15 |
| Chick | 71.22 | 76.92 | 100 | 76.36 | 76.88 | 75.45 |
| Mouse | 71.59 | 75.43 | 76.36 | 100 | 89.15 | 88.79 |
| Human | 72.74 | 75.05 | 76.88 | 89.15 | 100 | 91.09 |
| Bovine | 71.82 | 74.15 | 75.45 | 88.79 | 91.09 | 100 |

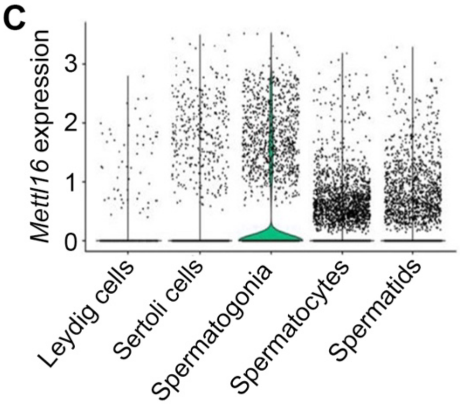


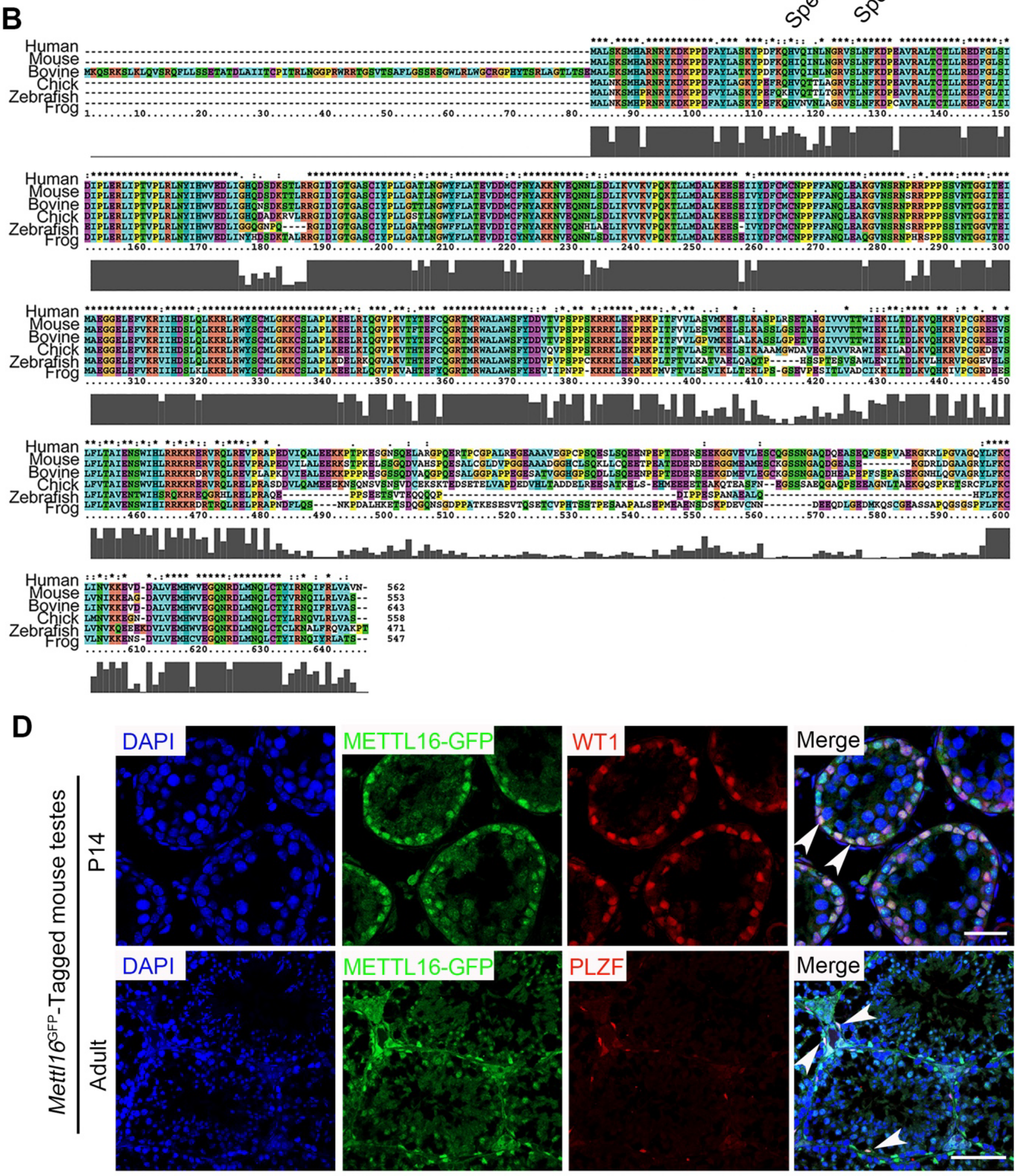

**Figure S1. Conservative analysis of METTL16 among different species and its expression during spermatogenesis. (A)** The heatmap for the similarity of METTL16 amino acid sequences among human, mouse, bovine, chick, zebrafish, and frog. Red and blue colors represent high and low similarities, respectively. **(B)** The multiple alignments of the amino acid sequences of METTL16 from various species. **(C)** Expression of Mettl16 in different types of germ cells is obtained by reanalysis of single-cell sequencing data (GSE112393). **(D)** Representative images of co-immunofluorescent staining of GFP (green) and WT1 (P10 testes, red) or PLZF (adult testes, red) on testicular sections from Mettl16EGFP mice are shown. The nuclei were stained with DAPI (blue). Scale bar = 20 μm for P14 and Scale bar = 50 μm for adult.

## Figure. S2

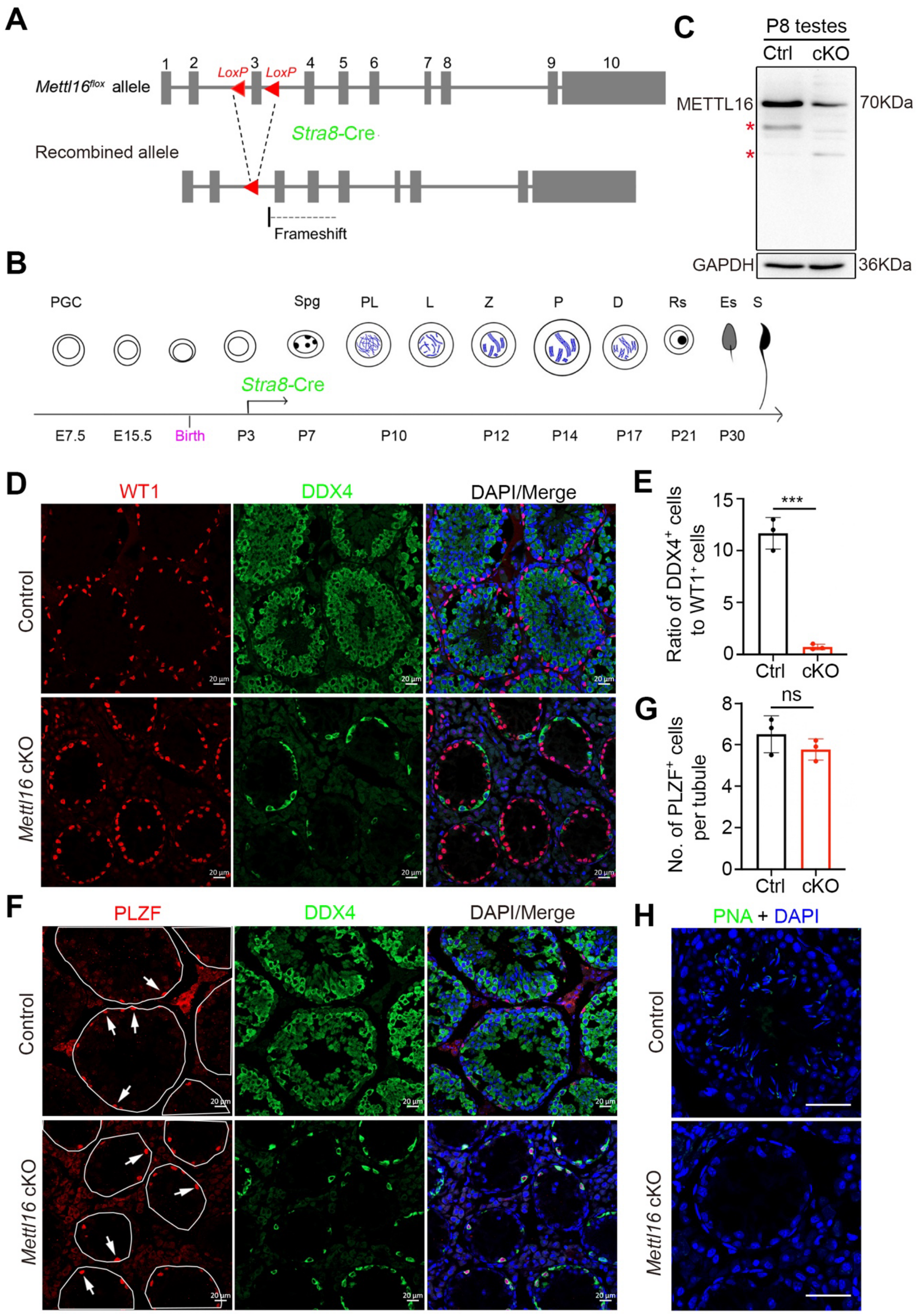
A
Mettl16flox allele
LoxP
LoxP
1 2 3 4 5 6 7 8 9 10
Stra8-Cre
Recombined allele
Frameshift
B
PGC
Spg
PL
L
Z
P
D
Rs
Es
S
Stra8-Cre
E7.5
E15.5
Birth
P3
P7
P10
P12
P14
P17
P21
P30
C
P8 testes
Ctrl
cKO
METTL16
70KDa
GAPDH
36KDa
D
WT1
DDX4
DAPI/Merge
Control
Mettl16 cKO
E
Ratio of DDX4+ cells to WT1+ cells
***
Ctrl
cKO
F
PLZF
DDX4
DAPI/Merge
Control
Mettl16 cKO
G
No. of PLZF+ cells per tubule
ns
Ctrl
cKO
H
PNA + DAPI
Control
Mettl16 cKO

**Figure. S2. Generation of germline-specific *Mettl16* knockout mouse model. (A)** The schematic strategy of the *Mettl16*$^{flox/flox}$ mouse model construction is shown. **(B)** The chronological events during spermatogenesis in mice are shown. *Stra8*-Cre starts expression at P3 testes in mice. **(C)** METTL16 expression in Control and *Mettl16* cKO mice at P8 is analyzed by Western blot. GAPDH was used as a loading control. The asterisks represent the non-specific band (present in both Ctrl and cKO) **(D)** Representative images of co-immunofluorescent staining of DDX4 (green) and WT1 (red) on testicular sections from adult Control and *Mettl16* cKO mice are shown. The DNA was stained with DAPI (blue). Scale bars = 20 μm. **(E)** The histogram shows the quantification of the ratio of DDX4$^{+}$ cells to WT1$^{+}$ cells in (D). Data were presented as mean ± SEM. n=3. ***$P$ < 0.001. **(F)** Representative images of co-immunofluorescent staining of DDX4 (green) and PLZF (red) on testicular sections from adult Control and *Mettl16* cKO mice are shown. The DNA was stained with DAPI (blue). Arrows indicate PLZF$^{+}$ spermatogonia in the tubules. Scale bars = 20 μm. **(G)** The histogram shows the quantification of the number of PLZF$^{+}$ cells per tubule in (F). Data were presented as mean ± SEM. n=3. ns, not significant. (**H)** Representative images of immunofluorescent staining of PNA (green) on testicular sections from adult Control and *Mettl16* cKO mice are shown. The nuclei were stained with DAPI (blue). Scale bars = 50 μm.

## Figure. S3

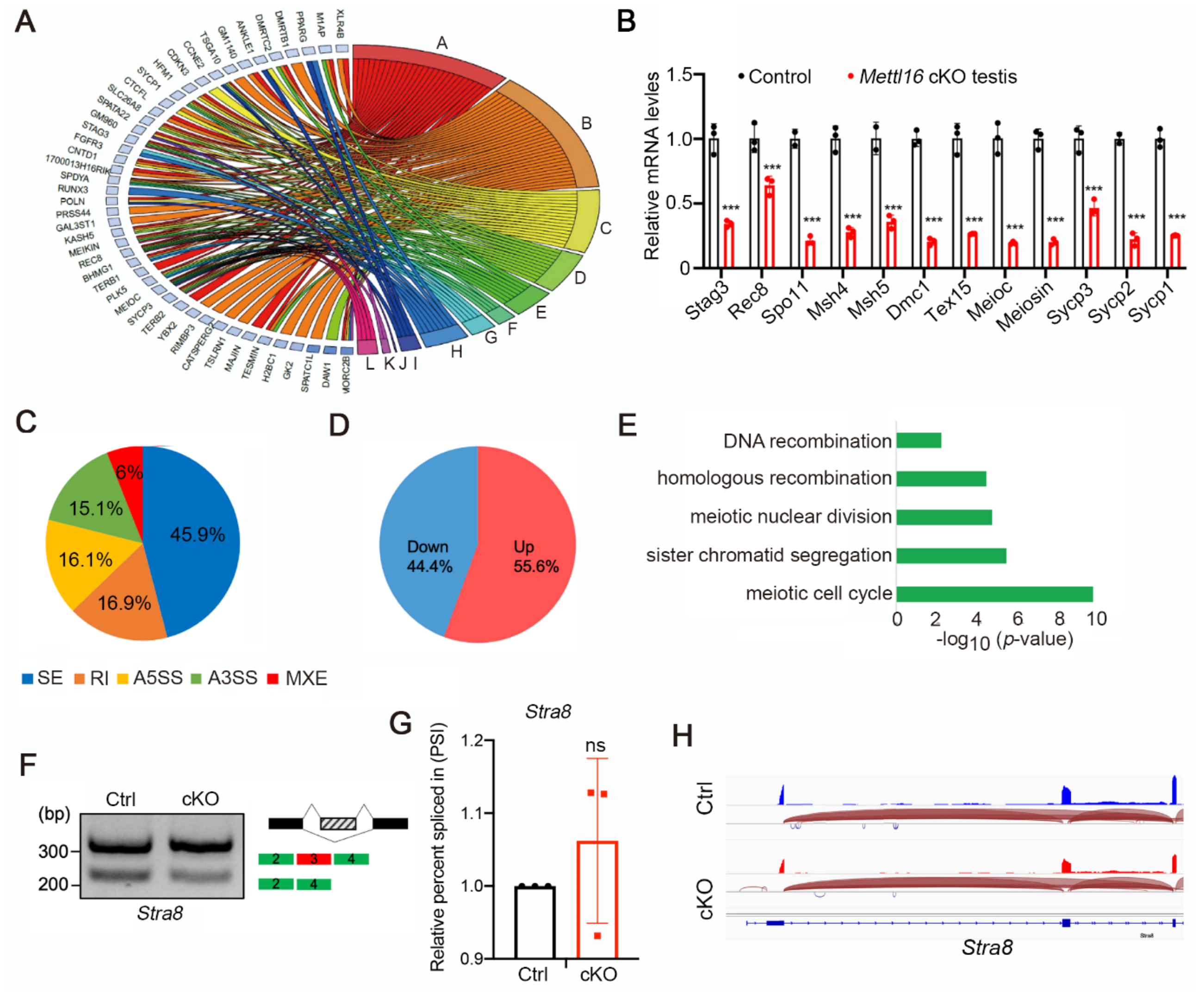


**Figure. S3. Loss of METTL16 in testes causes aberrant alternative splicing. (A)** The circle plot displays down-regulated genes (*Mettl16* cKO *v.s.* Control) enriched in GO terms. Among these, 'A' indicates meiotic cell cycle, 'B' indicates gamete generation, 'C' indicates DNA metabolic process, 'D' indicates sister chromatid segregation, 'E' indicates male meiotic nuclear division, 'F' indicates synaptonemal complex organization, 'G' indicates regulation of cell cycle progress, 'H' indicates regulation of chromosome segregation, 'I' indicates DNA replication, 'J' indicates meiotic DNA double-strand break formation, 'K' indicates DSB repair, 'L' indicates sister chromatid cohesion. **(B)** RT-qPCR analysis using RNA extracted from P10 testes verifies the indicated downregulated meiosis-related genes from RNA-seq data. Data are presented as mean ± SEM, n=3. ****P* < 0.001. **(C)** Pie chart showing the distribution of changed AS among different splicing events. **(D)** Pie chart representing proportions of changed AS identified in Control and *Mettl16* cKO mouse testes. **(E)** GO term enrichment analysis of biological processes for the overlapped 91 genes between DEGs and AS events in *Mettl16* cKO versus control testes. **(F)** RT-PCR analysis for *Stra8* gene in c-KIT-positive spermatogonia isolated from Control and *Mettl16* cKO mice at P10. The right panel

represents the schematic diagram of indicated AS exons. **(G)** The quantification of percent spliced in (PSI). Data are presented as mean ± SEM, n = 3. Ns, no significance. **(H)** Gene track view of splicing of *Stra8* in P10 control and *Mettl16* cKO RNA-seq data.

## Figure. S4

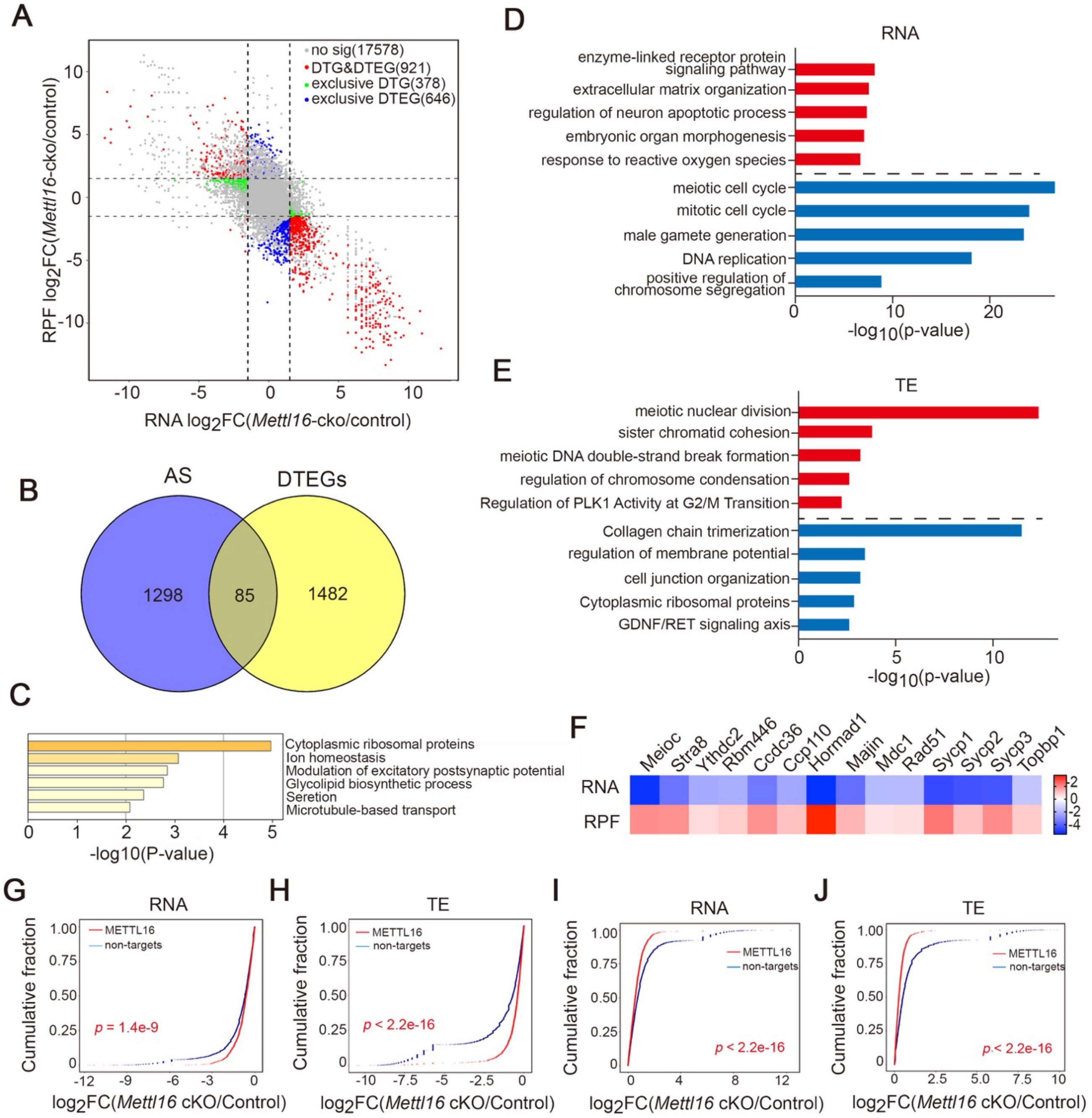


**Figure. S4. (A)** Scatter plots show the combined analysis of Ribo-seq and RNA-seq in P10 Control and *Mettl16* cKO testes. Genes were classified according to the indicated criteria. $|Log_2FC|$ or $|Log_2TE|$ > 1 and an adjusted *P*-value < 0.05 were considered significant. **(B)** Venn diagrams show the overlap between AS events and DTEGs (differential translation efficiency genes) in Mettl16 cKO versus control testes. **(C)** GO term enrichment analysis of biological processes for the overlapped 85 genes between AS events and DTEGs in Mettl16 cKO versus control testes **(D)** GO term enrichment analysis of differentially transcribed genes (DTG) shows the top 5 terms enriched in down (blue) and up (red) regulated genes. **(E)** GO term enrichment analysis of differential translation efficiency genes (DTEG) shows the top 5

terms enriched in down-regulated (blue) and up-regulated (red) translation efficiency genes. **(F)** The heatmap displays the fold-change of RNA and ribosome-protected fragments (RPF) abundance for 14 representative meiosis-related genes from Ribo-seq data. The red color and blue color refer to the fold changes that being upregulated and downregulated, respectively. **(G-H)** Cumulative distribution of RNA abundance (G) and translation efficiency (H) changes of down-regulated genes ‘Log2FC(cKO/Ctrl) < 0’ between Control and *Mettl16* cKO testes. **(I-J)** Cumulative distribution of RNA abundance (I) and translation efficiency (J) changes of up-regulated genes ‘Log2FC(cKO/Ctrl) > 0’ between Control and *Mettl16* cKO testes. The blue curves indicate non-targets of METTL16, and the red curves indicate METTL16-RIP. *P*-values were calculated using a two-sided Wilcoxon test.

**Figure. S5**

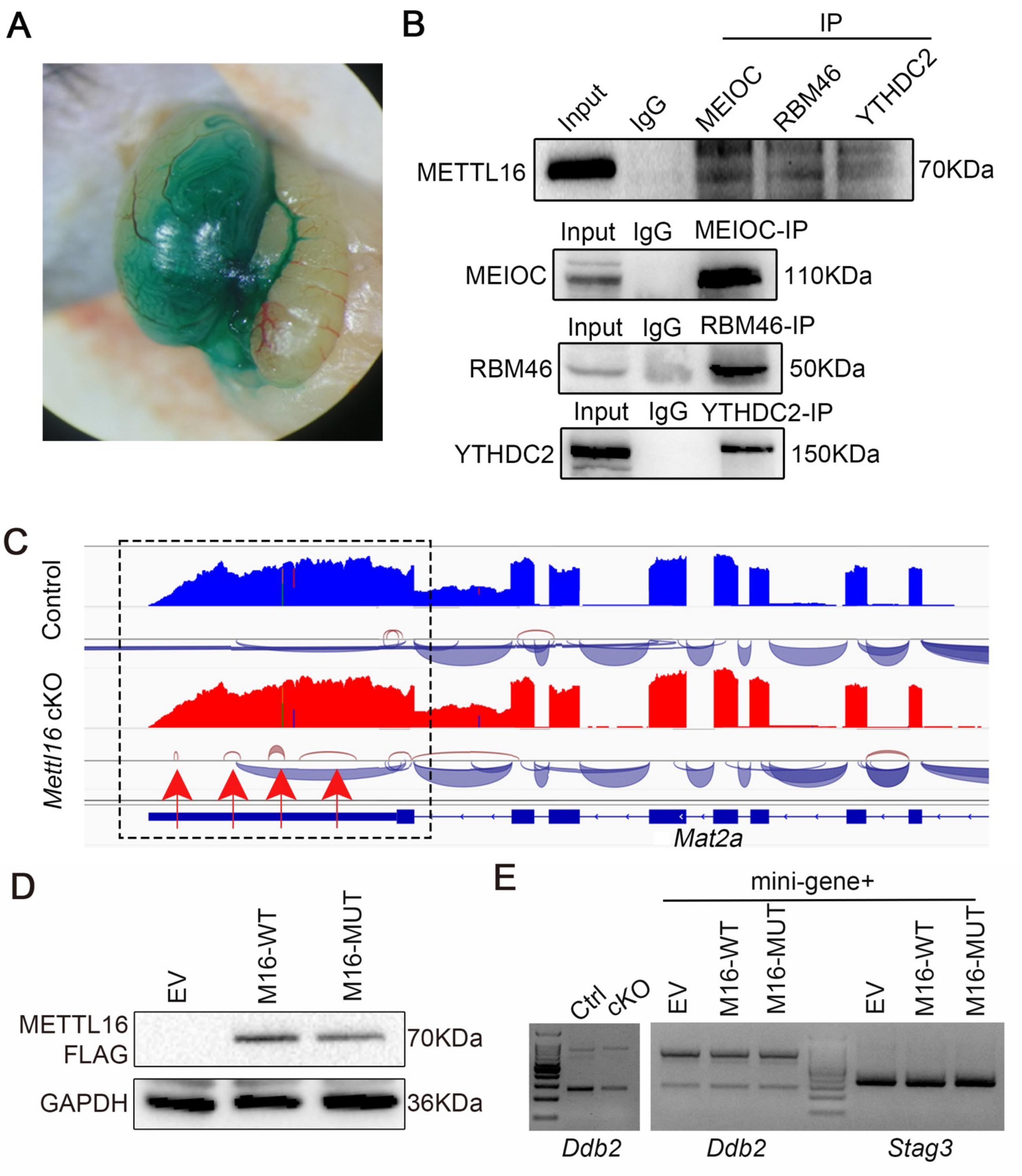


**Figure. S5. (A)** Green dye was added to the virus suspension to monitor the success of injection in the seminiferous tubules. (**B**) Reciprocal IP using antibodies against MEIOC, YTHDC2, and RBM46 of P10 wild-type testes were shown. **(C)** Gene track view of 3'-UTR splicing of *Mat2a* in P10 control and *Mettl16* cKO RNA-seq data. Red arrows indicate A3SS events in the *Mat2a* 3'-UTR region. **(D)** The expression of METTL16-Flag in HEK293T cells

was detected by western blot after transfection of empty vector (EV), wild-type or mutated METTL16-FLAG overexpression vector into HEK293T cells. **(E)** Left panel displayed RT-PCR analysis for *Ddb2* in c-KIT-positive spermatogonia isolated from Control and *Mettl16* cKO mice at P8. Right panel displayed RT-PCR analysis for *Ddb2* and *Stag3* in HEK293T cells following transfected mini-gene (pcDNA-*Ddb2* or pcDNA-*Stag3*) and EV, wild-type or mutated METTL16-FLAG vector respectively.
