# Supplementary material for: N6-methyladenosine writer METTL16-mediated alternative splicing and translation control are essential for murine spermatogenesis
Source: Genome Biol. 2024 Jul 19;25:193. doi: 10.1186/s13059-024-03332-5 (PMC11264951; doi:10.1186/s13059-024-03332-5)

Fig1E

IB:METTL16

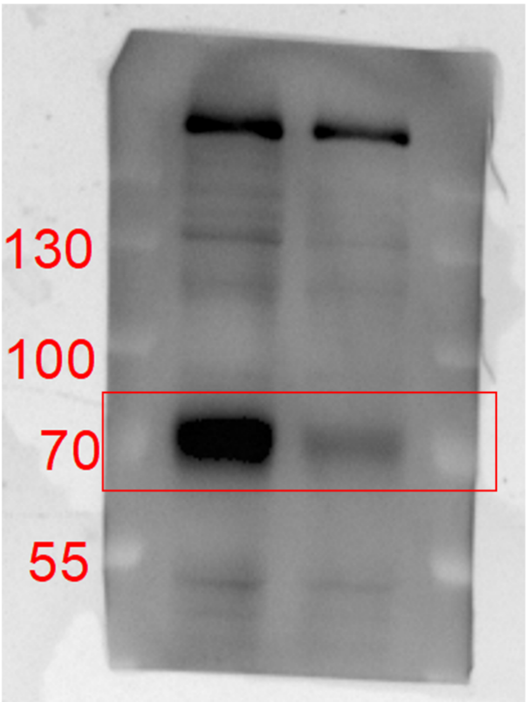

IB:GAPDH

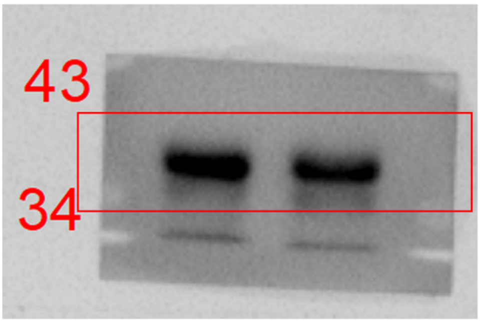

Fig2E

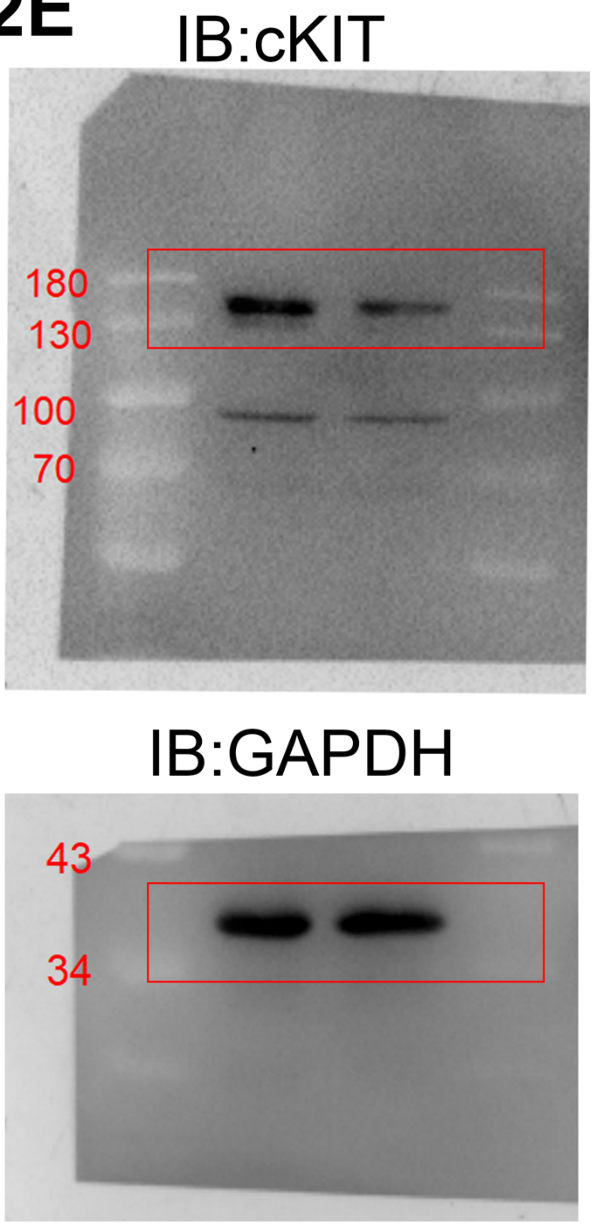

Fig2E

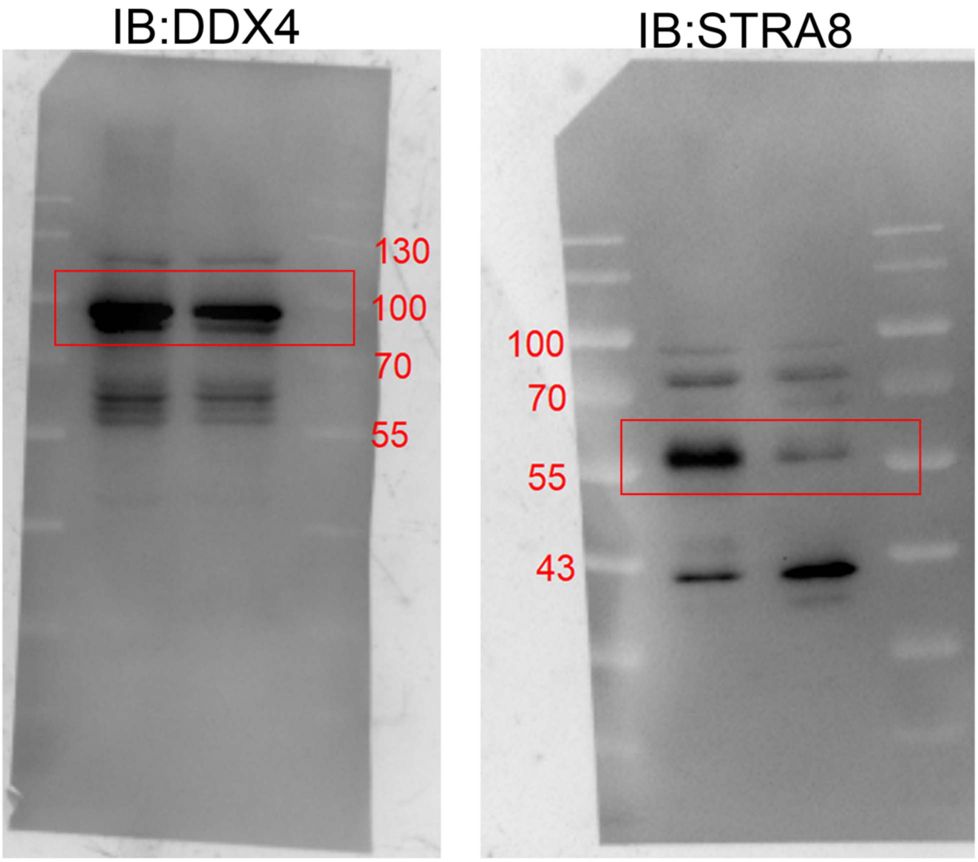

Fig4G

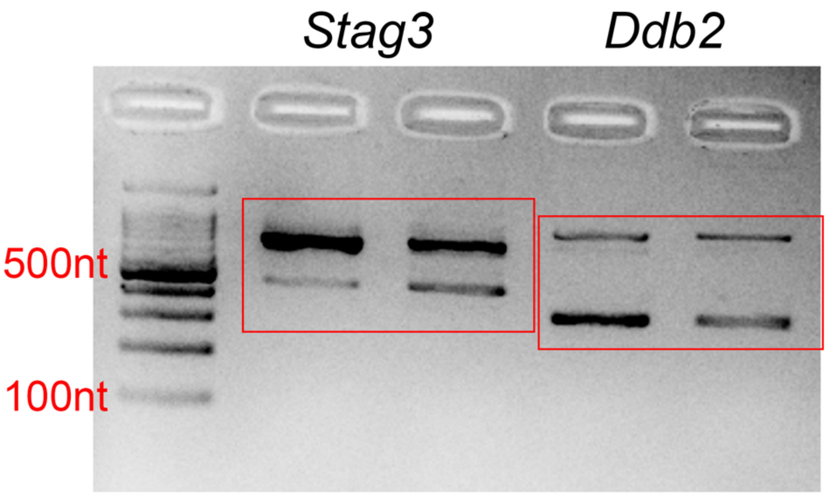

Fig4H

IB:METTl16

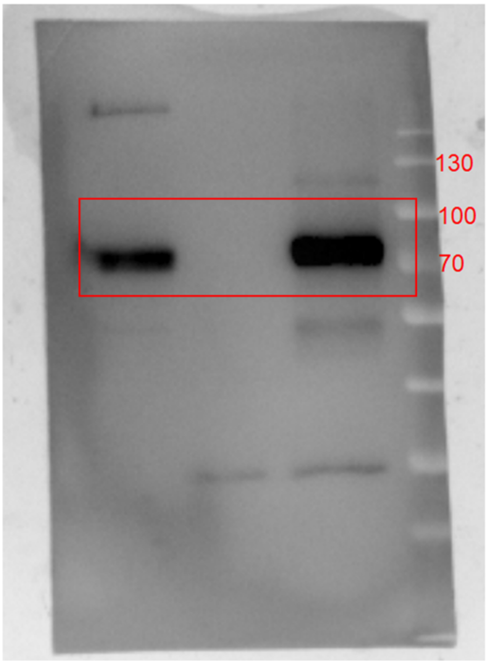

Fig4l

IB:METTL16

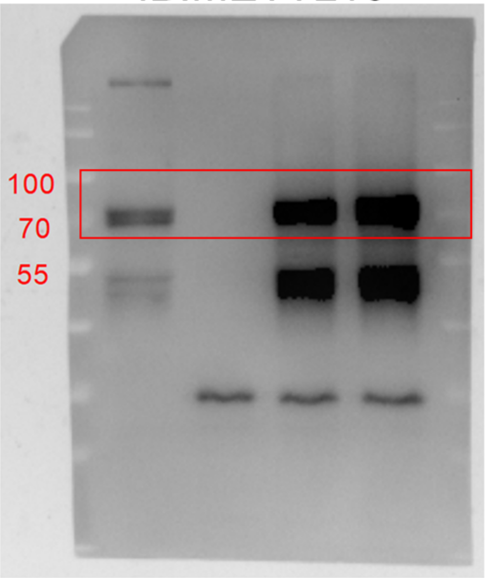

IB:SF3B1

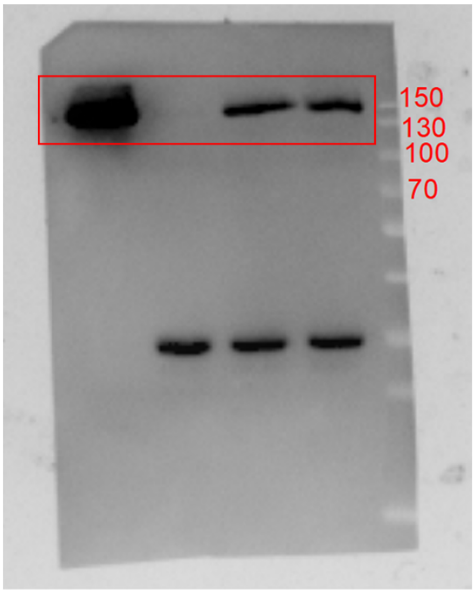

IB:SF3B3

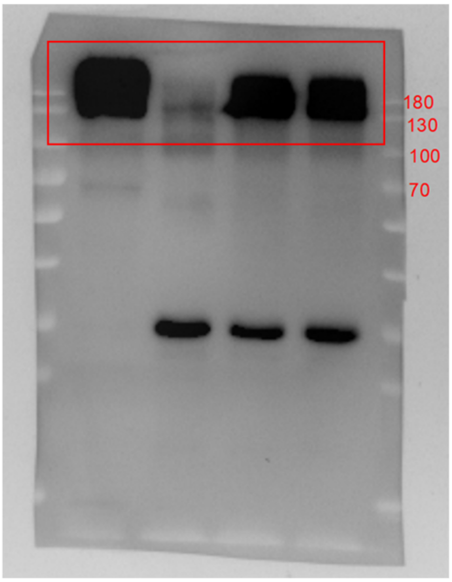

Fig7C

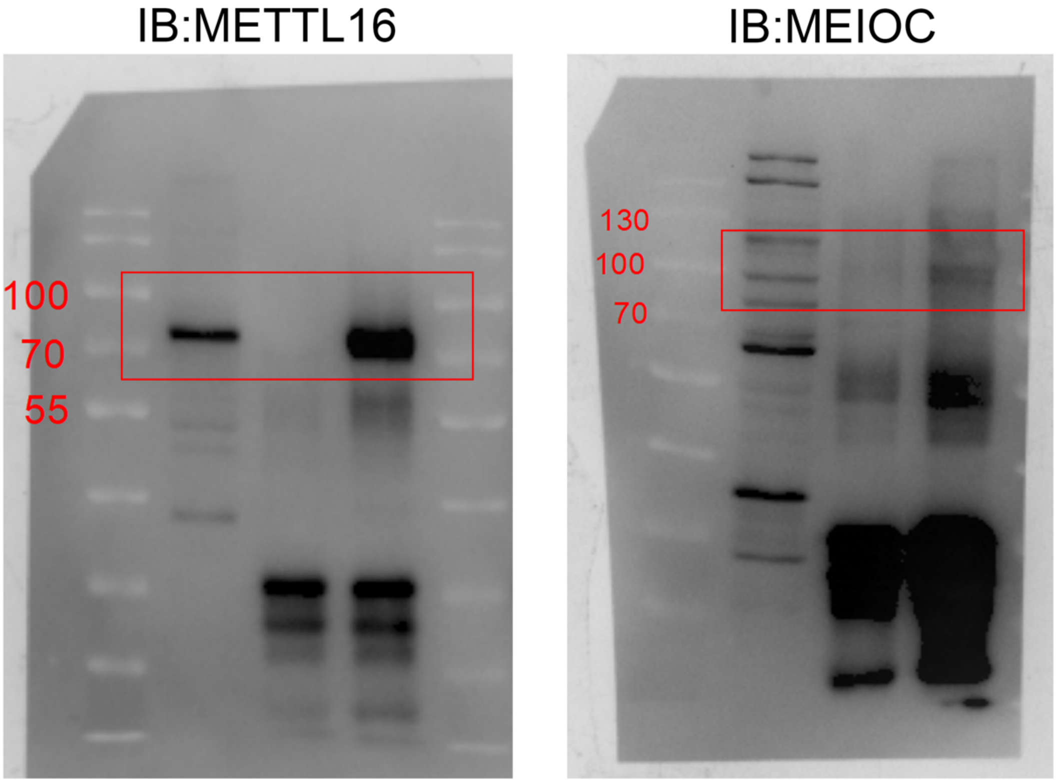

Fig7C

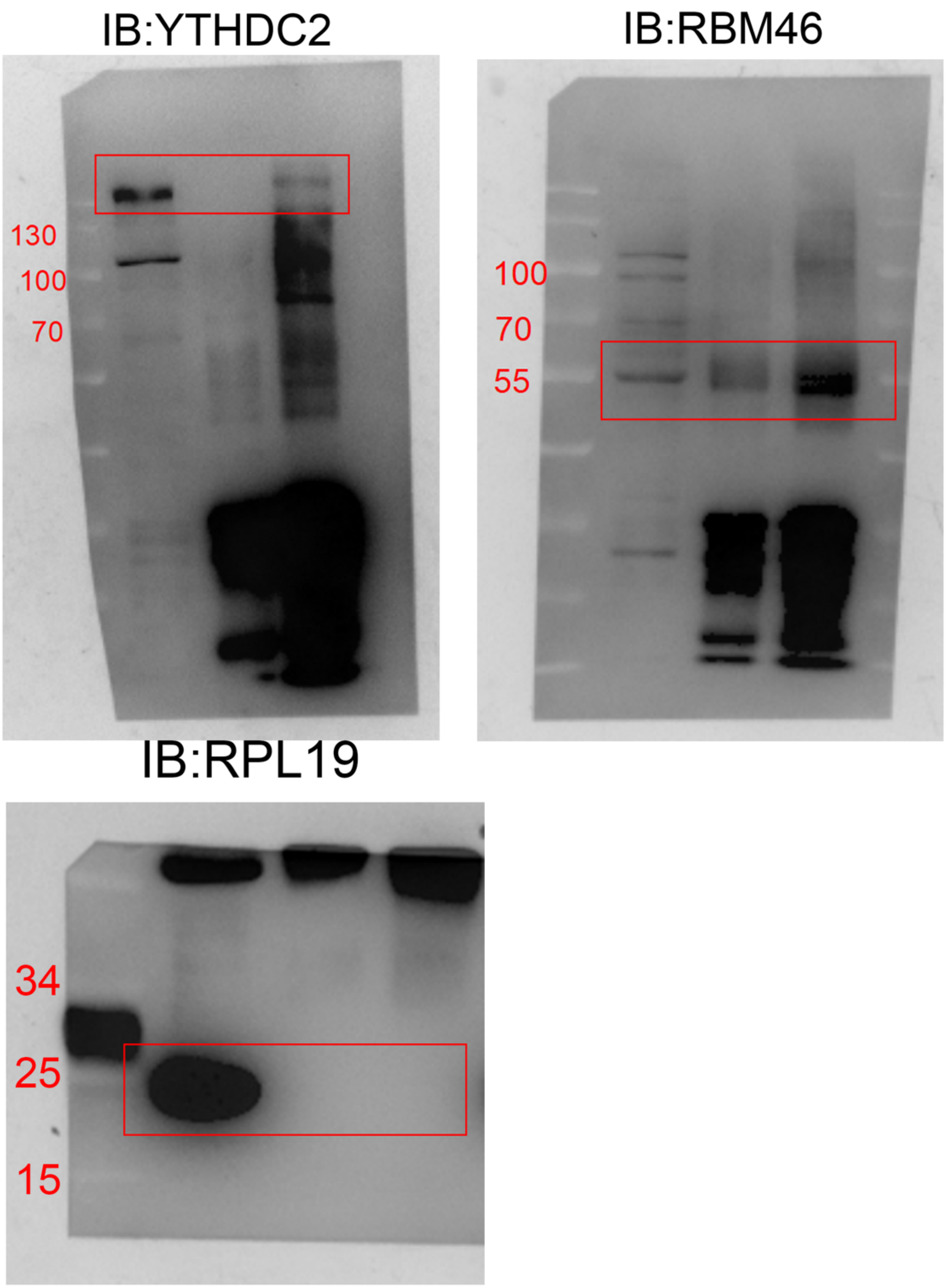

Fig7D

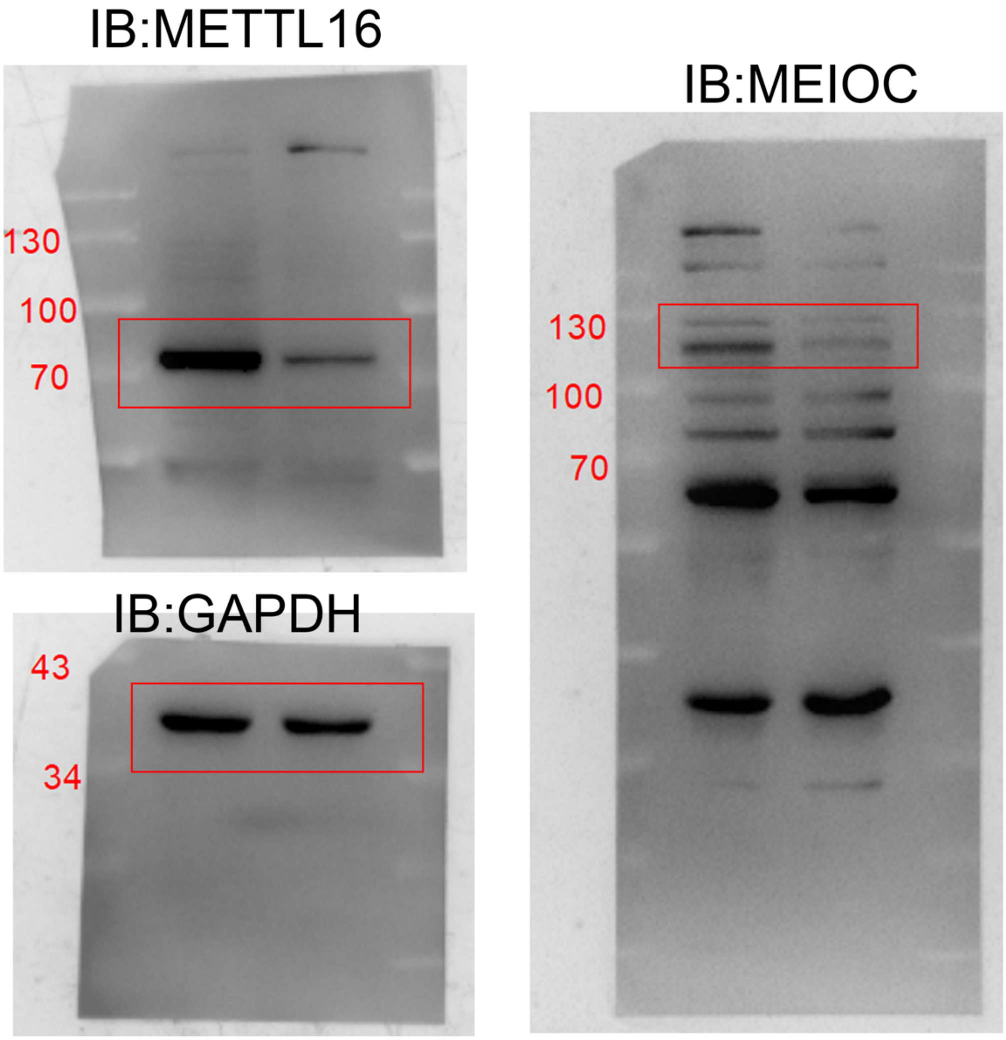

Fig7D

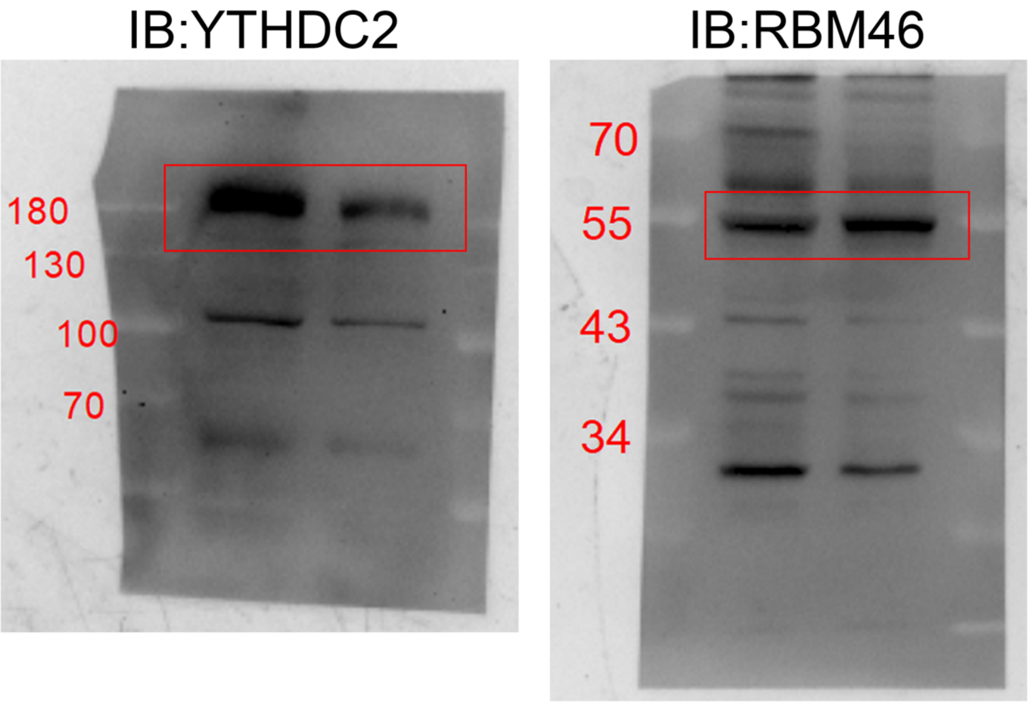

FigS2C

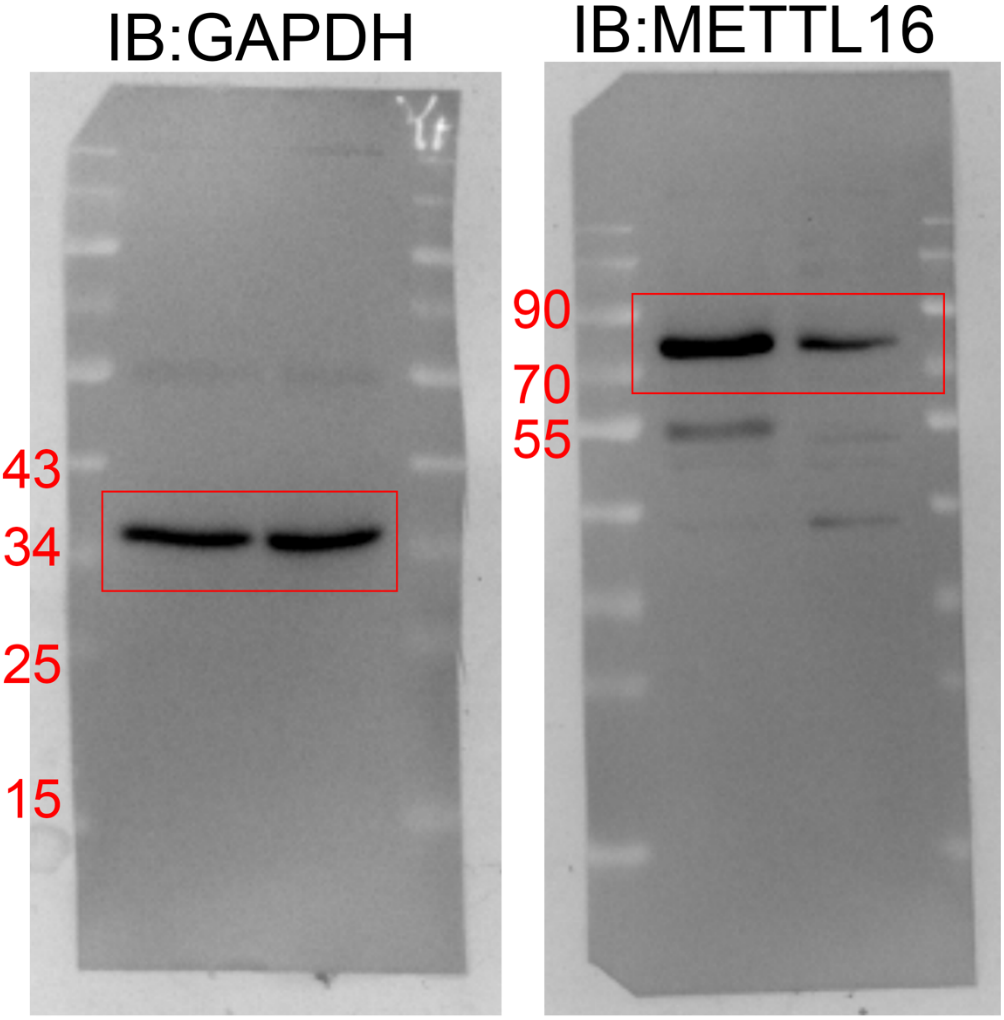

FigS5B

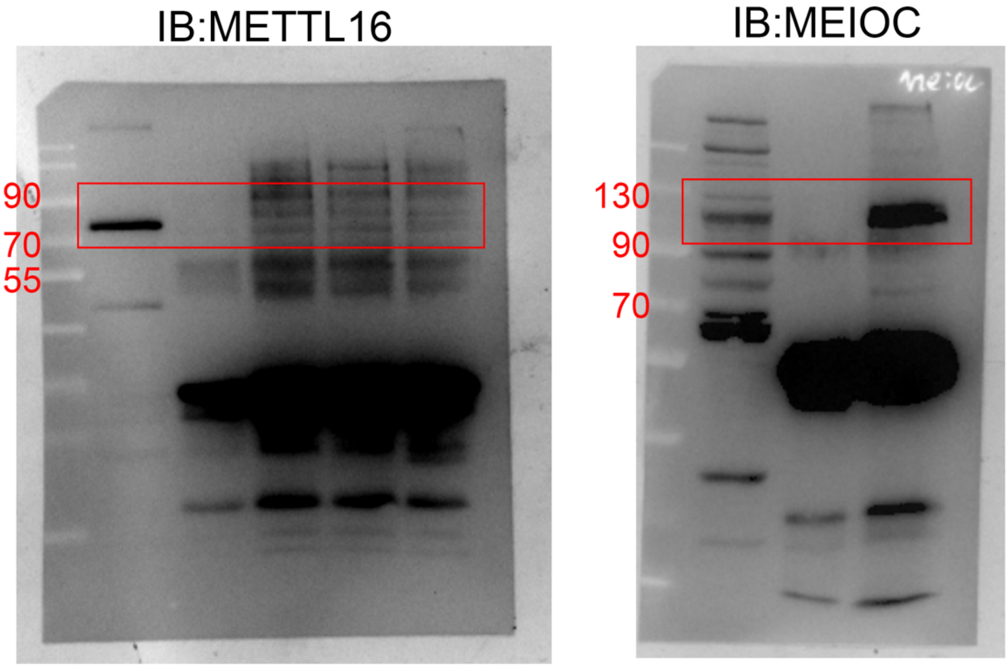

FigS5B

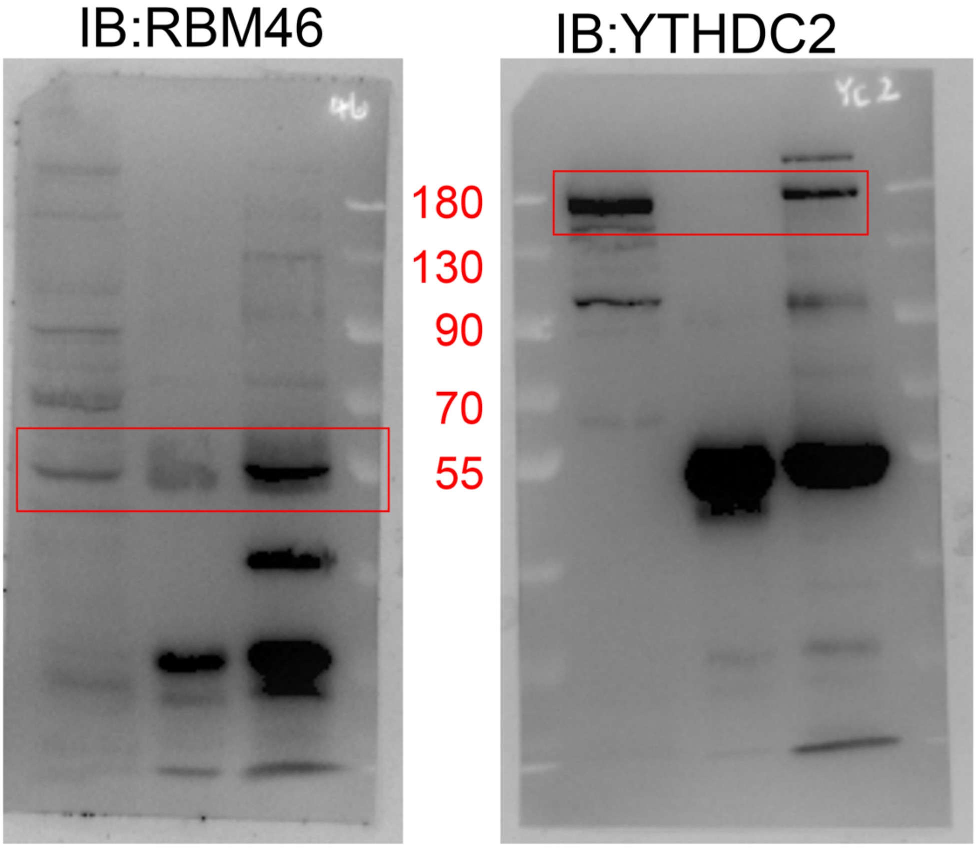

FigS5D

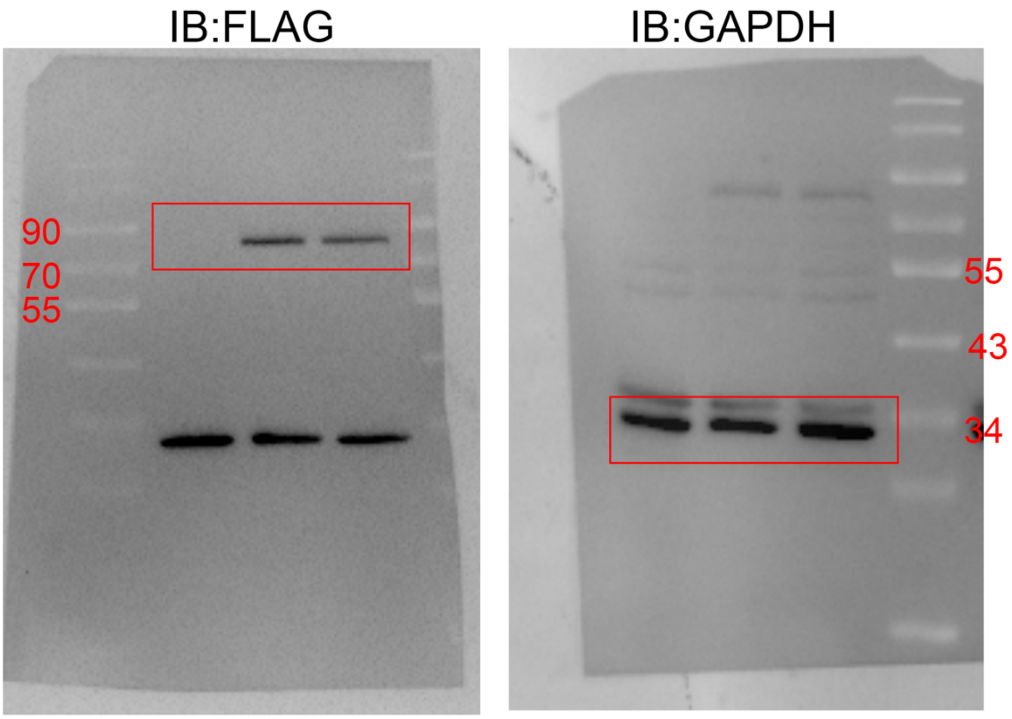

FigS5E

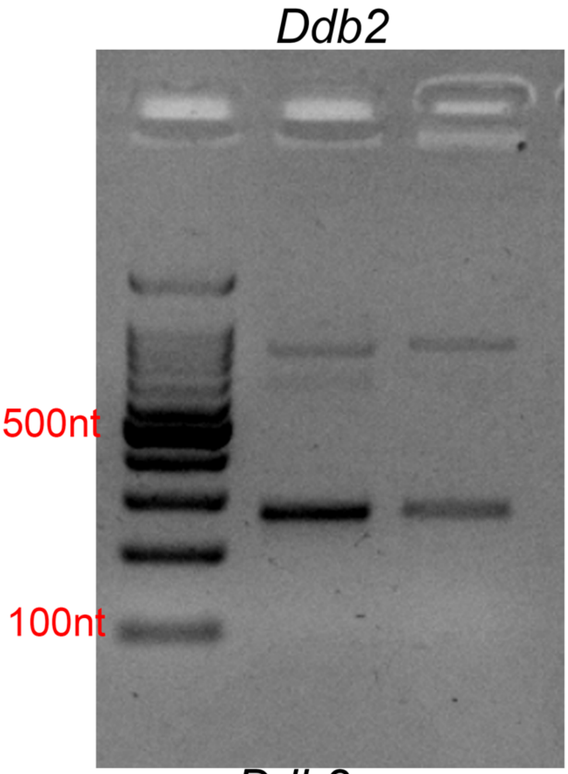

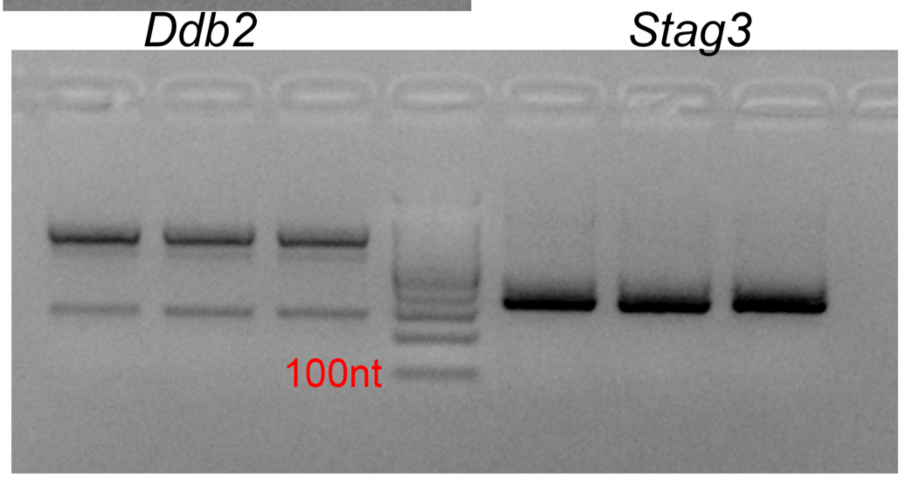

Supplement: Supplementary file 13 — Additional file 13. Uncropped images for the blots. [file 13059_2024_3332_MOESM13_ESM.pdf]
