# Supplementary material for: N6-methyladenosine writer METTL16-mediated alternative splicing and translation control are essential for murine spermatogenesis
Source: Genome Biol. 2024 Jul 19;25:193. doi: 10.1186/s13059-024-03332-5 (PMC11264951; doi:10.1186/s13059-024-03332-5)
Supplement: Supplementary file 14 — Additional file 14. Review history. [file 13059_2024_3332_MOESM14_ESM.docx]

Review History

**First round of review**

**Reviewer 1**

**Were you able to assess all statistics in the manuscript, including the appropriateness of statistical tests used?**

Yes, and I have assessed the statistics in my report.

**Comments to author:**

In this manuscript, the authors suggest a molecular model wherein METTL16-mediated alternative splicing and translation regulation play a crucial role in determining the mitosis-to-meiosis transition in germ cell fate in mice. This paper, well written and have sufficient details, is of outstanding interest to a broad audience of biologists. While the study offers valuable insights, I have identified both major and minor concerns that, if addressed, could further enhance the quality and clarity of this research.

Regarding splicing regulation by METTL16:
* There is a lack of figures illustrating alternative splicing changes for Mat2a (Line 273) following the loss of METTL16. Existing studies highlight METTL16's role in regulating MAT2A splicing in C. elegans (Mendel et al., Cell 2021) and human cells (Pendleton et al., Cell 2017) via either its methyltransferase or non-methyltransferase activities. Furthermore, I am wondering this Mat2a splicing changes in mice are due to METTL16's catalytic function or not?
* In Line 277, the suggestion that METTL16 may repress alternative splicing in the testes warrants clarification. How many splicing changes correlate with METTL16 binding or m6A peaks?
* U6 snRNA, a known METTL16 target essential for splicing, influences the splicing of specific 5' splice sites in yeast and plant studies (Ishigami et al., Nat Commun 2021; Parker et al., eLife 2022) but remains unexplored in mammalian cells. One of the interesting questions is that whether this 5' splice sites bias are also shown in mammalian cells. The author's proposal of METTL16 interaction with splicing regulator SF3B1/3 raises questions regarding splicing changes attributed to METTL16/U6 versus METTL16/SF3B. Utilizing established cell lines expressing catalytic mutant METTL16 could help differentiate splicing changes influenced by its catalytic versus non-catalytic functions.

Regarding translation regulation by METTL16:
* In Line 308, the authors conclude that the translation efficiency of METTL16 targets was lower than non-targets in METTL16-deficient testes. In the above paragraph, they showed that the direct targets of METTL16, Stra8 and Meioc, are translated more in METTL16-deficient testes though their mRNA abudance become lower. The conclusion and their examples appear to be contradictory.
* Via biochemistry experiments, the authors found that METTL16 interacts with MEIOC/YTHDC2/RBM46. However, the evidence for this interaction functionally regulating transcription and translational is lacking in this manuscript.

Meanwhile, I have several minor points that I hope can be helpful for this study.
* The authors observed that m6A-sequencing showed that ablation of METTL16 caused upregulation of the m6A-enriched transcripts and downregulation of the m6A-depleted transcripts. However, the authors also observed that METTL16 knockout causes very minor global m6A changes (by MS and m6A-seq). (1) Can the authors plot m6A levels on U6 RNA with and without METTL16? This could be used as a proof-of-concept. (2) As the majority of m6A on mRNA are not installed by METTL16 and they stayed unchanged with and without METTL16, how does the expression level of m6A-enriched transcripts are upregulated whilst m6A-depleted transcripts are downregulated due to loss of METTL16?
* In Figure 7D, why is METTL16 protein still present in the Mettl16 cKO samples by WB?
* What's the rational for global m6A level quantification of Mettl16 cKO using total RNA and mass spectrometry, rather than poly(A)+ RNA, rather than poly(A)- RNA? I believe that the authors are clear that rRNA is dominant population (>90%) of total RNA, the m6A on which are installed by other methyltransferases.
* It's unclear what does "but failed" mean in Line 329.
* I would suggest the authors plot their m6A enrichment, shown in figure 6D, in a manner consistent with literature using metaPlotR (https://github.com/olarerin/metaPlotR) or RNAmpp (https://github.com/guifengwei/RNAmpp).

**Reviewer 2**

**Were you able to assess all statistics in the manuscript, including the appropriateness of statistical tests used?**

Yes, and I have assessed the statistics in my report.

**Comments to author:**

Qian Ma et al. have generated testes- specific conditional Mettl16 KO mice to investigate its role in spermatogenesis. They have performed an in-depth analysis of the testes in these mice, including histochemical analyses, immunoblotting, RNAseq, RIP-seq, meRIP-seq and determined the translation efficiency by ribosome profiling.
Overall, they obtained some very interesting results suggesting a role of Mettl16 in the progression of meiosis and alternative splicing, the latter of which had been described before in different systems.
Rescue experiments suggested that the catalytic activity of Mettl16 was required for its function in spermatogenesis. However, Mettl16-specific m6A sites could not be linked to alternatively spliced potential target transcripts, so overall it is not quite clear how Mettl16 activity mechanistically relates to the multiple observations described by the authors (binding of splicing factors, alternative splicing of several transcripts, interaction MEIOC/RBM46/YTHDC2 complex, differential methylation of select transcripts,…).

Major points:

Line 116: the authors should mention that a germ-cell specific conditional KO mouse model was generated and described in Mendel et al. 2021, also describing infertility, high Mettl16 expression in testes and decreased testes size. They should discuss the advantages or additional information yielded by analyzing Stra8-Cre conditional KO mice.

The quality of the immunoblot in Fig.1A is nor adequate. Please provide a better blot image. Please provide the age of the mice used for immunoblotting in the figure legend.

Figure S2F please provide more representative images of PLZF+ cells in tubule sections.

Please provide a more detailed description of the results and legend presented in Fig. 3G. The description in the legend seems to refer to the cKO only.

Fig. 6I Please provide WT control as comparison for the partial rescue.

Fig. 7C The IP bands for MEIOC (unspecific IgG binding and immunoprecipitated by anti-Mettl16 antibodies) seem to display a different electrophoretic mobility. How do the authors explain this difference?
Please provide immunoblots of a non-binding protein (e.g. a YTH-domain containing protein not identified in the interactome) as control for the specificity of the co-IP.

To better dissect which of the observed phenotypes are causal for the physiological consequences which are not rescued by catalytically inactive Mettl16, the authors should perform select experiments (e.g. RT-PCR for alternative splicing of Stra8, Stag3) in the cKO testes injected with AAV expressing WT or PP185-186AA Mettl16.

In general, the authors should provide more detailed method descriptions (e.g. for m6A-seq, ribosome profiling, MS) or cite publications adequately describing the methods the authors employed.
Minor points:
Lines 141 to 148. The relevance and novelty of this paragraph in the beginning of the results section (including supplementary figure 1A/B) is unclear to this reviewer. Please integrate into a paragraph where the alignment and high degree of conservation strengthens the presented results.

Line 217: ..was blocked…

**Reviewer 3**

**Were you able to assess all statistics in the manuscript, including the appropriateness of statistical tests used?**

Yes, and I have assessed the statistics in my report.

**Comments to author:**

Mettl16 encodes an evolutionary conserved m6A methyltransferase which is ubiquitously expressed. It has been shown to catalyze m6A methylation of U6 snRNA and of the S-Adenosylmethionine (SAM) synthetase MAT2A mRNA. Its role during spermatogenesis, a process in which transcriptomic complexity is high and post-transcriptional regulation is very dynamic, has not yet been addressed.

In their manuscript (GBIO-D-23-01720) Ma et al. presents the phenotypic and molecular consequences of the conditional knockout of Mettl16 in postnatal male germ cells. The authors used Stra8-Cre (as part of the CRE/LOX system), a construct known to express the Cre recombinase in spermatogonia from postnatal day3. They first show that KO males are infertile due to a premeiotic defect in the differentiation of spermatogonia. As a result, KO testes from day 10 show a striking loss of germ cells compared to testes from control males of the same age. The number of differentiating (c-kit+) spermatogonia is severely reduced, and almost no meiotic cells are detected (while at this stage, pre-leptotene meiotic cells are normally present). The authors conclude that spermatogonia differentiation and meiotic initiation are impaired by Mettl16 KO.

The authors then performed RNA-seq and ribo-seq on P10 whole testis from KO vs control males and concluded that METTL16 deletion alters expression of meiosis-related genes, with the identification of >1000 differentially transcribed genes and >1000 genes with altered translation efficiency. They also investigate alternative splicing using rMATS tool and identified many alternative splicing events between KO and CTL testis. They compared their data with P8 testes m6A-IP libraries, and also performed m6A-seq in P10 testes from KO and CTL. Performing "rescue" experiment they show that m6A methyltransferase activity of METTL16 is necessary for its function during spermatogenesis. Finally, the authors conducted RIP-seq experiments on adult control testis to identify the mRNA directly bound to METTL16. They also performed IP on P10 testis to identify METTL16 protein partners, and detected MEIOC/YTHDC2/RBM46 among their partners.

The topic of the study is very interesting and timely, and the quantity and quality of the data presented is impressive. The figures and text are clear. However, I have some major concerns about the interpretation of a large part of the results. Besides some essential information is missing in the M&M section, which prevents me from reviewing the article satisfactorily in its present form.

My main concerns are:

1) Including immunofluorescence (IF) data showing METTL16 pattern of expression and localization in testicular sections would be useful. It would also be relevant to detect METTL16 in testicular sections from KO males to determine in which cell types/at which stage(s) METTL16 protein is no more visible. If METTL16 antibody does not work by IF, it could be addressed by performing western blot on testes collected before 10 days, or on purified undifferentiated and differentiated spermatogonia.

2) One major caveat of the present study is that RNA-seq, ribo seq and m6A-seq analyses were performed on P10 whole testes rather than on purified spermatogonia (or alternatively, on P8 testes). At P10 stage, the authors clearly show that the cell composition of KO and control testes is very different, since KO testes lack differentiated spermatogonia and early meiotic cells. It is therefore impossible to conclude that differentially expressed genes (or alternatively spliced, or genes with altered translation efficiency, etc.) are due to METTL16 loss; these transcriptome/translatome/m6A methylation changes certainly reflect (at least in part) the differences in the transcriptome/translatome programs of differentiated spermatogonia/early meiotic cells and other testicular cells. I therefore think that paragraph titles such as "METTL16 deletion alters expression of meiosis-related genes" or "METTL16 regulates the translation efficiency of genes in the testes" are not fully supported by the present data. The same applies for the article's title, and for the discussion.
Figure 4 shows the validation of several DEG, but in the text it is not indicated the material on which these experiments were performed… on whole testis or on purified kit+ cells ? If they were performed on kit+ cells, they are relevant data and it would also be useful to know details of gene normalization (one or several "house keeping" genes?), the number of genes tested, and if a correction for multiple tests was included.

3) The part in which the authors used purified c-KIT-positive spermatogonia from P8 to validate the alternative splicing of Stag3 and Stra8 by RT-qPCR is more convincing. However, it is unfortunate that this experiment appears to only have been carried out on two samples. It is therefore not appropriate to show statistics for this experiment (figure 4G).

1) The authors did not find the RNA of these two genes (Stag3 or Stra8) among the RNAs identified by RIP-Seq, according to Table S5. Yet it is written line 295 that "These results confirmed that METTL16 binds directly to Stra8 and Stag3 mRNA in P10 testes, as shown by RIP-seq data ". Can the authors explain? How can this discrepancy be interpreted?

2) The number of replicates for the Ribo-seq, RIP-seq (RIP-qPCR) and for the IP-MS experiments is not clearly indicated. The downstream bioinfo and statistical analyses are missing.

3) "The IP-MS data is provided in Supplementary Table 12." But this table was not provided. On figure 7A please also provide the number of METTL16 peptides identified.

4) In general, the M&M does not provide enough information for most of the experiments. This is particularly true for bioinformatic (including statistical) analyses which need to be presented for all experiments, and with more details, as part of the FAIR principle.

5) LC-ESI-MS/MS analysis of m6A levels: In the M&M section it is indicated that the experiment was performed on P10 testis, while in the text and in the figure legend it is indicated that it was performed on P8 whole testis.

6) In the M&M RNA-seq and RT-qPCR section it is only indicated that RNA was extracted from mouse testis (or from ckit+ spermatogonia) from P8 males, while RNA-seq were performed on whole testis from P10 stage. Please correct.

1) Discussion: line 431 "Previous research has shown that germ cell-specific deletion of m6A writers, either Mettl3 or Mettl14 mediated by Stra8-Cre, does not affect spermatogenesis, whereas simultaneous knockout of Mettl3 and Mettl14 causes defective spermiogenesis (Lin et al., 2017),". This is not an accurate statement since Mettl3 or Mettl14 KO (with Vasa-Cre) causes depletion of spermatogonial stem cells, and therefore a very severe spermatogenesis defect.

Minor comments

1) For non specialized readers, Stra8cre time of expression is unknown, please explain in the text (line 90). It will also be useful to have this information in the text (in addition to FigS2B) when describing Mettl16 conditional knockout model.

2) To convince the audience that the KO completely abolishes METTL16 protein level, please add more details (what would be the size of a truncated protein?) and/or provide a full length WB of Fig1E.

3) The evolution of METTL16 sequence has already been studied (at least partially) by Zhao et al, please cite PMID: 37085020

4) Line 90: "via stimulated by Stra8-Cre in mouse testes resulted in normal meiosis but m6A levels remain high in spermatocytes (Lin et al., 2017), suggesting that other
m6A methyltransferase members might exist to regulate m6A during meiosis."
Please correct typo and reformulate the sentence. Why using "but" ? the fact that m6A level remains high in spermatocytes and that Stra8-cre KO does not lead to meiotic defects are not opposite ideas. NB. It would be fair and useful to also mention the postmeiotic phenotypes (and m6a level in spermatids) of this mouse model…
5) Line 215 "Because the testes of Mettl16 cKO mice showed meiotic defects and no post-meiotic spermatozoa were present, we wanted to determine the stage at which meiosis was blockage." . What meiotic defects are the authors referring to? The previous paragraph present spermatogonial differentiation defects

6) Line 123: Stra8 and STAG3 ? gene or protein? Please change for consistency
7) Fig1C: typo in "spermatognonia"

**Reviewer 4**

**Were you able to assess all statistics in the manuscript, including the appropriateness of statistical tests used?**

Yes, and I have assessed the statistics in my report.

**Comments to author:**

Ma and Gui et al. presented a compelling study on the role of METTL16 in murine spermatogenesis. Their findings shed light on the mechanistic influence of METTL16 on alternative splicing of key transcripts related to meiosis. Overall, this study delves into an intriguing topic, executed with precision, and holds significance for both the epitranscriptomics and developmental biology fields. However, publication should be considered pending the resolution of several concerns. The primary issue revolves around the weak and correlative link between the observed phenotype and METTL16 m6A methyltransferase activity, despite the authors' attempt to establish causality.

Here are the major concerns:

* The manuscript consistently speculates, especially in Lines 371-375, 440, 452, and 450-451, that METTL16 m6A methyltransferase activity is pivotal for normal spermatogenesis in mice. However, no experiments supporting this claim have been conducted. Furthermore, the authors haven't investigated potential changes in the protein levels of other m6A writers, notably METTL3 and METTL14, or readers and erasers following METTL16 KO, which could potentially explain alterations in m6A methylation levels of specific transcripts in METTL16 KO tissues. The authors should either substantiate their claim or revise the text to consider the possibility that other components within the m6A pathway might be responsible for the observed changes.

* Related to the previous point, in Lines 393-396, the authors assert that "Taken together, these data demonstrate that METTL16 plays a critical role in spermatogenesis via its m6A transferase activity, regulating m6A modifications of METTL16 bound genes involved in germ cell development". This assertion lacks evidence supporting the importance of METTL16 methyltransferase activity in determining m6A levels of METTL16-bound transcripts. These transcripts might be methylated by METTL3/14, and changes in their m6A levels could result from misregulation of METTL3/14 or m6A erasers in METTL16 cKO testes. To validate their claim, the authors should demonstrate the restoration of m6A modification levels on METTL16-bound genes involved in germ cell development following the phenotype rescue by wild-type and mutant METTL16 injections in testes. This should be accompanied by evidence indicating the absence of changes in protein levels of other components in the m6A pathway.

* Regarding Fig 4H: RIP-qPCR enrichment should be calculated concerning the Input, not a negative control (IgG).

* In relation to Figures 4F, 5B, and the corresponding lines (lines 279 - 282, lines 315-317), where the authors note overlaps between differentially expressed genes (DEGs) and alternative splicing (AS) events, there's a suggestion to perform differential expression and translation analysis at the transcript level rather than the gene level. This approach might enhance the overlap between DEGs and AS events. Additionally, exploring the impact of altered alternative splicing on transcript translation could be insightful.

* Concerning the motif analysis shown in Fig 5H and 7F, it's unclear if both analyses were conducted using the same dataset. Moreover, there's inconsistency between the binding sites indicated in Fig 7B and the top METTL16 binding peaks in Fig 5H. The methodology for the RIP-seq data analysis and the identification of METTL16 binding sites/peaks requires clarification.

* The experiment illustrated in Fig 7C is not a Co-IP. Authors should conduct IP using antibodies against MEIOC, YTHDC2, and RBM46, and perform Western blotting using METTL16 antibodies.

* The purpose of the LC-ESI-MS/MS analysis of m6A levels performed by the authors is unclear. Analyzing total RNAs without rRNA depletion or polyA selection may not accurately reflect m6A levels in non-rRNA. Authors should explicitly state the aim of this analysis and the specific RNA subset they intended to examine.

* The authors should specify the number of biological and/or technical replicates used in all experiments, along with the number of animals per replicate.

* Additionally, the Methods and Materials section requires details on the analysis of Immunoprecipitation-mass spectrometry (IP-MS) data, and the authors should consider uploading the data to a public repository for transparency.

Minor points for consideration:

* Line 79: Clarify that METTL16 is an m6A methyltransferase for improved clarity. If discussing all vertebrate m6A methyltransferases, consider including METTL5 and ZCCHC4 or specify the discussion to non-rRNA m6A methyltransferases.

* In Figure 4A, specify on the plot that adjusted p-values are shown.

* In Figure 5E, include a legend indicating that the colors represent fold-change.

* In Lines 336-338, where the authors refer to RNA abundance but plot fold change, please elaborate for clarity.

* In Line 448-449, provide further clarification regarding the statement "the mRNA level was too low to be translated," potentially including references to support this phenomenon.

* In Lines 740-741, specify the parameters used to define "peaks."

**Authors Response**

**Point-by-point responses to the reviewers’ comments:**

REVIEWER REPORTS:

Reviewer #1: In this manuscript, the authors suggest a molecular model wherein METTL16-mediated alternative splicing and translation regulation play a crucial role in determining the mitosis-to-meiosis transition in germ cell fate in mice. This paper, well written and have sufficient details, is of outstanding interest to a broad audience of biologists. While the study offers valuable insights, I have identified both major and minor concerns that, if addressed, could further enhance the quality and clarity of this research.

***RESPONSE:*** *We thank you for your careful scrutiny of our work and appreciate your suggestions for improving the rigor and quality of our study. We have endeavored to provide more convincing evidence in the revision to further clarify our research.*

Regarding splicing regulation by METTL16:

- There is a lack of figures illustrating alternative splicing changes for Mat2a (Line 273) following the loss of METTL16. Existing studies highlight METTL16's role in regulating MAT2A splicing in C. elegans (Mendel et al., Cell 2021) and human cells (Pendleton et al., Cell 2017) via either its methyltransferase or non-methyltransferase activities. Furthermore, I am wondering this Mat2a splicing changes in mice are due to METTL16's catalytic function or not?

***RESPONSE:*** *Thank you for asking a good question. In the current study, we focused on identifying the alternative 3' splice site (A3SS) splicing event of Mat2a from our RNA-seq data, which differs from that reported in C. elegans and human cells. In addition, we found that the expression level of Mat2a mRNA was not altered in P10 Mettl16 cKO mouse testes compared to controls (see new Supplementary* ***Figure S5C****), suggesting that the spermatogenesis arrest in Mettl16 cKO mice may not directly correlated with the A3SS splicing of Mat2a in mice. Therefore, these changes in Mat2a splicing (A3SS) observed in mice may not be due to the catalytic function of METTL16. We have mentioned this possibility in the revised manuscript (see lines 494-500 with yellow highlighted).*

- In Line 277, the suggestion that METTL16 may repress alternative splicing in the testes warrants clarification. How many splicing changes correlate with METTL16 binding or m6A peaks?

***RESPONSE:*** *Thank you for your question. We redone METTL16 RIP-seq using isolated P10 spermatogenic cells in the revision and found that 459 genes with AS events changed correlated with METTL16 binding and 874 AS events changed genes associated with m6A peaks. We have added these new data in the revised manuscript (see new* ***Fig.6F*** *and lines 373-375 with yellow highlighted).*

- U6 snRNA, a known METTL16 target essential for splicing, influences the splicing of specific 5' splice sites in yeast and plant studies (Ishigami et al., Nat Commun 2021; Parker et al., eLife 2022) but remains unexplored in mammalian cells. One of the interesting questions is that whether this 5' splice sites bias are also shown in mammalian cells. The author's proposal of METTL16 interaction with splicing regulator SF3B1/3 raises questions regarding splicing changes attributed to METTL16/U6 versus METTL16/SF3B. Utilizing established cell lines expressing catalytic mutant METTL16 could help differentiate splicing changes influenced by its catalytic versus non-catalytic functions.

***RESPONSE:*** *Thank you for raising this interesting question. We indeed identified 322 alternative 5’ splice site (A5SS) events in P10 Mettl16 cKO testis as shown in* ***Fig. 4E****, suggesting that the 5' splice site bias also occurred in mammalian cells.*

*In addition, we agree with your suggestion that in vitro cell lines expressing catalytic mutant METTL16 could help differentiate alternative splicing influenced by its catalytic or non-catalytic functions. We thus redesigned the minigene pcDNA3.1-Ddb2 and co-transfected it with METTL16 wild-type or catalytic mutant vectors in 293T cells. The results showed that in vitro splicing regulation was different from that in vivo, and the METTL16 catalytic mutant seemed to work as well as the METTL16 wild-type. We have added the new data in Supplementary* ***Figure S5D-E.***

Regarding translation regulation by METTL16:

- In Line 308, the authors conclude that the translation efficiency of METTL16 targets was lower than non-targets in METTL16-deficient testes. In the above paragraph, they showed that the direct targets of METTL16, Stra8 and Meioc, are translated more in METTL16-deficient testes though their mRNA abudance become lower. The conclusion and their examples appear to be contradictory.

***RESPONSE:*** *Thank you for pointing out this issue. We have reperformed METTL16 RIP-seq experiments using isolated P10 spermatogenic cells and re-analyzed the translation efficiency of METTL16 targets and non-targets. Interestingly, we still found the translation efficiency (TE) of METTL16 targets was lower than non-targets in P10 METTL16-deficient spermatogenic cells (see new* ***Fig.5E****). However, the question you raised also caught our attention, and we divided the analyzed genes into an up-regulated group and a down-regulated group for further analysis. The new results showed that, for down-regulated genes (such as Stra8 and Meioc), the translation efficiency of METTL16 targets was higher than non-targets in METTL16-deficient spermatogenic cells (see new* *Supplementary* ***Figure S4G-H****), which is consistent with the example results shown in new Supplementary* ***Figure S4F*** *as well.*

- Via biochemistry experiments, the authors found that METTL16 interacts with MEIOC/YTHDC2/RBM46. However, the evidence for this interaction functionally regulating transcription and translational is lacking in this manuscript.

***RESPONSE:*** *Thank you for your comment. We agree with the importance of functional interaction among METTL16 and MEIOC/YTHDC2/RBM46 at the transcriptional and translational levels, but this seems to be beyond the scope of the current research. Based on the present study, it is clear that in P10 testis, METTL16 interacts with RNA close to MEIOC/YTHDC2/RBM46 binding sites and that METTL16 deletion can lead to decreased expression levels of YTHDC2 and MEIOC but does not affect the protein level of RBM46. We will follow up on the study for this interaction functionally regulating transcription and translational among these complexes in the future.*

Meanwhile, I have several minor points that I hope can be helpful for this study.

- The authors observed that m6A-sequencing showed that ablation of METTL16 caused upregulation of the m6A-enriched transcripts and downregulation of the m6A-depleted transcripts. However, the authors also observed that METTL16 knockout causes very minor global m6A changes (by MS and m6A-seq). (1) Can the authors plot m6A levels on U6 RNA with and without METTL16? This could be used as a proof-of-concept. (2) As the majority of m6A on mRNA are not installed by METTL16 and they stayed unchanged with and without METTL16, how does the expression level of m6A-enriched transcripts are upregulated whilst m6A-depleted transcripts are downregulated due to loss of METTL16?

***RESPONSE:*** *Thank you for raising these comments and good suggestions. (1) We have tried to plot m6A levels on U6 RNA, but unfortunately, we have failed because our m6A-seq data mainly detects the m6A modifications on mRNA, not on small nuclear RNA. (2) Following your comment, we have used P8 Mettl16 cKO testis to detect the expression level of other classic m6A installed proteins, including METTL3, METTL14, ALKBH5, and YTHDF2. The results showed that the expression levels of these proteins did not change in the P8 Mettl16 cKO testis compared to that of controls (see* *new* ***Figure 5G-H****). Nevertheless, our m6A-seq data still detected many m6A differentially modified genes, suggesting there may be potential m6A-installed pathways regulated by METTL16 directly that require elucidation in the future.*

- In Figure 7D, why is METTL16 protein still present in the Mettl16 cKO samples by WB?

***RESPONSE:*** *Thank you for your question. Since METTl16 is ubiquitously expressed in all types of testis cells, in the current study, we used Stra8-Cre to specifically knock out Mettl16 in germ cells (from type A1 spermatogonia). In* ***Figure 7D****, we used whole P10 testes containing somatic cells (e.g., Sertoli cells) to* *determine the protein level of METLL16, which led to a weak METTL16 protein band observed in Mettl16 cKO samples by WB.*

- What's the rational for global m6A level quantification of Mettl16 cKO using total RNA and mass spectrometry, rather than poly(A)+ RNA, rather than poly(A)- RNA? I believe that the authors are clear that rRNA is dominant population (>90%) of total RNA, the m6A on which are installed by other methyltransferases.

***RESPONSE:*** *Thank you for your expert comment. Indeed, we also considered that rRNA accounted for a high proportion of total RNA, but since METTL16 was initially reported to mainly catalyze non-coding RNA and one mRNA (Mat2a) (Pendleton et al., 2017), we chose total RNA for mass spectrometry. In addition, as a recent study showed that METTL16 can catalyze hundreds of mRNAs (Su et al., 2022), we performed m6A-seq to detect the m6A modification on mRNAs in the current study. We also mentioned this rationale in the main text (see lines 336-344 with yellow highlighted).*

- It's unclear what does "but failed" mean in Line 329.

***RESPONSE:*** *We originally wanted to express that even the translation efficiency of several important genes, such as Stra8 and Meioc, was upregulated in Mettl16 cKO testes, but the protein levels of these genes failed to upregulated but ultimately downregulated. We removed “but failed” in the revision to avoid misunderstanding.*

- I would suggest the authors plot their m6A enrichment, shown in figure 6D, in a manner consistent with literature using metaPlotR (https://github.com/olarerin/metaPlotR) or RNAmpp (https://github.com/guifengwei/RNAmpp).

***RESPONSE:*** *Done as suggested. Thank you.*

Reviewer #2: Qian Ma et al. have generated testes- specific conditional Mettl16 KO mice to investigate its role in spermatogenesis. They have performed an in-depth analysis of the testes in these mice, including histochemical analyses, immunoblotting, RNAseq, RIP-seq, meRIP-seq and determined the translation efficiency by ribosome profiling.

Overall, they obtained some very interesting results suggesting a role of Mettl16 in the progression of meiosis and alternative splicing, the latter of which had been described before in different systems. Rescue experiments suggested that the catalytic activity of Mettl16 was required for its function in spermatogenesis. However, Mettl16-specific m6A sites could not be linked to alternatively spliced potential target transcripts, so overall it is not quite clear how Mettl16 activity mechanistically relates to the multiple observations described by the authors (binding of splicing factors, alternative splicing of several transcripts, interaction MEIOC/RBM46/YTHDC2 complex, differential methylation of select transcripts,…).

***RESPONSE:*** *Thank you very much for your appreciation of our interesting results about the role of Mettl16 and your valuable comments, which enabled us to improve the quality of the manuscript. We have performed additional experiments and analyses to solidify the conclusions according to the suggestions. We hope you will be satisfied with our revision and find it suitable for publication.*

Major points:

Line 116: the authors should mention that a germ-cell specific conditional KO mouse model was generated and described in Mendel et al. 2021, also describing infertility, high Mettl16 expression in testes and decreased testes size. They should discuss the advantages or additional information yielded by analyzing Stra8-Cre conditional KO mice.

***RESPONSE:*** *We appreciate these good suggestions. We have revised our manuscript as suggested (see lines 100-105 in Background and lines 429-434 in Discussion).*

The quality of the immunoblot in Fig.1A is nor adequate. Please provide a better blot image. Please provide the age of the mice used for immunoblotting in the figure legend.

***RESPONSE:*** *As the specificity of the METTL16 antibody was not very good, we could not obtain a perfect blot image despite several attempts to perform WB. In the revision, we have provided a slightly better blot image of* ***Figure 1A*** *as far as possible. We hope that you will be satisfied with this new blot image. Thank you very much for your careful review.*

Figure S2F please provide more representative images of PLZF+ cells in tubule sections.

***RESPONSE:*** *We have used arrows to indicate the representative PLZF^+^ cells in the seminiferous tubule sections of new Supplementary* ***Figure S2F****, as suggested.*

Please provide a more detailed description of the results and legend presented in Fig. 3G. The description in the legend seems to refer to the cKO only.

***RESPONSE:*** *We apologize for this oversight. We added a more detailed description of the control results about* ***Figure 3G*** *(see lines 216-220 with yellow highlighted).*

Fig. 6I Please provide WT control as comparison for the partial rescue.

***RESPONSE:*** *Done as suggested. Now, it was shown in revised* ***Figure 6H****. Thanks!*

Fig. 7C The IP bands for MEIOC (unspecific IgG binding and immunoprecipitated by anti-Mettl16 antibodies) seem to display a different electrophoretic mobility. How do the authors explain this difference?

***RESPONSE:*** *Thank you for pointing out this issue, which may be caused by inconsistent pH between the input and the IP samples during the experimental process because previous studies have shown that the mobility of several proteins changed with changing pH values (Pusztai and Watt, 1971). When preparing the input sample, we added the appropriate 5x loading buffer to the protein lysis buffer and made it ‘1x’. And when preparing the IP samples, we directly added 1x loading buffer to the magnetic beads with binding proteins. Due to the different sample preparation, the pH values in the samples could change.*

Please provide immunoblots of a non-binding protein (e.g. a YTH-domain containing protein not identified in the interactome) as control for the specificity of the co-IP.

***RESPONSE:*** *In the revision, we used RPL19 as non-binding protein control for the specificity of the coIP assays (see new* ***Figure 7C****).*

To better dissect which of the observed phenotypes are causal for the physiological consequences which are not rescued by catalytically inactive Mettl16, the authors should perform select experiments (e.g. RT-PCR for alternative splicing of Stra8, Stag3) in the cKO testes injected with AAV expressing WT or PP185-186AA Mettl16.

***RESPONSE:*** *We appreciate that you have raised this critical comment, which motivated us to substantially expand our investigations in vivo and in vitro. Since we observed abnormal spermatogonia differentiation and meiotic initiation phenotypes in Mettl16 cKO mice, we then decided to perform rescue experiments using P5 mice for efferent duct injection of AAV expressing WT or PP185-186AA Mettl16. However, we were unable to anesthetize the newborn mice (at P5 age) to inject the plasmid into their efferent duct. In addition, the newly generated METTL16^GFP^-tagged mice also showed high METTL16 expression in non-germ cells (such as Sertoli cells) as well (see new Supplementary* ***Figure S1D****), which indicating a direct testicular injection of AAV in vivo was also not advisable. Therefore, we sought to perform an alternative experiment in vitro to dissect the cause of aberrant alternative splicing events.*

*We designed minigenes of interested splicing genes and co-transfected them into 293T cells with*

*METTL16 wild-type or catalytic mutant vectors. Initially, we planned to design minigene of Stag3 and Stra8, but it turned out that the number of intron bases of Stra8 was too long to clone it into an overexpression vector. We then engineered minigenes of Stag3 and Ddb2 (splicing changed in Mettl16 cKO spermatogonia) (see new* ***Figure 4G****) and transfected them into cells with either wildtype or mutant METTL16. Surprisingly, Stag3 did not undergo alternative splicing in 293T cells, and Dbd2 did undergo alternative splicing but the splicing pattern in 293T cells was different from that in vivo (see new* *Supplementary* ***Figure S5E****).*

In general, the authors should provide more detailed method descriptions (e.g. for m6A-seq, ribosome profiling, MS) or cite publications adequately describing the methods the authors employed.

***RESPONSE:*** *We have provided more detailed method descriptions in the Methods section (see highlighted text). Thank you again for your careful review.*

Minor points:

Lines 141 to 148. The relevance and novelty of this paragraph in the beginning of the results section (including supplementary figure 1A/B) is unclear to this reviewer. Please integrate into a paragraph where the alignment and high degree of conservation strengthens the presented results.

***RESPONSE:*** *Thank you for your comments. We have rewritten this paragraph according to your suggestion (see lines 126-131 with yellow highlighted).*

Line 217: ..was blocked…

***RESPONSE:*** *Corrected. Thank you!*

Reviewer #3: Mettl16 encodes an evolutionary conserved m6A methyltransferase which is ubiquitously expressed. It has been shown to catalyze m6A methylation of U6 snRNA and of the SAdenosylmethionine (SAM) synthetase MAT2A mRNA. Its role during spermatogenesis, a process in which transcriptomic complexity is high and post-transcriptional regulation is very dynamic, has not yet been addressed.

In their manuscript (GBIO-D-23-01720) Ma et al. presents the phenotypic and molecular consequences of the conditional knockout of Mettl16 in postnatal male germ cells. The authors used Stra8-Cre (as part of the CRE/LOX system), a construct known to express the Cre recombinase in spermatogonia from postnatal day3. They first show that KO males are infertile due to a premeiotic defect in the differentiation of spermatogonia. As a result, KO testes from day 10 show a striking loss of germ cells compared to testes from control males of the same age. The number of differentiating (c-kit+) spermatogonia is severely reduced, and almost no meiotic cells are detected (while at this stage, preleptotene meiotic cells are normally present). The authors conclude that spermatogonia differentiation and meiotic initiation are impaired by Mettl16 KO.

The authors then performed RNA-seq and ribo-seq on P10 whole testis from KO vs control males and concluded that METTL16 deletion alters expression of meiosis-related genes, with the identification of >1000 differentially transcribed genes and >1000 genes with altered translation efficiency. They also investigate alternative splicing using rMATS tool and identified many alternative splicing events between KO and CTL testis. They compared their data with P8 testes m6A-IP libraries, and also performed m6A-seq in P10 testes from KO and CTL. Performing "rescue" experiment they show that m6A methyltransferase activity of METTL16 is necessary for its function during spermatogenesis. Finally, the authors conducted RIP-seq experiments on adult control testis to identify the mRNA directly bound to METTL16. They also performed IP on P10 testis to identify METTL16 protein partners, and detected MEIOC/YTHDC2/RBM46 among their partners.

The topic of the study is very interesting and timely, and the quantity and quality of the data presented is impressive. The figures and text are clear. However, I have some major concerns about the interpretation of a large part of the results. Besides some essential information is missing in the M&M section, which prevents me from reviewing the article satisfactorily in its present form.

***RESPONSE:*** *Thank you for your appreciation of our interesting findings. We would also like to thank you for your valuable comments, which significantly improved the quality of our manuscript.*

My main concerns are:

1) Including immunofluorescence (IF) data showing METTL16 pattern of expression and localization in testicular sections would be useful. It would also be relevant to detect METTL16 in testicular sections from KO males to determine in which cell types/at which stage(s) METTL16 protein is no more visible. If METTL16 antibody does not work by IF, it could be addressed by performing western blot on testes collected before 10 days, or on purified undifferentiated and differentiated spermatogonia.

***RESPONSE:*** *Thanks for your constructive suggestions. We agree that immunofluorescence (IF) is the better method to show the METTL16 expression pattern and determine the METTL16 KO efficiency in testicular cells. However, all the available commercial METTL16 antibodies did not work by IF. Therefore, we have generated METTL16^GFP^-tagged mice via a knock-in strategy during the manuscript submission stage. IF staining of Tagged mouse testicular sections using GFP antibody revealed that METTL16 was uniquely expressed in the P14 testis, with more intense signals observed in WT1^+^ Sertoli cells (see new Supplementary* ***Figure S1D****), c-KIT^+^ spermatogonia (see new* ***Figure 1D****), early spermatocytes with dot-like SYCP3, and weak signals in pachytene spermatocytes of P14 testes. GFP signals were also found in PLZF^+^ undifferentiated spermatogonia of adult testes (see new Supplementary* ***Figure S1D****).*

*Since the commercial METTL16 antibody did not work for IF, we followed your suggestion and performed a Western blot on P8 testes. We found that METTL16 protein levels were significantly reduced in Mettl16 cKO testes compared to control testes (see new* *Supplementary* ***Figure S2C****), further suggesting that METTL16 has been successfully depleted in the mouse spermatogonial cell type.*

2) One major caveat of the present study is that RNA-seq, ribo seq and m6A-seq analyses were performed on P10 whole testes rather than on purified spermatogonia (or alternatively, on P8 testes). At P10 stage, the authors clearly show that the cell composition of KO and control testes is very different, since KO testes lack differentiated spermatogonia and early meiotic cells. It is therefore impossible to conclude that differentially expressed genes (or alternatively spliced, or genes with altered translation efficiency, etc.) are due to METTL16 loss; these transcriptome/translatome/m6A methylation changes certainly reflect (at least in part) the differences in the transcriptome/translatome programs of differentiated spermatogonia/early meiotic cells and other testicular cells. I therefore think that paragraph titles such as "METTL16 deletion alters expression of meiosis-related genes" or "METTL16 regulates the translation efficiency of genes in the testes" are not fully supported by the present data. The same applies for the article's title, and for the discussion.

***RESPONSE:*** *Thank you for pointing out this issue. We agree with your comment that it would be better to use purified spermatogonia rather than whole testes for the sequencing analyses. In fact, we have tried to purify the spermatogonia using P8-10 testes multiple times, but unfortunately, we technically failed to collect enough cells for sequencing (ribo-seq, m6A-seq) due to the limited Mettl16 cKO testes available during each cell purification process. To avoid delaying the publication of the current study, we alternatively selected whole testes to perform sequencing analyses.*

*As shown in* ***Figure 2C****, the number of c-KIT positive spermatogonia present in P10 cKO testes was about 50% of that in control mice. During chromosome spread analysis, early meiotic cells, including SYCP3^+^γH2AX^+^ pre-leptotene like spermatocytes and SYCP3-γH2AX^+^ germ cells, were found in P10 Mettl16 cKO testes (****Figure 3G****). These detailed analyses confirmed the presence of differentiated spermatogonia and early meiotic cells in P10 Mettl16 cKO testes. Therefore, we decided to use the P10 whole testes at which spermatogonial differentiation and meiosis initiation have been completed.*

*Although the number of DDX4-positive germ cells and c-KIT-positive spermatogonia was reduced in P10 Mettl16 cKO mice, c-KIT-positive spermatogonia and preleptotene spermatocytes were still present in P10 cKO testes, which might reflect the differences in transcriptome/translatome programs of differentiated spermatogonia/early meiotic cells to some extent by sequencing and does not affect the overall conclusion much. In addition, we have also validated the DEGs using purified c-KIT^+^ spermatogonia cells to confirm the reliability of the sequencing data. Thus, our multi-omics data confirmed that the altered expression of meiosis-related genes in P10 Mettl16 cKO testes was caused by METTL16 ablation rather than reduced numbers of differentiated spermatogonia and early meiotic cells, and the time point of P10 was appropriate for* *transcriptional analysis. We hope that this reviewer is satisfied with our explanation and understands our situation.*

Figure 4 shows the validation of several DEG, but in the text it is not indicated the material on which these experiments were performed… on whole testis or on purified kit+ cells ? If they were performed on kit+ cells, they are relevant data and it would also be useful to know details of gene normalization (one or several "house keeping" genes?), the number of genes tested, and if a correction for multiple tests was included.

***RESPONSE:*** *We apologize for this carelessness. Validation of DEGs was performed by RT-qPCR on purified c-KIT^+^ spermatogonial cells (see lines 241-243 with yellow highlighted). Actin was used as an internal control for gene expression normalization. Statistical analysis between normalized gene expression in each sample was performed by two-sided Student’s t-test using GraphPad Prism 8.0. We have included this information in the Figure legend of revised manuscript.*

3) The part in which the authors used purified c-KIT-positive spermatogonia from P8 to validate the alternative splicing of Stag3 and Stra8 by RT-qPCR is more convincing. However, it is unfortunate that this experiment appears to only have been carried out on two samples. It is therefore not appropriate to show statistics for this experiment (figure 4G).

***RESPONSE:*** *Thank you for your comment. In the revision, we have redone this experiment to validate the alternative splicing of Stag3 and Ddb2 by RT-qPCR in c-KIT-positive spermatogonia from P10 testes. Statistical analysis was carried out on three replicates.*

4) The authors did not find the RNA of these two genes (Stag3 or Stra8) among the RNAs identified by RIP-Seq, according to Table S5. Yet it is written line 295 that "These results confirmed that METTL16 binds directly to Stra8 and Stag3 mRNA in P10 testes, as shown by RIP-seq data ". Can the authors explain? How can this discrepancy be interpreted?

***RESPONSE:*** *Thank you for your careful review. We actually performed RIP-seq on both adult and P10 mouse isolated spermatogenic cells, and interestingly, we found that the targeted genes, Stag3 and Stra8, were present in the P10 RIP-seq data but not in the adult RIP-seq data. We have replaced Table S5 with P10 RIP-seq data in the revision.*

5) The number of replicates for the Ribo-seq, RIP-seq (RIP-qPCR) and for the IP-MS experiments is not clearly indicated. The downstream bioinfo and statistical analyses are missing.

***RESPONSE:*** *We apologize for this unclear indication. The minimum amount of RNA used for ribo-seq was approximately 30 μg, and to avoid degradation, the testes should be sent for analysis within one month, which means we need to collect at least ten cKO P10 male mice in one month to run a replicate. We sent three replicates for analysis, but two failed due to degradation of cKO RNA. RIP-seq of adult and P10 testes was performed once each. RIP-qPCR was performed at least three times to validate the RIP-seq results. IP-MS experiments were also performed on P10 testes in two replicates. The downstream bioinformatic and statistical analyses have been added to the Methods section (see the highlighted text).*

6) "The IP-MS data is provided in Supplementary Table 12." But this table was not provided. On figure 7A please also provide the number of METTL16 peptides identified.

***RESPONSE:*** *The IP-MS data has been provided in Supplementary* ***Table S9****, and we have corrected the description accordingly. We identified 33 METTL16 peptides from IP-MS and have added this information in* ***Figure 7A****, as suggested.*

7) In general, the M&M does not provide enough information for most of the experiments. This is particularly true for bioinformatic (including statistical) analyses which need to be presented for all experiments, and with more details, as part of the FAIR principle.

***RESPONSE:*** *Thank you for pointing this out. We have provided the detailed information in the Methods section (see the highlighted text).*

8) LC-ESI-MS/MS analysis of m6A levels: In the M&M section it is indicated that the experiment was performed on P10 testis, while in the text and in the figure legend it is indicated that it was performed on P8 whole testis.

***RESPONSE:*** *We apologize for this carelessness and have corrected P10 in the Methods section to P8 (see line 779).*

9) In the M&M RNA-seq and RT-qPCR section it is only indicated that RNA was extracted from mouse testis (or from ckit+ spermatogonia) from P8 males, while RNA-seq were performed on whole testis from P10 stage. Please correct.

***RESPONSE:*** *In these cases, all samples were from P10 mosue testes or c-KIT^+^ spermatogonia. We have corrected them (see line 639). Thank you!*

10) Discussion: line 431 "Previous research has shown that germ cell-specific deletion of m6A writers, either Mettl3 or Mettl14 mediated by Stra8-Cre, does not affect spermatogenesis, whereas simultaneous knockout of Mettl3 and Mettl14 causes defective spermiogenesis (Lin et al., 2017),". This is not an accurate statement since Mettl3 or Mettl14 KO (with Vasa-Cre) causes depletion of spermatogonial stem cells, and therefore a very severe spermatogenesis defect.

***RESPONSE:*** *Agreed! We have rephrased this statement to make it more accurate (see lines 420-428 in the Discussion section).*

Minor comments

1) For non specialized readers, Stra8cre time of expression is unknown, please explain in the text (line 90). It will also be useful to have this information in the text (in addition to FigS2B) when describing Mettl16 conditional knockout model.

***RESPONSE:*** *Good suggestion. We have done as suggested.*

2) To convince the audience that the KO completely abolishes METTL16 protein level, please add more details (what would be the size of a truncated protein?) and/or provide a full length WB of Fig1E.

***RESPONSE:*** *As suggested, we have added more details regarding the generation of Mettl16 cKO mice in the methods of revised manuscript (see lines 553-562 with yellow highlighted). We also performed additional WB experiments using P8 testes from Control and Mettl16 cKO mice to further validate the METTL16 KO efficiency and provided a full-length WB image shown in Supplementary* ***Figure S2C****. Since METTl16 is ubiquitously expressed in all types of testicular cells, in the current study, we used Stra8-Cre to specifically knock out Mettl16 in germ cells (from type A1 spermatogonia), which led to a weak METTL16 protein band observed in Mettl16 cKO samples by WB.*

3) The evolution of METTL16 sequence has already been studied (at least partially) by Zhao et al, please cite PMID: 37085020

***RESPONSE:*** *We cited the reference as suggested.*

4) Line 90: "via stimulated by Stra8-Cre in mouse testes resulted in normal meiosis but m6A levels remain high in spermatocytes (Lin et al., 2017), suggesting that other m6A methyltransferase members might exist to regulate m6A during meiosis." Please correct typo and reformulate the sentence. Why using "but" ? the fact that m6A level remains high in spermatocytes and that Stra8-cre KO does not lead to meiotic defects are not opposite ideas. NB. It would be fair and useful to also mention the postmeiotic phenotypes (and m6a level in spermatids) of this mouse model…

***RESPONSE:*** *We have corrected this sentence as suggested (see lines 77-81). Thanks!*

5) Line 215 "Because the testes of Mettl16 cKO mice showed meiotic defects and no post-meiotic spermatozoa were present, we wanted to determine the stage at which meiosis was blockage." . What meiotic defects are the authors referring to? The previous paragraph present spermatogonial differentiation defects

***RESPONSE:*** *Thank you for your comment. We have rephrased this sentence to make the paragraph transition more smoothly (see lines 200-203).*

6) Line 123: Stra8 and STAG3 ? gene or protein? Please change for consistency

***RESPONSE:*** *Corrected. Thank you!*

7) Fig1C: typo in "spermatognonia"

***RESPONSE:*** *Corrected.*

Reviewer #4: Ma and Gui et al. presented a compelling study on the role of METTL16 in murine spermatogenesis. Their findings shed light on the mechanistic influence of METTL16 on alternative splicing of key transcripts related to meiosis. Overall, this study delves into an intriguing topic, executed with precision, and holds significance for both the epitranscriptomics and developmental biology fields. However, publication should be considered pending the resolution of several concerns. The primary issue revolves around the weak and correlative link between the observed phenotype and METTL16 m6A methyltransferase activity, despite the authors' attempt to establish causality.

***RESPONSE:*** *Thank you very much for your evaluation and insightful comments, which enabled us to improve the quality of the manuscript. We have thoroughly revised the manuscript according to your comments and hope you will be satisfied with our revision.*

Here are the major concerns:

- The manuscript consistently speculates, especially in Lines 371-375, 440, 452, and 450-451, that METTL16 m6A methyltransferase activity is pivotal for normal spermatogenesis in mice. However, no experiments supporting this claim have been conducted. Furthermore, the authors haven't investigated potential changes in the protein levels of other m6A writers, notably METTL3 and METTL14, or readers and erasers following METTL16 KO, which could potentially explain alterations in m6A methylation levels of specific transcripts in METTL16 KO tissues. The authors should either substantiate their claim or revise the text to consider the possibility that other components within the m6A pathway might be responsible for the observed changes.

***RESPONSE:*** *Thank you for your comments. As you suggested, we have investigated the expression of other m6A writers (METTL3, METTL14), reader YTHDF2 and eraser ALKBH5 in P8 testes by Western blot. Interestingly, the results showed that METTL16 deletion does not affect the protein levels of these classical m6A pathway proteins (new* *Supplementary* ***Figure 5G-H****), suggesting that the changes in m6A modifications observed in Mettl16 cKO mice are likely caused by METTL16 methyltransferase activity and that METTL16 may play a critical role in regulating m6A levels during spermatogenesis. We have included these new data in the revision and revised the text accordingly (see lines 344-350 with yellow highlighted).*

- Related to the previous point, in Lines 393-396, the authors assert that "Taken together, these data demonstrate that METTL16 plays a critical role in spermatogenesis via its m6A transferase activity, regulating m6A modifications of METTL16 bound genes involved in germ cell development".

This assertion lacks evidence supporting the importance of METTL16 methyltransferase activity in determining m6A levels of METTL16-bound transcripts. These transcripts might be methylated by METTL3/14, and changes in their m6A levels could result from misregulation of METTL3/14 or m6A erasers in METTL16 cKO testes. To validate their claim, the authors should demonstrate the restoration of m6A modification levels on METTL16-bound genes involved in germ cell development following the phenotype rescue by wild-type and mutant METTL16 injections in testes. This should be accompanied by evidence indicating the absence of changes in protein levels of other components in the m6A pathway.

***RESPONSE:*** *Thank you very much for your comments and suggestions. As responded to above, we have examined the protein levels of other components of the m6A pathway, including m6A writers (METTL3, METTL14), reader YTHDF2 and eraser ALKBH5 in P8 testes from control and Mettl16 cKO mice, and found that the expression levels of all these classical m6A-related proteins are not affected in Mettl16 cKO testes. We have also tried to detect the m6A levels of METTL16-bound genes using the rescued testis samples, but were unsuccessful because only a few seminiferous tubules could be rescued, and the number of successfully rescued tubules was too small to perform subsequent detection. However, we have carefully revised our assertion to avoid overstatement in the conclusion (see lines 387-389).*

- Regarding Fig 4H: RIP-qPCR enrichment should be calculated concerning the Input, not a negative control (IgG).

***RESPONSE:*** *Done as suggested. Thank you for your careful review.*

- In relation to Figures 4F, 5B, and the corresponding lines (lines 279 - 282, lines 315-317), where the authors note overlaps between differentially expressed genes (DEGs) and alternative splicing (AS) events, there's a suggestion to perform differential expression and translation analysis at the transcript level rather than the gene level. This approach might enhance the overlap between DEGs and AS events. Additionally, exploring the impact of altered alternative splicing on transcript translation could be insightful.

***RESPONSE:*** *Thank you for your comment and good suggestion. We agree with your comment that it would indeed be more meaningful to analyze differential expression and translation regulation at the level of a single transcript. Alternative splicing can cause a gene to produce multiple mRNA transcripts, resulting in one mRNA being translated into different proteins. By analyzing alternative splicing or translation at the gene level, one could get an overall result of multiple transcripts stacked on top of each other. In addition, in cKO testes, the regions identified as undergoing alternative splicing may also belong to transcripts A, B, C... and different transcripts correspond to different expression and translation regulations. We, therefore, decided to analyze the overlap between DEGs and AS events at the gene level.*

*Additionally, following your suggestion, we preliminarily explored the relationship between the occurrence of alternative splicing genes and the occurrence of translation regulatory genes. Combined bioinformatic analysis revealed that 85 genes were simultaneously under both splicing and translation regulation (see Supplementary* ***Figure S4B****). Interestingly, further analysis of these 85 genes revealed that their functions are mainly enriched in some basic biological processes and have little to do with phenotype (see Supplementary* ***Figure S4C****). This suggests that the translational regulation of alternatively spliced genes in Mettl16 cKO mice may not be directly related to their phenotype, but this does not mean that altered splicing does not affect translation. We have added the new data and revised the text in the revision (see lines 294-299 with yellow highlighted).*

- Concerning the motif analysis shown in Fig 5H and 7F, it's unclear if both analyses were conducted using the same dataset. Moreover, there's inconsistency between the binding sites indicated in Fig 7B and the top METTL16 binding peaks in Fig 5H. The methodology for the RIP-seq data analysis and the identification of METTL16 binding sites/peaks requires clarification.

***RESPONSE:*** *Thank you for pointing this out. We have conducted the motif analysis for Fig.5H and Fig.7F using the same RIP-seq dataset (P10 testes). Methodologically, we used Homer (version 4.10) to identify sequence motifs enriched in peak regions and obtained many potential motifs from the RIP-seq data. In Fig.5H, we wanted to show the top three motifs, while in* ***Figure 7F****, we just showed the motif we selected from many* *potential motifs that may bind to the same regions as YTHDC2/MEIOC/RBM46. We have provided the detailed RIP-seq data analysis in the Methods section (see the lines 588-701 with highlighted).*

- The experiment illustrated in Fig 7C is not a Co-IP. Authors should conduct IP using antibodies against MEIOC, YTHDC2, and RBM46, and perform Western blotting using METTL16 antibodies.

***RESPONSE:*** *We agree with this suggestion and have performed additional Co-IP assays as suggested. The new IP results are shown in Supplementary* ***Figure S5B.***

- The purpose of the LC-ESI-MS/MS analysis of m6A levels performed by the authors is unclear. Analyzing total RNAs without rRNA depletion or polyA selection may not accurately reflect m6A levels in non-rRNA. Authors should explicitly state the aim of this analysis and the specific RNA subset they intended to examine.

***RESPONSE:*** *Thank you for your comment. We agree with you that analyzing the m6A levels of polyA mRNA is more useful than analyzing total RNA by LC-ESI-MS/MS. Considering that the substrates of METTL16 included both non-coding RNAs and hundreds of mRNAs, we chose total RNA to detect global m6A levels and explore the global change of RNA upon METTL16 deletion. Interestingly, no significant changes in the overall m6A levels were observed in METTL16-deficient and control testes; however, we have mentioned the possible effect of rRNA on m6A levels in the revised manuscript to avoid any misunderstanding of our data (see lines 339-344 with yellow highlighted).*

- The authors should specify the number of biological and/or technical replicates used in all experiments, along with the number of animals per replicate.

***RESPONSE:*** *We have added the number of replicates and the number of animals per replicate to the Figure legend / Methods section.*

- Additionally, the Methods and Materials section requires details on the analysis of Immunoprecipitation-mass spectrometry (IP-MS) data, and the authors should consider uploading the data to a public repository for transparency.

***RESPONSE:*** *Details of the analysis of the IP-MS data are provided in the Methods section (see lines 711-719 with yellow highlighted), and the IP-MS data have been uploaded to a separate Supplementary* ***Table S9*** *for transparency. Thank you for your careful review.*

Minor points for consideration:

- Line 79: Clarify that METTL16 is an m6A methyltransferase for improved clarity. If discussing all vertebrate m6A methyltransferases, consider including METTL5 and ZCCHC4 or specify the discussion to non-rRNA m6A methyltransferases.

***RESPONSE:*** *As you suggested, we specifically mentioned the non-rRNA m6A methyltransferases (writers) in the introduction (line 69).*

- In Figure 4A, specify on the plot that adjusted p-values are shown.

***RESPONSE:*** *Done as suggested. Thanks!*

- In Figure 5E, include a legend indicating that the colors represent fold-change.

***RESPONSE:*** *Done as suggested. The red and blue colors refer to the fold changes that are upregulated and downregulated, respectively.*

- In Lines 336-338, where the authors refer to RNA abundance but plot fold change, please elaborate for clarity.

***RESPONSE:*** *RNA abundance was presented as the log_2_Fold Change(Mettl16/Control) in the X-axis according to the reference (Tan et al., 2023). We have clarified this in the Figure legend of* ***Figure 5DE****.*

- In Line 448-449, provide further clarification regarding the statement "the mRNA level was too low to be translated," potentially including references to support this phenomenon.

***RESPONSE:*** *Thank for your comment. We have rewritten this sentence as “the mRNA concentration (predicted to regulate ribosome occupancy and density) was so low that the protein expression could hardly be rescued (Nguyen et al., 2022)”.*

- In Lines 740-741, specify the parameters used to define "peaks."

***RESPONSE:*** *We have provided more details about m6A-seq analysis, and the parameters used to define “peaks” have been specified. Again, thank you very much for your scrutiny of our work.*

***REFERENCES:***

*Nguyen, H.L., Duviau, M.P., Laguerre, S., Nouaille, S., Cocaign-Bousquet, M., Girbal, L., 2022. Synergistic Regulation of Transcription and Translation in Escherichia coli Revealed by Codirectional Increases in mRNA Concentration and Translation Efficiency. Microbiol Spectr 10, e0204121.*

*Pendleton, K.E., Chen, B., Liu, K., Hunter, O.V., Xie, Y., Tu, B.P., Conrad, N.K., 2017. The U6 snRNA m(6)A Methyltransferase METTL16 Regulates SAM Synthetase Intron Retention. Cell 169, 824-835 e814.*

*Pusztai, A., Watt, W.B., 1971. Free-flow electrophoresis of proteins in phenol-containing solvents at various pH values. Biochim Biophys Acta 251, 158-163.*

*Su, R., Dong, L., Li, Y., Gao, M., He, P.C., Liu, W., Wei, J., Zhao, Z., Gao, L., Han, L., Deng, X., Li, C., Prince, E., Tan, B., Qing, Y., Qin, X., Shen, C., Xue, M., Zhou, K., Chen, Z., Xue, J., Li, W., Qin, H., Wu, X., Sun, M., Nam, Y., Chen, C.W., Huang, W., Horne, D., Rosen, S.T., He, C., Chen, J., 2022. METTL16 exerts an m(6)A-independent function to facilitate translation and tumorigenesis. Nat Cell Biol 24, 205-216.*

*Tan, X.S., Zheng, C.H., Zhuang, Y.H., Jin, P.P., Wang, F.C., 2023. The m6A reader PRRC2A is essential for meiosis I completion during spermatogenesis. Nature Communications 14.*

**Second round of review**

**Reviewer 1**

While I acknowledge the effects made by the authors, I still have further questions regarding their responses. See the attachment please.

Regarding splicing regulation by METTL16:

*There is a lack of figures illustrating alternative splicing changes for Mat2a (Line 273) following the loss of METTL16. Existing studies highlight METTL16's role in regulating MAT2A splicing in C. elegans (Mendel et al., Cell 2021) and human cells (Pendleton et al., Cell 2017) via either its methyltransferase or non-methyltransferase activities. Furthermore, I am wondering this Mat2a splicing changes in mice are due to METTL16's catalytic function or not?

*RESPONSE: Thank you for asking a good question. In the current study, we focused on identifying the alternative 3' splice site (A3SS) splicing event of Mat2a from our RNA-seq data, which differs from that reported in C. elegans and human cells. In addition, we found that the expression level of Mat2a mRNA was not altered in P10 Mettl16 cKO mouse testes compared to controls (see new Supplementary Figure S5C), suggesting that the spermatogenesis arrest in Mettl16 cKO mice may not directly correlated with the A3SS splicing of Mat2a in mice. Therefore, these changes in Mat2a splicing (A3SS) observed in mice may not be due to the catalytic function of METTL16. We have mentioned this possibility in the revised manuscript (see lines 494-500 with yellow highlighted).*

Reply: Thanks for the answer. I only have two minor points here. (1) Can the author use arrowheads to highlight the A3SS events in Mat2a 3’-UTR region please? I’ve no idea where to look in Figure S5C. (2) Typo. The figure legend should be 3’-UTR instead of 5’-UTR.

*U6 snRNA, a known METTL16 target essential for splicing, influences the splicing of specific 5' splice sites in yeast and plant studies (Ishigami et al., Nat Commun 2021; Parker et al., eLife 2022) but remains unexplored in mammalian cells. One of the interesting questions is that whether this 5' splice sites bias are also shown in mammalian cells. The author's proposal of METTL16 interaction with splicing regulator SF3B1/3 raises questions regarding splicing changes attributed to METTL16/U6 versus METTL16/SF3B. Utilizing established cell lines expressing catalytic mutant

METTL16 could help differentiate splicing changes influenced by its catalytic versus non-catalytic functions.

*RESPONSE: Thank you for raising this interesting question. We indeed identified 322 alternative 5’ splice site (A5SS) events in P10 Mettl16 cKO testis as shown in Fig. 4E, suggesting that the 5' splice site bias also occurred in mammalian cells.*

*In addition, we agree with your suggestion that in vitro cell lines expressing catalytic mutant METTL16 could help differentiate alternative splicing influenced by its catalytic or non-catalytic functions. We thus redesigned the minigene pcDNA3.1-Ddb2 and cotransfected it with METTL16 wild-type or catalytic mutant vectors in 293T cells. The results showed that in vitro splicing regulation was different from that in vivo, and the METTL16 catalytic mutant seemed to work as well as the METTL16 wild-type. We have added the new data in Supplementary Figure S5D-E.*

Reply: I appreciate the efforts made to address my comments. However, I feel that my queries remain unanswered. Specifically, I would appreciate a more detailed analysis of the 5’ splice site (A5SS) usage. Could the authors please examine which motifs or patterns of A5SS are increasingly or decreasingly utilized in the Mettl16 conditional knockout (cKO). This analysis could follow the methodologies used in the studies by Ishigami et al. (Nat Commun 2021) and Parker et al. (eLife 2022), where A5SS were shown to be potentially regulated via METTL16/U6 axis. I also hope the authors could discuss the splicing changes mediated by METTL16 in the Discussion regarding METTL16/U6 axis versus METTL16/SF3B axis.

*Via biochemistry experiments, the authors found that METTL16 interacts with MEIOC/YTHDC2/RBM46. However, the evidence for this interaction functionally regulating transcription and translational is lacking in this manuscript.

*RESPONSE: Thank you for your comment. We agree with the importance of functional interaction among METTL16 and MEIOC/YTHDC2/RBM46 at the transcriptional and translational levels, but this seems to be beyond the scope of the current research.*

*Based on the present study, it is clear that in P10 testis, METTL16 interacts with RNA close to MEIOC/YTHDC2/RBM46 binding sites and that METTL16 deletion can lead to decreased expression levels of YTHDC2 and MEIOC but does not affect the protein level of RBM46. We will follow up on the study for this interaction functionally regulating transcription and translational among these complexes in the future.*

Reply: I appreciate the efforts made to address my comments. At least to me, it seems that the identified interaction between METTL16 and MEIOC/YTHDC2/RBM46 does not help to understand the mechanisms that METTL16 act in spermatogenesis. The authors claim this interaction as a critical transcriptional and translational regulator responsible for proper cell cycle and meiosis initiation progress (Line 412-413). In fact, there is NO evidence provided in this study which genes are regulated via this interaction. This is not beyond the scope of this study if the authors aim to study the mechanisms that METTL16 regulates splicing and translation. Otherwise, the section could be removed from this study.

Meanwhile, I have several minor points that I hope can be helpful for this study.

*The authors observed that m6A-sequencing showed that ablation of METTL16 caused upregulation of the m6A-enriched transcripts and downregulation of the m6A depleted transcripts. However, the authors also observed that METTL16 knockout causes very minor global m6A changes (by MS and m6A-seq). (1) Can the authors plot m6A levels on U6 RNA with and without METTL16? This could be used as a proof-of concept. (2) As the majority of m6A on mRNA are not installed by METTL16 and they stayed unchanged with and without METTL16, how does the expression level of m6Aenriched transcripts are upregulated whilst m6A-depleted transcripts are downregulated due to loss of METTL16?

*RESPONSE: Thank you for raising these comments and good suggestions. (1) We have tried to plot m6A levels on U6 RNA, but unfortunately, we have failed because our m6A-seq data mainly detects the m6A modifications on mRNA, not on small nuclear RNA. (2) Following your comment, we have used P8 Mettl16 cKO testis to detect the expression level of other classic m6A installed proteins, including METTL3, METTL14, ALKBH5, and YTHDF2. The results showed that the expression levels of these proteins did not change in the P8 Mettl16 cKO testis compared to that of controls (see new Figure 5G H). Nevertheless, our m6A-seq data still detected many m6A differentially modified genes, suggesting there may be potential m6A-installed pathways regulated by METTL16 directly that require elucidation in the future.*

Reply: if the m6A-seq can not be used for U6 m6A measurement, the authors need to use alternative ways to verify that m6A on U6 is gone, e.g. RIP-qPCR, or SELECT. This becomes essential to prove that METTL16 is functionally gone, as the METTL16 WB is not completely gone due to mixed population of cells.

**Reviewer 3**

For the revised version of their manuscript, the authors carried out an impressive number of additional experiments and have taken into account most of my comments and suggestions.

I have one finale comment related to my point 2 (NB. from Rev3): to my observation that P10 whole testes were used to perform most NGS analyses, despite the fact that germ cell composition is not the same in KO and CTL at this stage (cf figures 2 and 3). The authors replied that they “have tried to purify the spermatogonia using P8-10 testes multiple times, but unfortunately, technically failed to collect enough cells for sequencing (ribo-seq, m6A-seq) due to the limited Mettl16 cKO testes available during each cell purification process.”

I understand the technical difficulties to perform challenging experiments on P8 rather than on P10 whole testes. Since the validation of DEGs by RT-qPCR was performed on purified Kit+ spermatogonia (Figure 4 with now a detailed legend), it is convincing that the genes they quantified by RT-qPCR are bona fide DEGs from METTL16 KO. There is nevertheless a potential bias in their sequencing results performed on KO P10 testis that I think should be mentioned in the manuscript.

Regarding RT-qPCR statistical analyses, it would be appropriate to correct the t-test for multiple testing (if it has not been done).

Minor typo in the Abstract :
Our findings support

**Reviewer 4**

The authors adequately addressed all comments and substantially improved the quality and clarity of the manuscript, which is ready for publication.

**Authors Response**

**Point-by-point responses to the reviewers’ comments:**

Reviewer reports:
Reviewer #1: While I acknowledge the effects made by the authors, I still have further questions regarding their responses. See the attachment please.

Regarding splicing regulation by METTL16:
*There is a lack of figures illustrating alternative splicing changes for Mat2a (Line 273) following the loss of METTL16. Existing studies highlight METTL16's role in regulating MAT2A splicing in C. elegans (Mendel et al., Cell 2021) and human cells (Pendleton et al., Cell 2017) via either its methyltransferase or non-methyltransferase activities. Furthermore, I am wondering this Mat2a splicing changes in mice are due to METTL16's catalytic function or not?

*RESPONSE: Thank you for asking a good question. In the current study, we focused on identifying the alternative 3' splice site (A3SS) splicing event of Mat2a from our RNA-seq data, which differs from that reported in C. elegans and human cells. In addition, we found that the expression level of Mat2a mRNA was not altered in P10 Mettl16 cKO mouse testes compared to controls (see new Supplementary Figure S5C), suggesting that the spermatogenesis arrest in Mettl16 cKO mice may not directly correlated with the A3SS splicing of Mat2a in mice. Therefore, these changes in Mat2a splicing (A3SS) observed in mice may not be due to the catalytic function of METTL16. We have mentioned this possibility in the revised manuscript (see lines 494-500 with yellow highlighted).*

Reply: Thanks for the answer. I only have two minor points here. (1) Can the author use arrowheads to highlight the A3SS events in Mat2a 3’-UTR region please? I’ve no idea where to look in Figure S5C. (2) Typo. The figure legend should be 3’-UTR instead of 5’-UTR.

*RESPONSE: Thank you for the good suggestions. We have highlighted the A3SS events in the Mat2a 3’-UTR region using red arrows and corrected the typo in the new Figure S5C.*

*U6 snRNA, a known METTL16 target essential for splicing, influences the splicing of specific 5' splice sites in yeast and plant studies (Ishigami et al., Nat Commun 2021; Parker et al., eLife 2022) but remains unexplored in mammalian cells. One of the interesting questions is that whether this 5' splice sites bias are also shown in mammalian cells. The author's proposal of METTL16 interaction with splicing regulator SF3B1/3 raises questions regarding splicing changes attributed to METTL16/U6 versus METTL16/SF3B. Utilizing established cell lines expressing catalytic mutant METTL16 could help differentiate splicing changes influenced by its catalytic versus non-catalytic functions.
 *RESPONSE: Thank you for raising this interesting question. We indeed identified 322 alternative 5’ splice site (A5SS) events in P10 Mettl16 cKO testis as shown in Fig. 4E, suggesting that the 5' splice site bias also occurred in mammalian cells.*
*In addition, we agree with your suggestion that in vitro cell lines expressing catalytic mutant METTL16 could help differentiate alternative splicing influenced by its catalytic or non-catalytic functions. We thus redesigned the minigene pcDNA3.1-Ddb2 and cotransfected it with METTL16 wild-type or catalytic mutant vectors in 293T cells. The results showed that in vitro splicing regulation was different from that in vivo, and the METTL16 catalytic mutant seemed to work as well as the METTL16 wild-type. We have added the new data in Supplementary Figure S5D-E.*

Reply: I appreciate the efforts made to address my comments. However, I feel that my queries remain unanswered. Specifically, I would appreciate a more detailed analysis of the 5’ splice site (A5SS) usage. Could the authors please examine which motifs or patterns of A5SS are increasingly or decreasingly utilized in the Mettl16 conditional knockout (cKO). This analysis could follow the methodologies used in the studies by Ishigami et al. (Nat Commun 2021) and Parker et al. (eLife 2022), where A5SS were shown to be potentially regulated via METTL16/U6 axis. I also hope the authors could discuss the splicing changes mediated by METTL16 in the Discussion regarding METTL16/U6 axis versus METTL16/SF3B axis.

*RESPONSE: Thank you for your constructive feedback and insightful suggestions. We appreciate your interest in our manuscript and the specific queries raised regarding the 5’ splice site (A5SS) analysis in the Mettl16 conditional knockout (cKO) mice. Based on your comments, we acknowledge the importance of examining A5SS motifs and patterns influenced by METTL16 depletion. However, we apologize that performing a comprehensive analysis similar to these studies within the requested timeframe is a significant challenge for us due to limited resources and time constraints. In reviewing the literature and considering your query, we note that the role of the m6A modification on U6 and splicing regulation in reproductive tissues remains unknown, which prompted us to further investigate its function during reproductive biology in the future. In addition, following your suggestion, we have made the discussion of splicing changes mediated by METTL16 in the context of the METTL16/U6 axis versus the METTL16/SF3B axis in the Discussion section of revised manuscript.*

*Via biochemistry experiments, the authors found that METTL16 interacts with MEIOC/YTHDC2/RBM46. However, the evidence for this interaction functionally regulating transcription and translational is lacking in this manuscript.
*RESPONSE: Thank you for your comment. We agree with the importance of functional interaction among METTL16 and MEIOC/YTHDC2/RBM46 at the transcriptional and translational levels, but this seems to be beyond the scope of the current research. Based on the present study, it is clear that in P10 testis, METTL16 interacts with RNA close to MEIOC/YTHDC2/RBM46 binding sites and that METTL16 deletion can lead to decreased expression levels of YTHDC2 and MEIOC but does not affect the protein level of RBM46. We will follow up on the study for this interaction functionally regulating transcription and translational among these complexes in the future.*

Reply: I appreciate the efforts made to address my comments. At least to me, it seems that the identified interaction between METTL16 and MEIOC/YTHDC2/RBM46 does not help to understand the mechanisms that METTL16 act in spermatogenesis. The authors claim this interaction as a critical transcriptional and translational regulator responsible for proper cell cycle and meiosis initiation progress (Line 412-413). In fact, there is NO evidence provided in this study which genes are regulated via this interaction. This is not beyond the scope of this study if the authors aim to study the mechanisms that METTL16 regulates splicing and translation. Otherwise, the section could be removed from this study.

*RESPONSE: Thank you for your comments. We agree with you that our study does not provide direct evidence of the genes regulated by the interaction between METTL16 and MEIOC/YTHDC2/RBM46. To avoid any overstatement, we have revised the conclusions to only claim potential co-regulation of target genes rather than definitive regulation in the revision. This adjustment aims to maintain clarity while acknowledging the possibility of co-operative interactions affecting biological processes. We hope that our revision meets your expectations and improves the clarity and relevance of the manuscript.*

Meanwhile, I have several minor points that I hope can be helpful for this study.
*The authors observed that m6A-sequencing showed that ablation of METTL16 caused upregulation of the m6A-enriched transcripts and downregulation of the m6A depleted transcripts. However, the authors also observed that METTL16 knockout causes very minor global m6A changes (by MS and m6A-seq). (1) Can the authors plot m6A levels on U6 RNA with and without METTL16? This could be used as a proof-of concept. (2) As the majority of m6A on mRNA are not installed by METTL16 and they stayed unchanged with and without METTL16, how does the expression level of m6Aenriched transcripts are upregulated whilst m6A-depleted transcripts are downregulated due to loss of METTL16?

*RESPONSE: Thank you for raising these comments and good suggestions. (1) We have tried to plot m6A levels on U6 RNA, but unfortunately, we have failed because our m6A-seq data mainly detects the m6A modifications on mRNA, not on small nuclear RNA. (2) Following your comment, we have used P8 Mettl16 cKO testis to detect the expression level of other classic m6A installed proteins, including METTL3, METTL14, ALKBH5, and YTHDF2. The results showed that the expression levels of these proteins did not change in the P8 Mettl16 cKO testis compared to that of controls (see new Figure 5G H). Nevertheless, our m6A-seq data still detected many m6A differentially modified genes, suggesting there may be potential m6A-installed pathways regulated* *by METTL16 directly that require elucidation in the future.*

Reply: if the m6A-seq can not be used for U6 m6A measurement, the authors need to use alternative ways to verify that m6A on U6 is gone, e.g. RIP-qPCR, or SELECT. This becomes essential to prove that METTL16 is functionally gone, as the METTL16 WB is not completely gone due to mixed population of cells.

*RESPONSE: We appreciate your comments and agree with your suggestions, however, we are unable to collect enough fresh P8 Mettl16 cKO mouse testes for RIP-qPCR analysis within the limited timeframe of the revision. Furthermore, both the current histological staining results and the m6A-seq data effectively support our conclusion regarding the functional loss of METTL16 during spermatogenesis.*

Reviewer #3:
For the revised version of their manuscript, the authors carried out an impressive number of additional experiments and have taken into account most of my comments and suggestions.

I have one finale comment related to my point 2 (NB. from Rev3): to my observation that P10 whole testes were used to perform most NGS analyses, despite the fact that germ cell composition is not the same in KO and CTL at this stage (cf figures 2 and 3). The authors replied that they “have tried to purify the spermatogonia using P8-10 testes multiple times, but unfortunately, technically failed to collect enough cells for sequencing (ribo-seq, m6A-seq) due to the limited Mettl16 cKO testes available during each cell purification process.”

I understand the technical difficulties to perform challenging experiments on P8 rather than on P10 whole testes. Since the validation of DEGs by RT-qPCR was performed on purified Kit+ spermatogonia (Figure 4 with now a detailed legend), it is convincing that the genes they quantified by RT-qPCR are bona fide DEGs from METTL16 KO. There is nevertheless a potential bias in their sequencing results performed on KO P10 testis that I think should be mentioned in the manuscript.
RESPONSE: Thank you again for your comments. We have mentioned the potential bias in sequencing as suggested.

Regarding RT-qPCR statistical analyses, it would be appropriate to correct the t-test for multiple testing (if it has not been done).
*RESPONSE: We appreciate your suggestion and will ensure that our statistical approach follows best practice to maintain the statistical significance and reliability of our results. In this study, we used the t-test for statistical analysis in our RT-qPCR experiments, with each treatment group having its corresponding control group for comparison. This choice was made due to the design of our experiment, where the t-test is appropriate for evaluating mean differences between two groups. If multiple comparisons were performed, we would consider appropriate corrections such as Bonferroni or Benjamini-Hochberg procedures to ensure control of the type I error rate.*

Minor typo in the Abstract :
Our findings support
*RESPONSE: We have corrected the typos in the Abstract. Thanks!*

Reviewer #4: The authors adequately addressed all comments and substantially improved the quality and clarity of the manuscript, which is ready for publication.
*RESPONSE: Thank you very much for your appreciation of this revised manuscript.*
